# Effects of mobility on dialect change: Introducing the linguistic mobility index

**DOI:** 10.1371/journal.pone.0300735

**Published:** 2024-04-16

**Authors:** Péter Jeszenszky, Carina Steiner, Adrian Leemann

**Affiliations:** 1 Center for the Study of Language and Society (CSLS), University of Bern, Bern, Switzerland; 2 Institute of Germanic Languages and Literatures, University of Bern, Bern, Switzerland; 3 German Seminar, University of Zurich, Zurich, Switzerland; Royal Holloway University of London, UNITED KINGDOM

## Abstract

Increased geographical mobility prompts dialectologists to factor in survey participants’ exposure to linguistic variation in their research. Changing mobility patterns (e.g. longer-distance commuting; easier relocation to distant places for work, study or marriage) have caused linguistic connections to become much more diverse, potentially contributing to the acceleration of dialect change. In this methodological work we propose the *Linguistic Mobility Index* (LMI) to estimate long-term exposure to dialectal variation and thereby to provide a reference of “localness” about survey participants. Based on data about a survey participant’s linguistic biography, an LMI may comprise combinations of influential agents and environments, such as the dialects of parents and long-term partners, the places where participants have lived and worked, and the participants’ level of education. We encapsulate the linguistic effects of these agents based on linguistic differences, the intensity and importance of the relationship. We quantify the linguistic effects in three steps. 1) The linguistic effect of an agent is represented by a linguistic distance, 2) This linguistic distance is weighted based on the intensity of the participant’s exposure to the agent, and 3) Further weighted according to the relationship embodied by the agent. LMI is conceptualised and evaluated based on 500 speakers from 125 localities in the Swiss German Dialects Across Time and Space (SDATS) corpus, and guidance is provided for establishing LMI in other linguistic studies. For the assessment of LMI’s applicability to other studies, four LMI prototypes are constructed based on the SDATS corpus, employing different theoretical considerations and combinations of influential agents and environments to simulate the availability of biographical data in other studies. Using mixed-effects modelling, we evaluate the utility of the LMI prototypes as predictors of dialect change between historic and contemporary linguistic data of Swiss German. The LMI prototypes successfully show that higher exposure to dialectal variation contributes to more dialect change and that its effect is stronger than some sociodemographic variables that are often tested for affecting dialect change (e.g. sex and educational background).

## Introduction

In this paper, we argue that quantifying exposure to dialectal or linguistic variation at speaker level may provide researchers with a new approach for investigating language variation and change. Increased mobility jeopardises the validity of region as the primary determinant of linguistic variation [1], due to mobility-induced dialect change. Mobility leads to a potential increase in contact, exposing individuals to linguistic variation, and the intensity of this exposure plays a key role in language change [2]. Due to this increasing exposure to linguistic variation, it is indispensable to address the mobility of participants in linguistic surveys [3,4]. To this end, this methodological paper introduces the approach of the *Linguistic Mobility Index* (LMI), a heuristic to estimate individuals’ exposure to potential linguistic influences through examining long-term mobility patterns in their linguistic biographies. While our primary focus lies in dialectology and sociolinguistics, we encourage the broader application of the linguistic mobility approach across various linguistic.

Mobile behaviour potentially results in contact with peers in different localities and in long-term exposure to linguistic variation (e.g. through regular contact, such as via commuting or relocation). Previous dialectological and sociolinguistic studies have researched mobility and exposure to contact in relation to different linguistic aspects (e.g. [1–3,5–9]). David Britain’s extensive research on the topic underscores the crucial influence of mobility on dialectal variation. His exploration of “sedentarism” and “nomadism” reveals that spatial mobility significantly shapes linguistic diversity within dialects [3]. In addition, his research on spatial dimensions, language change and diffusion in urban and rural contexts [7] and with regards to contact and divergence around dialect boundaries [10] highlight the nuanced impact of mobility on dialectological research. Similar to his holistic views, we regard linguistic mobility as reflecting the combined potential effects of the places visited or contacts made (i.e. contact with peers from these places) on an individual’s dialect. Thus, a linguistically mobile person is characterised by activities that bring them into contact with linguistically different localities, such as multiple relocations throughout their lives, routinely commuting for study, work or other regular activities, or having family ties in such localities. In contrast, a linguistically non-mobile person would gain most of their linguistic influences from the inhabitants of the locality in which they grew up.

The constant increase in people’s geographical mobility over the last century has strengthened the potential impact of diverse linguistic contacts on dialect use [11–13]. This linguistic change caused by the intensification of dialect contact among speakers from a larger number of places has been framed in dialectology as a function of the general mobility patterns of the population, conceptualised, for example, as the wave model of language change [14] and the linguistic gravity model [9]. However, little quantitative research has been conducted on the effects of mobility at the speaker level, partly because surveys traditionally focused on capturing variation elicited from non-mobile old rural males and females (NORMs and NORFs, cf. [15]). Of the available studies on geographical mobility of individuals, Chambers [1] devised the *Regionality Index* (*RI*) as a function of the participant’s birthplace, their residence from 8 to 18, the current residence and their parents’ birthplace. The result is also weighted by proximity to the home region. In his study [1], he tested *RI* in Quebec City and the Golden Horseshoe region on three lexical variables and showed significant differences in lexical choices with regards to levels of *RI*. Beaman [5] studied the role of mobility and local orientation in the attrition of Swabian German through observing dialect change within the lifespan of individuals. She quantified the mobility of her participants by combining ‘residential dispersion’, the number of moves, and ‘residential distance’, the distance of the locations lived at from the birthplace, both weighted by the number of years spent in each location. She has shown that while mobility does not make a difference in her 1982 survey, in the 2017 survey, women with high mobility have lower probability of speaking dialect. Bowie [6] observed the retention of phonological forms and studied the effect of being exposed to a second dialect quantified by the time that elapsed since his adult speakers moved away from Waldorf, Maryland. For some linguistic variables, he found larger effects the longer the speaker had been gone. In a similar constellation, Regan [8] studied the change in the perceived socioeconomic status of words, based on the number of years spent away from Lepe, Andalusia, Spain. Moreover, Chambers’ *RI* [1] was applied in studies to account for the extent to which individuals could represent local communities [16–18].

In terms of quantifying exposure to linguistic variation through contact, research has focused on the relation between language change and the most important linguistic influences in individuals’ lives. An individual’s linguistic heritage is viewed as coming from the speech of their parents and primary caregivers, who have a foundational influence on their dialect, especially in early childhood before large-scale exposure to older peers (e.g. [19–22]). This foundation is then strongly shaped by other intense contacts, including relatives, peers during childhood and adolescence [22], such as at school [23,24], partners [25,26]; and contacts within the workplace and other communities as adults [7,26,27]. All of these influential agents and environments are important to consider when studying linguistic mobility.

To date, quantitative measures of the linguistic exposure of individuals to other dialects have not been determined based on aggregating biographical information. Instead, quantification of linguistic mobility has focused on its foundational aspects, such as assigning rates based on time spent away from a reference locality [5,6,8]. The LMI approach addresses this research gap by systematically constructing an index based on multiple linguistically influential agents and environments that can be extracted from sociodemographic information recorded in linguistic surveys. Doing so, the LMI integrates linguistic biography data into a single value representing exposure to linguistic variation.

We demonstrate the implementation of the LMI approach using data from 500 survey participants recorded in the SDATS corpus (*Swiss German Dialects Across Time and Space* [28]). Four LMI prototypes are constructed which simulate the availability of biographical data in other dialect surveys, thereby testing the applicability and flexibility of the LMI approach.

We evaluate the usefulness and relevance of the implemented LMI by predicting dialect change while controlling for variation in sociodemographic variables frequently used to assess language change. Other studies have included in such tasks for instance, depending on the design of the linguistic survey, gender (e.g. [29,30]), educational background (e.g. [31]), urbanity and social networks (e.g. [32–36]). Our evaluation compares the performance of these variables to the performance of four LMI prototypes as predictors in mixed-effects models. The predicted dialect change rate is calculated based on ten lexical variables, using historical linguistic data from the *Sprachatlas der deutschen Schwei*z (SDS [37]), the most comprehensive data collection on Swiss German dialects, recorded in the 1950s and ‘60s, and contemporary data from the SDATS project. We expect the model results to confirm that linguistically mobile speakers have higher rates of dialect change, while non-mobile speakers have lower rates.

The remainder of this paper is structured as follows. After outlining the general construction of LMI, we present the implementation of LMI on the example of the SDATS corpus and evaluate four LMI prototypes in mixed-effects models. In addition, we briefly address the question of regionality and urbanity with respect to mobility’s effects on language change. After presenting and discussing the results of the models, we identify the limitations of LMI and provide further recommendations for the application of LMI in other research areas.

## Methods

### Introducing the linguistic mobility index

The core concept of LMI is the aggregation of the linguistic effects of social connections and influential agents or environments (henceforth referred to as *‘agents’*) affecting a participant throughout their life. These *agents* may be people and groups that the participant has been exposed to. LMI estimates a summary of the potential effects accumulated throughout the life of the participant, weighted according to the participant’s *relationship* to the agent and the *intensity of exposure* to that contact. Linguistic surveys with a controlled selection of participants usually elicit some sociodemographic information about their participants and about some of the influential agents and situations in question. This information may be used to quantitatively estimate the potential linguistic effects on a participant.

Dialectological surveys typically assign each participant to a reference locality or linguistic variety. Similarly, a location could be assigned to each influential agent. The potential linguistic effect may be calculated based on the linguistic distance (we use this term to quantify linguistic differences across survey sites in the spirit of dialectometry, e.g. [38]) of the participant to the agents’ assigned locations. The intensity of the participant’s exposure to these locations, and their relationship, is expressed based on information elicited from survey questionnaires or other biographical data (e.g. the type of social connection that exposed them to the location, or the time they spent there). In comparison to Chambers’ RI, which measures the extent to which an individual has been exposed to a reference locality, LMI measures the potential effect of the linguistic variation pertaining to places that the participant encountered outside their reference locality.

A linguistically influential agent’s or environment’s effect on a survey participant can be thus generally estimated based on the following steps, given a collection of linguistic material and questionnaire data associated with the participants and the agents.

1. **Calculating linguistic distance**: The basis of LMI is linguistic distance, which is a quantitative estimate of the difference between the linguistic varieties that pertain to the participant and to the agent (e.g. the accumulated difference of a number of lexical, phonetical and morphosyntactic variables between the participant’s and the agent’s reference locality).
2. **Weighting based on the intensity of the exposure**: Delivering a more exact estimation of the agent’s effect may be based on available questionnaire data regarding the participant’s exposure to the agent (e.g. age at the time of contact, duration and frequency of the contact). The linguistic distance is multiplied by this weight.
3. **Weighting based on the participant’s relationship to the agent**: Determining the typical long-term influence of the kinds of agents involved on the linguistic profile on an individual. In this step theoretical decisions may play a more important role, as to determine what role parents, school, workplace, or other kinds of agents that the linguistic survey has data about, typically plays in a community. A weight is devised for each kind of agent, and the result of the previous step is multiplied by it.

A participant’s LMI is then created as an aggregation of those agents’ estimated effects that the researchers wish to investigate in their study. The larger the resulting LMI, the more long-term effect the participant has potentially received due to the dialectal variation they were exposed to.

### Implementing the LMI approach on Swiss German dialect data

Before embarking on the implementation of the LMI approach on Swiss German dialects, it is important to take note of the specific linguistic situation of Swiss German. In the diglossic context of Switzerland, dialects enjoy a higher prestige in comparison to Standard German (e.g. [39,40]). This is not just covert prestige, but using dialects is seen as an identity-establishing language tactic across all German-speaking Swiss regions and social classes [40]. Although some ‘levelling’ is occurring in Swiss German dialects [20,41], Swiss German speakers (practically all German speakers born or raised in Switzerland) use their own dialects colloquially and almost never use Standard German as a lingua franca between speakers of various dialects. While Swiss German speakers are constantly exposed to Standard German from an early age, such as in school and via the media, speaking Standard German is expected only in situations that require a standardised, widely understood form of communication, including academic or professional environments, government institutions, written communication, and in interactions with speakers from Germany and Austria, and with non-native speakers. The diverse topography and the (historical) administrative structure of Switzerland often determine mobility flows and cultural orientations, e.g. fastest paths follow valleys, and natural and man-made boundaries limit communication and cultural exchange. This contributes to the regional diversity of the dialects. In consequence, spatial patterns are expected in linguistic mobility and, correspondingly, in dialect change, such as cascade patterns often manifesting themselves along the mobility paths [7,9,14], which are often defined by topographic features.

The LMI framework has been implemented using sociodemographic information obtained in the questionnaire that the participants of the SDATS survey filled out, answering questions related to various long-term linguistic influences. This information is used for establishing the aforementioned agents for this study, which are detailed in the following sections. Importantly, SDATS was collected to serve multiple dialectological and sociolinguistic research purposes and the design of the present study had no direct impact on the linguistic variables and the biographical data recorded in the SDATS survey or its questionnaire. We believe that researchers wishing to implement a similar index would be in a similar situation, having data from more general purpose and/or legacy dialect surveys.

#### Swiss German Dialects Across Time and Space (SDATS)

The SDATS survey was conducted in 2020–2021 across 125 localities in German-speaking Switzerland, which form a subset of the 625 SDS survey localities [42]. SDS [37] is the largest complete dialectal survey of Swiss German and served as the basis for linguistic distance calculations, as detailed below. From each of these localities, we used data from four participants for this study: two older (65+ years old) and two younger speakers (20–35 years old), with one male and one female speaker in both age cohorts. For every speaker, one parent came from the region of the reference locality, and the speakers themselves grew up in and lived most of their lives there. We recorded a mix of educational backgrounds without considering occupations. Further, their daily travel time was required to not exceed the Swiss average of approximately two hours. After a dialect interview, the speakers filled out an unsupervised online questionnaire consisting of over 300 items eliciting demographic information (age, gender, education, and occupation), as well as detailed mobility profiles, language biographies, information on social networks (relatives and peers), personality (the Big Five personality traits) and language attitudes (cf. [43] for details).

The SDATS participants were properly instructed and indicated their consent to participate by signing an appropriate consent form, approved by the Legal Services Office of the University of Bern. The participants explicitly consented to the anonymous analysis of the data they provided and were informed about the applicability of the Data Privacy Act of the Canton of Berne (Datenschutzgesetz des Kantons Bern–KDSG; BSG 152.04, from 19.02.1986). This procedure of collecting and analysing anonymous user data conforms to the regulations of the Bern cantonal ethics committee (https://www.gsi.be.ch/de/start/ueber-uns/kommissionen-gsi/ethikkommission.html) and the accompanying federal act on research involving human beings in Switzerland (https://www.fedlex.admin.ch/eli/cc/2013/617/en). For this reason, we did not seek further ethical approval from cantonal or federal institutional bodies.

#### The steps of implementing LMI on SDATS data

**Fig 1:**
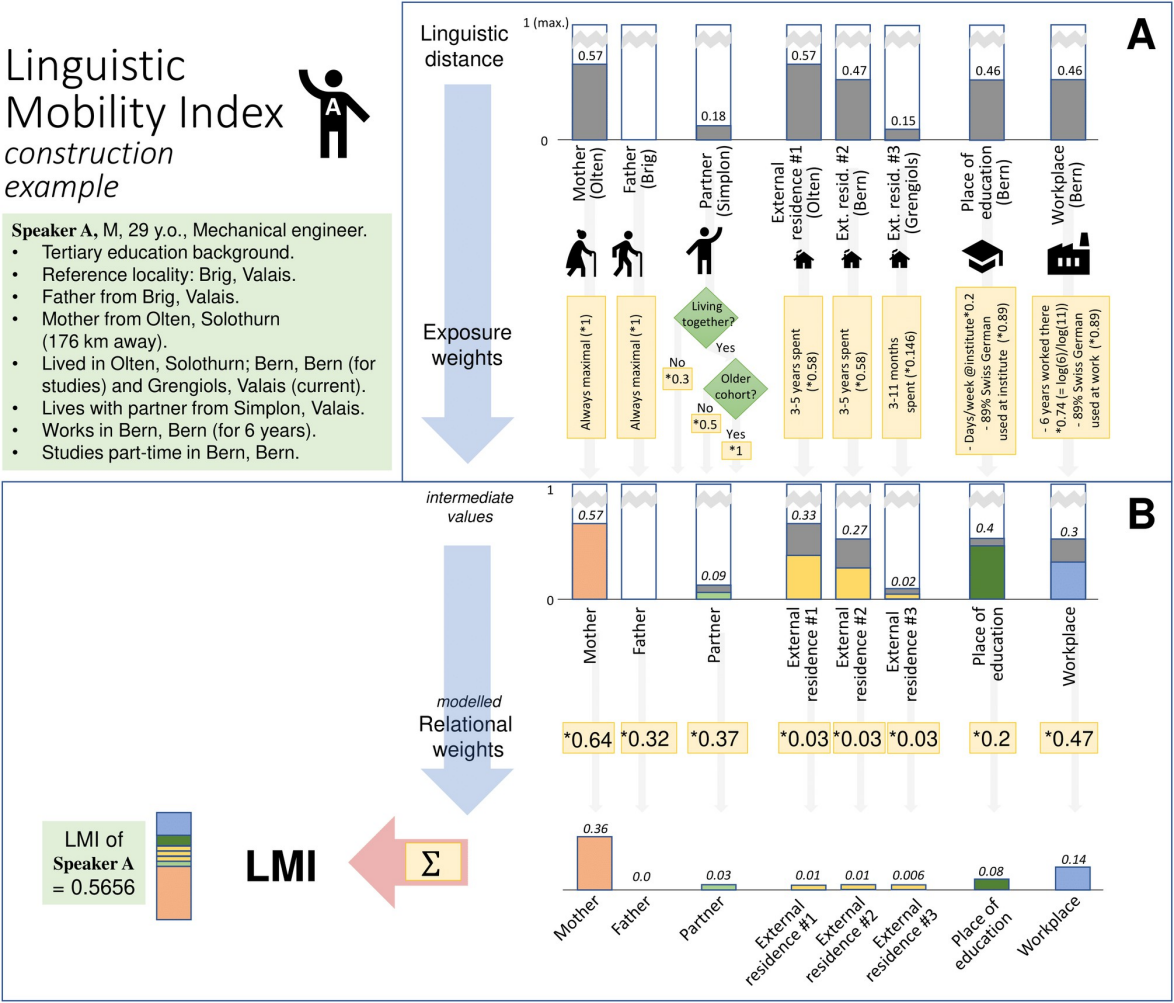
Example calculation of LMI components, demonstrating the three main steps of the process: Linguistic distance calculation, exposure weights and relational weights. The linguistic distance values are multiplied by the exposure weights and by the relational weights, and then the results are summed.

Using the example of Speaker A in the following sections, we will demonstrate the construction of LMI based on SDATS questionnaire data. Fig 1 also illustrates the steps followed in constructing LMI. Information on the data preparation, construction of LMI and data modelling can be accessed in the Supplementary Material, along with the corresponding R source code in S1 Appendix and S2 Appendix, at https://osf.io/hfbpk/.

Implementing the LMI approach started by determining the relevant linguistically influential agents and environments that could be retrieved based on the speakers’ SDATS questionnaire data including various amounts of information on the agents. We constructed the following agents: mother, father, long-term partner (henceforth: partner), residence where a speaker has lived outside the reference locality (henceforth: external residence), place of ongoing education and current workplace. We opted for these agents based on the availability of biographical data in the SDATS questionnaire that was comparable across most participants. The agents are considered to represent the linguistic variation of places and people to which an individual has the most contact throughout their lives. The between-speaker variation, however, results in a scenario where not all agents can be established for each speaker (e.g. pensioners without information on workplace, young people without a partner). For further kinds of biographical data that we did not include, consult S1 Appendix (Section 1). In what follows, we go through the aforementioned three steps of estimating agents’ effect on survey participants.

#### Linguistic distance

We quantified an agent’s potential linguistic effect on a speaker by calculating a linguistic distance between them. This involves assigning a locality to the agent and calculating the linguistic distance between this locality and the speaker’s reference locality, using SDS dialect data.

We assigned an SDS survey locality (*n* = 565) to each agent through geocoding. The locality names associated with the agents, as recorded in the SDATS questionnaire (e.g. Speaker A’s workplace is Bern), were matched to geographical coordinates by means of geocoding, in R [44] (version 4.0.4.), using the *tidygeocoder* package [45] (version 1.0.3.). Then, solving point-in-polygon problems using the Voronoi polygons of SDS localities, the closest SDS survey locality was assigned to the agent, with the help of the *sf* package [46] (version 1.0–2.). In the example in Fig 1, Speaker A named Bern as the locality of his workplace. Geocoding this string returned the geographic coordinates of the de facto capital city, Bern. The point-in-polygon routine then determined that the coordinates are found in the Voronoi polygon of the corresponding SDS locality ‘Bern’.

As the next step, linguistic distances were determined between the agent’s locality and the reference locality of the speaker, based on dialect data from the SDS. These linguistic distances were calculated based on Goebl’s *Relative Identity Value* (*RIV*_jk_) [47], using the portion of the SDS variables which were digitised by Scherrer and his colleagues starting from 2008 [48] (289 linguistic variables: 107 phonetic, 118 morphosyntactic and 64 lexical variables). For any pairs of SDS localities, linguistic distance were calculated, similarly to [49], as follows. For every linguistic variable, phonetic, morphosyntactic or lexical alike, phonetically dissimilar variant categories were constructed (see Table 1 for examples). Within these variant categories, a further distinction was made between the phonetically similar subvariants that occur in SDS. When the variant categories between Locality *i* and Locality *j* are different, the linguistic distance grows by 1. Even when variant categories are identical but the subvariants differ, the linguistic distance increases to a lesser extent. The linguistic distance between any pair of localities is the accumulation of linguistic distances regarding the variables (divided by the *n* number of variables that had data for both localities in SDS). The linguistic distance between any two localities may thus span from 0 to 1 (i.e. 0 = linguistically identical and 1 = total linguistic discrepancy). At variable level this can be written as in Equation (1),

$$D_{ij}^{ling}=\frac{\Sigma D_{Q}}{n}$$

where *D*_*Q*_ is the number of differing variables regarding Localities *i* and *j*. Regarding the workplace of Speaker A (Bern) and his reference locality (Brig), the linguistic distance amounts to 0.46, which means that about half of the linguistic features are different across the two localities.

**Table 1:** Example for the calculation of a linguistic distance between two localities based on data in the Sprachatlas der deutschen Schweiz (SDS). A1, B1, B2 and C1 are variant categories, while A1.1, B1.1, B2.1, C1.1 and C1.2 are subvariants within the respective variant categories.

	*Variable A* *(Std*. *Ger*. *‘Pflaume’)* *(Eng*. *’plum‘)*	*Variable B* *(Std*. *Ger*. *‘hinauf’)* *(Eng*. *’onto’ [sth*.*])*	*Variable C* *(Std*. *Ger*. *‘schneiden’)* *(Eng*. *’to cut‘)*	*…*	*Variable N*
***Locality i*, *Speaker 1***	*A 1*.*1 (‘Pfluume’)*	*B 1*.*1 (‘ufe’)*	*C 1*.*1 (‘schääre’)*	*…*	*…*
***Locality j*, *Speaker 1***	*A 1*.*1 (‘Pfluume’)*	*B 2*.*1 (‘embruf’)*	*C 1*.*2 (‘schäärle’)*	*…*	*…*
*Linguistic distance per variable*	*0*	*1*	*0*.*1*		*…*

Due to the lack of data, there are some specific locality constellations for which we had to set linguistic distances as flat rates. The linguistic effect of the reference locality received a flat rate of 0 because it is known that the speakers in our sample (such as Speaker A from Brig in the above example) grew up in the reference locality. Having no additional information about the connection to one’s reference locality in the SDATS questionnaire, our best inference about the peer effect in the first 10–20 years of language acquisition is that it is identical to the speaker’s own (note that we consider parental effects separately). Similarly, places that are officially not German speaking receive a flat rate of 0 as we do not have data about whether anything related to such places influences the German language of the speaker. Based on our data, we cannot account for the effects of these places on the speakers’ German dialects, thus they do not increase the LMI. Places in Germany and Austria, representing Standard German, received a flat rate of 0.5 as linguistic distance, regardless of possible local dialectal influence. We do not have information about whether places in Austria and Germany impacted the speakers through local dialects or Standard German, and we lack dialect data comparable to SDS, for the calculation of linguistic distances. With this rather large flat rate we aim to acknowledge the pervasive influence of Standard German on Swiss German speakers. Its potential influence has led to the decision that exposure to Standard German should contribute to a higher linguistic mobility index. Among the 3000+ places geolocated for the agents, fewer than 200 places are in Austria and Germany. This also contributed to our decision to ignore the variation across the variety of German dialects and accents and to use a fixed rate.

#### Exposure weights

Using *exposure weights*, which quantify the intensity of the speaker’s contact with the agents, we fine-tuned the degree to which the effect of an agent is considered in LMI. This exposure weight is to be understood as the proportion to which the linguistic distance between the speaker and the agent is considered. The SDATS questionnaire recorded different parameters for each kind of agent, which we compiled into weights to quantify the agents’ linguistic influence on the speaker. Exposure weights are specific to the kinds of agents and environments, they depend on the available questionnaire items associated with the agent, and range from 0 to 1. Thus, they regulate the effect of these agents and environments across speakers, i.e. the agents (mother, father, external residence, partners, place of education and workplace) differ due to the different kinds of questionnaire data recorded in relation to them. For each agent, linguistic distance is multiplied by the exposure weight value (Fig 1).

As any research questionnaire might have different parameters associated with their potential agents, we gloss over the actual questionnaire items used for the agents in our implementation. Due to the decisive effects of parents on speakers’ dialects at the beginning of language acquisition, and lacking questionnaire data further specifying their relationship to the speaker, the linguistic distance associated with a parent that comes from outside the speaker’s reference locality receives the maximal exposure weight (i.e. 1). Regarding partners, the exposure weight differs depending on whether they live together (0.5) or whether the partner is mentioned among the three closest personal contacts of the speaker (0.3). For older speakers, living together with a partner may mean there is a higher certainty that they have been together for longer, accounting for more linguistic exposure. Therefore, the maximal weight is applied in these cases (i.e. 1). Residence outside the reference locality is weighted based on the number of years spent there, which was recorded as categories (e.g. 3–5 years). Based on the duration of residence, the weights increase logarithmically. The linguistic distance associated with the workplace receives a logarithmically growing weight, which reaches a maximum after 10 years having worked there (in this case, exact number of years was recorded in the questionnaire). Finally, for the place of education, the linguistic distance is weighted by the days per week spent at the institution. The value is weighted further in the case of workplace and place of education by the proportion of Swiss German and Standard German used at work (elicited as a self-rated percentage). Exposure to Standard German receives the flat rate of 0.5 in these calculations too. For Speaker A’s workplace, for example, the exposure weighting involves the logarithm of his 6 years having worked in Bern divided by the maximal amount considered (10 years), further weighted by the proportion of Swiss German and Standard German used at work. His 89% usage of Swiss German means a 0.89 multiplier to the linguistic distance of Bern (0.47), while a 0.11 multiplier to the linguistic distance with Standard German (0.5). For more details on exposure weighting, please see S1 Appendix.

#### Relational weights

Linguistic exposure also depends on the nature of the relationship an individual has to the locality (e.g. through an agent). We assume that a speaker has more intensive contact with the local linguistic variety through a person who is from that locality (e.g. a partner) than through an agent more loosely connected to the locality (e.g. studies, workplace) through which the speaker potentially encounters a more mixed linguistic community.

*Relational weights* serve to differentiate the effects of various kinds of agents. These weights remain unaffected by specific questionnaire items or the availability of data used for calculating exposure weights, which makes them relatively easy to implement in any study. For a specific kind of agent (e.g. partner, workplace, external residence), every speaker receives the same, constant relational weight as a multiplier. This way we can actively differentiate the effects of the workplace from the effect of other agents, such as parents. In the case of Speaker A, we first multiply the linguistic distance pertaining to his workplace with the exposure weight to account for the intensity of his workplace contacts (Fig 1A). Then we multiply this intermediate value by the relational weight associated with workplace as an agent (Fig 1B), in order to differentiate the effect of the workplace from the other agents.

For the current study, we implemented the relational weights through a modelling procedure. We set up the following four mixed-effects models for estimating relational weights. For each kind of agent, we will use their corresponding *β*-coefficients as relational weights.

- **M1**—Relational weight of place of education for those currently undertaking studies (number of speakers for whom the agent is relevant, *n* = 119);
- **M2**—Relational weight of partner for those with a partner identified in the questionnaire (*n* = 351);
- **M3**—Relational weight of workplace for those who indicated a workplace (*n* = 306); and
- **M4**—Relational weights of mother, father and external residence in a general model (*n* = 500).

The models use the unweighted linguistic distances between speaker–agent locality pairs as fixed effects alongside the control variables age, sex and educational background, all of which were used as criteria for the balanced participant selection in the SDATS survey (henceforth collectively referred to as *survey criteria*). The models predict a dialect change rate in ten lexical variables (also used for the evaluation of LMI, explained further below). A unique speaker identifier (n = 500) and the linguistic variable are used as random intercepts in the models. In terms of the missing values we used bootstrapped regression imputation to impute values for the partner’s and the workplace’s linguistic distance, with the help of the *mice* package [50] (version 3.14.0). Additionally, relational weights are also modelled separately for the older and younger age cohorts regarding workplace and external residence, due to the fact that the older cohort had had more chance to work and live longer in other places than the younger one. The resulting relational weights (*β*-coefficients of the model) are numerically indicated in Table 2 and highlighted in Fig 1B. With each of the models we used 10-fold cross-validation, using the *cv* package [51] (version 1.0.1) to detect possible over-fitting. The resulting average mean square error (for the general model above, *MSE* = 0.2378) being lower than the variance of the target variable (*σ*^*2*^ = 0.2291), i.e. the change from SDS, suggests that the model is providing predictions that are, on average, better than simply predicting the mean of the dialect change variable for all observations. For the details of this modelling procedure, see S1 Appendix.

**Table 2:** Composition of the four LMI prototypes.

Agent	LMI^A^ (Minimal)	LMI^B^ (Cumulative)	LMI^C^ (Comprehensive)	LMI^D^ (Cohort-based)
**Mother’s origin**	*LD* × 0.6351	*LD* × 0.6351	*LD* × 0.6351	*LD* × 0.6351
**Father’s origin**	*LD* × 0.317	*LD* × 0.317	*LD* × 0.317	*LD* × 0.317
**Partner’s origin**	-	*LD* × 0.3461	*LD* × *W*_*e*_ × 0.3461	*LD* × *W*_*e*_ × 0.3461
**External residence**	-	Σ *LD* × 0.0319	Σ *LD* × *W*_*e*_ × 0.0319	Older cohort: Σ *LD* × *W*_*e*_ × 0.0206
				Younger cohort: Σ *LD* × *W*_*e*_ ×0.1663
**Workplace**	-	*LD* ×0.4728	*LD* × *W*_*e*_ × 0.4728	Older cohort: *LD* × *W*_*e*_ × -0.8728
				Younger cohort: *LD* × *W*_*e*_ × 0.6061
**Place of education**	-	*LD* ×0.2023	*LD* × *W*_*e*_ × 0.2023	*LD* × *W*_*e*_ × 0.2023

*LD* stands for linguistic distance, *W*_*e*_ stands for exposure weight- Relational weights are indicated numerically, as they are constant for each kind of agent.

### Setting up the LMI prototypes

We constructed four LMI prototypes for all speakers, based on the linguistic distances pertaining to the agents, the exposure weights and the relational weights. The LMI prototypes represent different theoretical considerations and simulate possible scenarios in terms of available biographical data in dialectological survey projects. To this end, we take different subsets of the SDATS questionnaire data for each prototype, maintaining however, that the data is available for all 500 speakers. Each prototype bears a name and an abbreviation and Table 2 presents the components involved in each of them, along with the weights associated with the agents involved.

#### Minimal prototype (LMI^A^)

Constructing the minimal prototype simulates a scenario, where a hypothetical survey only records little background information about the speakers’ influences. We included the origin of the father and the origin of the mother, considering that many surveys include this information to account for authenticity. The agents are weighted only by relational weights, which simulates that there are no further parameters available about the agents. This way it is possible to test whether minimal background information may still be enough for the construction of a meaningful LMI.

#### Cumulative prototype (LMI^B^)

Surveys often elicit various pieces of background information (e.g. origin of parents, partners and peers, former places of residence, places of education and work) without gathering detailed information about the possible effect of these localities (e.g. [52]). The cumulative prototype sums up the linguistic distances from all agents in a linear manner, disregarding exposure weights and using only the relational weights. Maximising the effect of each agent (i.e. each locality) considered, this prototype basically cumulates influential agents and environments in a linguistic biography, assigning dialectal locations to them.

#### Comprehensive prototype (LMI^C^)

All the aforementioned agents’ effects (i.e. mother, father, partner, external residence, workplace and place of ongoing education) are included in LMI^C^, using exposure weights and the relational weights associated with them. The parameters of this prototype correspond to a scenario where a larger range of biographical data is elicited from which one could estimate exposure weights for some of the agents.

#### Cohort-based prototype (LMI^D^)

The cohort-based prototype is tailored for the specific situation in SDATS. It accounts for the increased mobility of the last 50 years through assessing the history of potential exposure in the two age cohorts differently. The younger SDATS cohort (20–35 y.o. in 2020) has grown up in different circumstances regarding dialect acquisition and dialect change compared to the older cohort (60–80 y.o. in 2020) [20]. With societal changes, the increasing likelihood of geographical mobility and the access to a wider variety of media, the younger cohort has had potentially more intense exposure to other dialects and to Standard German than the older cohort did at the same age. As we will show later, age is the strongest independent variable in our survey to explain language change: younger speakers tend to show more dialect change. This is also because more time has elapsed between the SDS and the younger speakers’ dialect acquisition, allowing more time for dialects to change. That is, the younger cohort has a different baseline against which their dialect might change over their lifespan [22].

The relational weights are constant across the LMI prototypes and across participants. For LMI^D^, we change this to account for the cohort-effect by implementing age-cohort-based relational weights for ‘workplace’ and ‘external residence’ in the relational weight models M3 and M4 (see details in S1 Appendix and S2 Appendix). We do so by also modelling the two age cohorts separately in M3 and M4. This enables the younger cohort to attain higher LMI values in comparison to the other LMI prototypes by allowing the linguistic effects stemming from the workplace and external residence agents to have a higher weight. Table 2 summarises the components in the four LMI prototypes and the corresponding weighting, including the cohort-based relational weights in LMI^D^.

### Evaluating LMI as a predictor of dialect change

**Fig 2:**
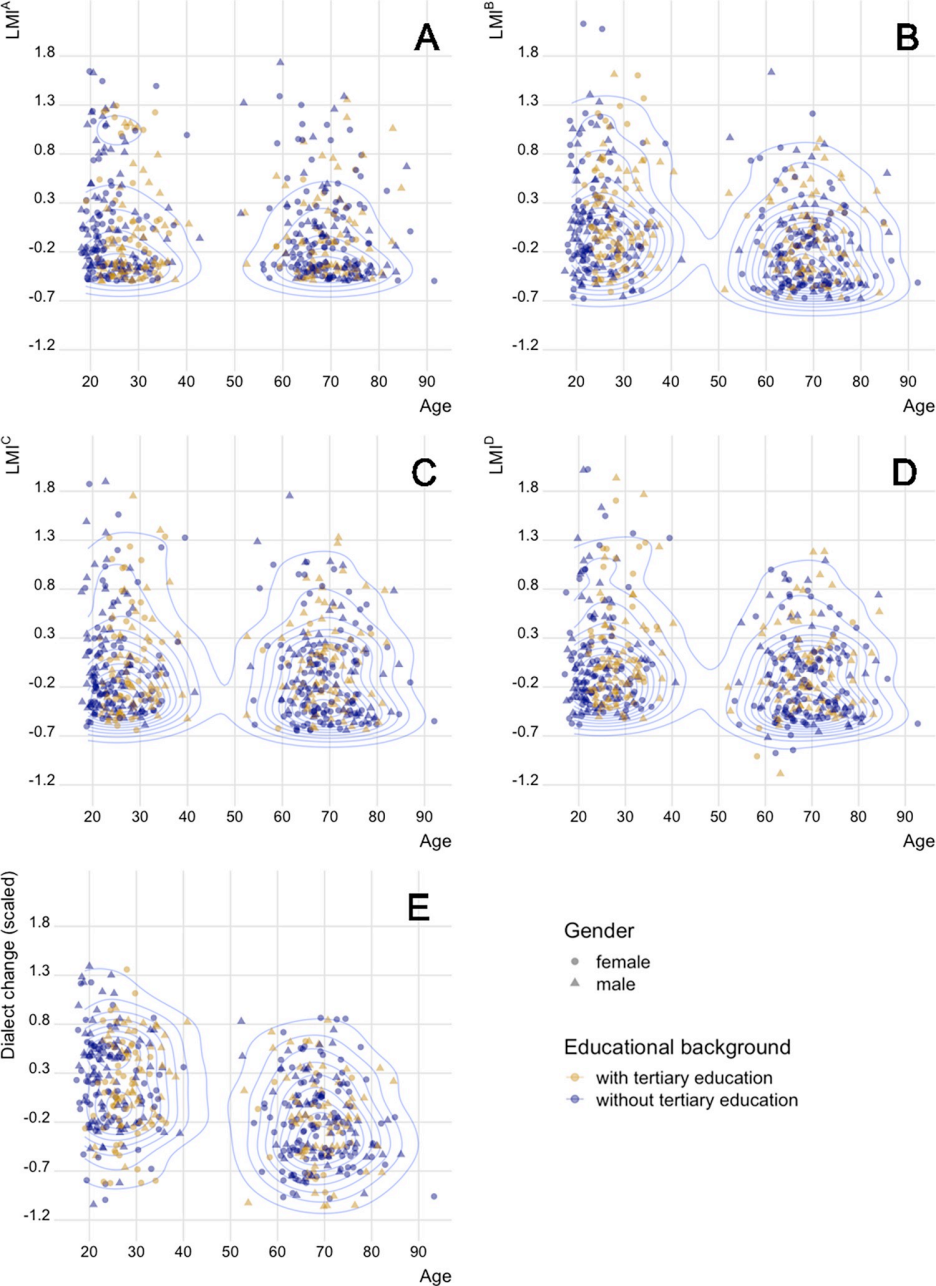
Distribution of LMI and dialect change with regards to age. In Panels (A)-(D) the distribution of standardised LMI^A^, LMI^B^, LMI^C^ and LMI^D^ values are shown (y-axes) against the age of the speakers (x-axes). Panel (E) shows the distribution of standardised dialect change rates against the age of the speakers. Each point represents a speaker (*n* = 500). Point colour represents educational level and shape represents sex. The blue concentrical lines show the density of speakers.

We will evaluate LMI by testing the performance of the four prototypes as predictors of dialect change. Fig 2A–2D shows the relationship between the four LMI prototypes with age. The shapes of the symbols represent sex and colours represent educational background. Speakers without tertiary education appear to be less mobile, while a number of younger speakers with no or ongoing higher-level education form a cluster on the left side of Fig 2A–2D. For comparison, Fig 2E presents the relationship of age, sex, educational background and the dialect change rate used for the evaluation. All panels in Fig 2 show that younger speakers have, on average, a slightly higher LMI and dialect change rate.

To test the utility of LMI as a predictor of language change, the four LMI prototypes were entered as fixed effects in logistic mixed-effects regression models. We expected the model results to confirm, while controlling for the variables based on which SDATS speakers were collected, that speakers with higher LMI have higher rates of dialect change and those with lower LMI have lower rates of dialect change. The outcome variable of the models is change rate in lexical variables, calculated based on ten items (Table 3), recorded at the 125 survey localities which were included in both SDS and in SDATS, approximately 70 years apart. The ten lexical items were chosen to represent maximal expected variation in terms of dialect change and different word frequencies based on Google Books Ngrams [53]. Lexical items, also including those investigated by Juska-Bacher [20], were chosen as the lexical level changes faster compared to other grammatical linguistic levels, according to Trudgill [54]. Table 3 shows the rates of change in the 500 speakers and the word frequency in Switzerland in 2019 based on Google Books Ngrams [52]. The greatest change occurred in the word ‘freckles’ (*Std*. *Germ*.: *‘Sommersprossen’*) followed by ‘butterfly’ (*Std*. *Germ*.: *‘Schmetterling’*), while other lexical items show a smaller, comparable rate of change. The goal of the modelling for this evaluation was neither to find the perfect model for predicting dialect change nor to explain language change at the level of individual SDATS speakers or linguistic items. Rather, the goal was to test whether LMI has validity for explaining dialect change that may not have happened within the lifetime of the speakers, but partly in the previous generations.

**Table 3:** The ten lexical items used in the evaluation study.

SDS map number	Standard German	Dialectal variants	English	Proportion of change from SDS in the 500 speakers	Frequency in Switzerland in 2019
V 179	Butter	Butter, Schmalz, Anke etc.	butter	33.2%	0.001’425%
VI 237	Schmetterling	Summervogel, Fifolter, Schmätterling etc.	butterfly	48.4%	0.000’316%
V 212	Bonbon	Zuckerli, Täfeli, Tröpsli, Bombom, Guetsch etc.	(hard) candy	29%	0.000’073%
IV 17	Wange	Wang, Wang(j)i, Backe (with fricative or non-fricative second consonant)	cheek	36.4%	0.003’38%
IV 43	Sommersprossen	Laubfläcke, Summersprosse, Merzetupf, Merzedräck etc.	freckles	72.8%	0.000’234%
IV 71	Schluckauf	Hitzgi, Gluggsi, Hixer, Hösch etc.	hiccup	41.8%	0.000’065%
V 21	Kuss	Kuss, Schmutz, Müntschi, Muntsi etc.	kiss	28%	0.004’093%
VI 179	Zwiebel	Zibele, Zwible, Bele, Böl(l)e etc.	onion	28%	0.000’55%
VI 40	Pfütze	Glunte, Gumpe, Gülle, Gudle, Glungge, Lache, Pütze etc.	puddle	42%	0.000’231%
V 139, V 140	Taschentuch	Nastuech, Naselumpe, Schnupftuech, Fazeneetli etc.	tissue (hanky)	28.2%	0.000’787%

The data on word frequency in Switzerland in 2019 comes from Google Books Ngrams, based on the Standard German version of the words.

#### Predictors of dialect change

Language change rates show differences with regard to age, sex, social class and educational background [30,55,56]. We test these empirical observations, often uncovered in sociolinguistic research on dialect change modelling, using simple linear regression models and we control for them in the mixed-effect models of the evaluation. The baselines of dialect change are different across the age cohorts, with the younger SDATS cohort’s language acquisition taking place on average 40 years later in time from the time when data for SDS was recorded. Due to this, we expect that any predictor of language change would deliver more noisy results for the younger cohort, which makes it crucial to control for age cohort in our study. Owing to the patterns of linguistic differences with similar areas and outlying dialects occurring, the baseline for dialect change is also expected to vary in space, making the spatial origin of individuals an important predictor for dialect change. However, in this evaluation, we avoided including spatial variation directly in the modelling due to the following reasons. On the one hand, we aim to provide a methodology that could be also useful for studies where speaker groups with no spatial variation are considered (e.g. urban sociolinguistics with a focus on class). In such cases accounting for spatial variation in the speaker sample is irrelevant. On the other hand, four speakers in 125 survey localities are too low a number for accounting for categorical effects in tests that assume normality. To characterise the spatial distribution of the dialect change rate and linguistic mobility, we tested the similarity of localities with regard to these values (using the Kruskal-Wallis test) and the clustering of values in space (measuring the spatial autocorrelation using Moran’s *I*). Additionally, we tested the linear effect of population size of the SDATS survey sites using federal statistical data [57].

#### Mixed-effects modelling

The mixed-effects modelling was implemented in *R* using the *lme4* package [58] (version 1.1–27.1). Each observation in the dataset represents a combination of a speaker (*n* = 500) and an item (*n* = 10), amounting to 4983 observations after removing invalid or missing answers. As fixed effects, each model includes one of the four LMI prototypes, together with the survey criteria as independent variables (Table 4). All fixed effects were z-standardised (see Eq (2)), to facilitate the interpretation of the model results [59].


$$z=\frac{x-\mu_{x}}{2\sigma_{x}}$$

**Table 4:** The variables entered in the mixed-effects models of the evaluation.

Outcome variable	
Dialect change in item	Change (1), or no change (0) compared to SDS data in the same locality
**Fixed effects**	
Linguistic Mobility Index (LMI)	Continuous variable–One of the four LMI prototypes, z-standardised (the four LMI prototypes LMI^A^, LMI^B^, LMI^C^ and LMI^D^ are used in separate models)
Age cohort	Binary variable–‘older’ > 20–35 years (-0.5); ‘younger’ < 60–80 years old (0.5)
Sex	Binary variable–female (-0.5) and male (0.5)
Highest completed education	Binary variable–with tertiary education background (-0.5) or without (0.5)
**Random effects**	
Speaker	*n* = 500
Item	*n* = 10

Binary fixed effects were contrast coded to zeroes and ones in the order of the magnitude of dialect change expected (z-standardised to -0.5 and 0.5). The standardised LMI values range between -1.62 and 2.14. Due to the presence of pseudoreplication (all speakers answered each of the ten questions), we included speaker and item as random effects in the mixed-effects models. This also controlled for the differences in the frequency with which words occur, as more frequent forms are expected to be more resistant to change [60]. Collinearity was tested through the analysis of variance inflation factors (VIFs), using the *car* package [61] (version 3.0–10.), resulting in values only slightly larger than 1; thus, collinearity problems were not expected. We also ran additional models including interaction terms across the fixed effects. For more details, consult S2 Appendix.

## Results

**Fig 3:**
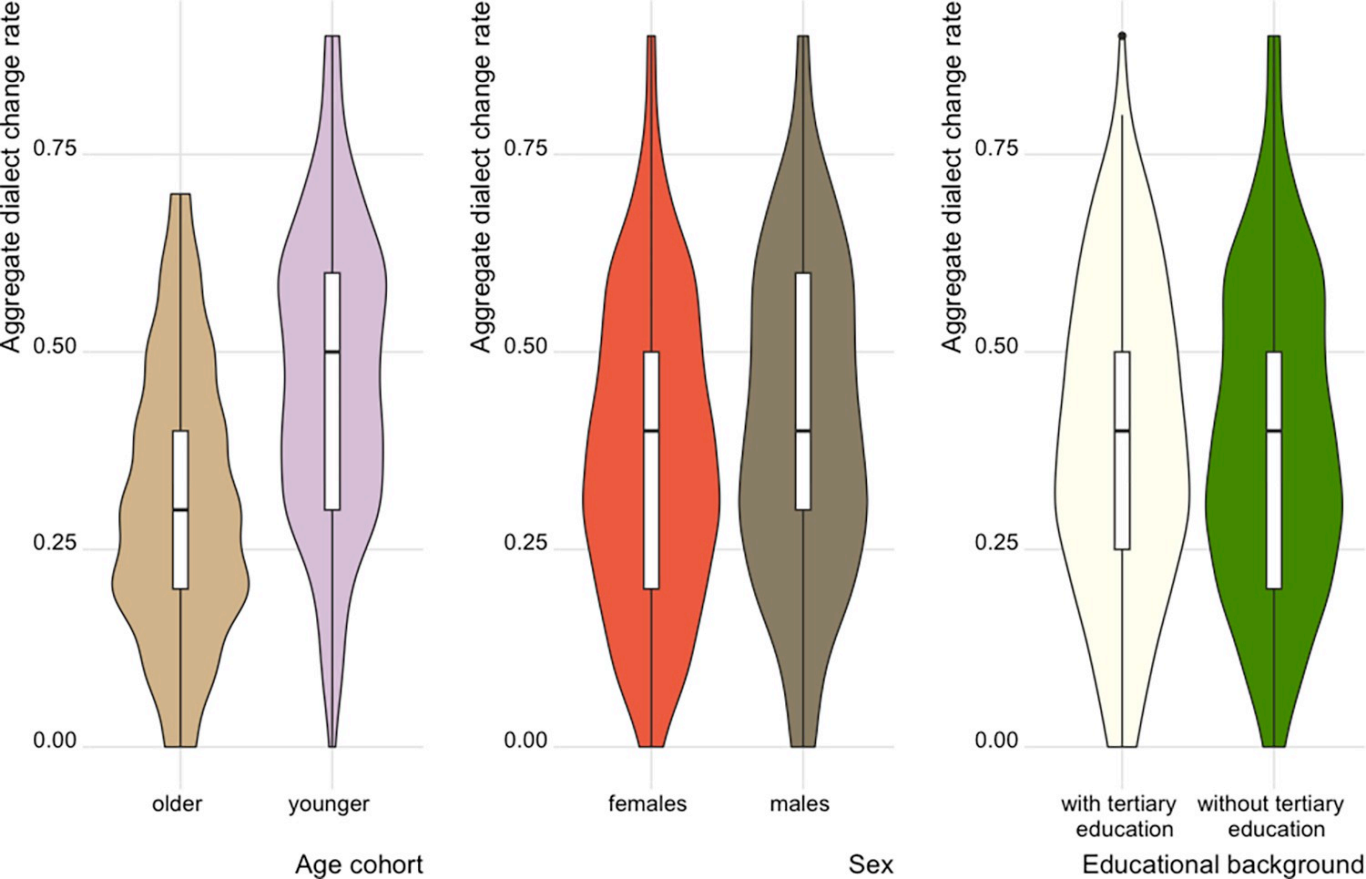
Dialect change rate for the ten lexical items by age cohort, sex and educational background.

In this section we report the outputs of the mixed-effects models described above, in order to evaluate the LMI approach as a useful heuristic for language variation and change studies. In addition, to contextualise the relation between linguistic mobility and dialect change, we characterise the spatial patterns of dialect change rate and the LMI prototypes. First, however, we briefly explore simple linear regression models of the independent variables (corresponding to the survey criteria), LMI prototypes and dialect change. Age cohort and sex are statistically significant predictors of dialect change, while education is not (see Fig 3). The younger cohort shows greater change (*μ* = 47.03%, *SD* = 18.75%) than the older cohort (*μ* = 30.8%, *SD* = 17.18%).

**Fig 4:**
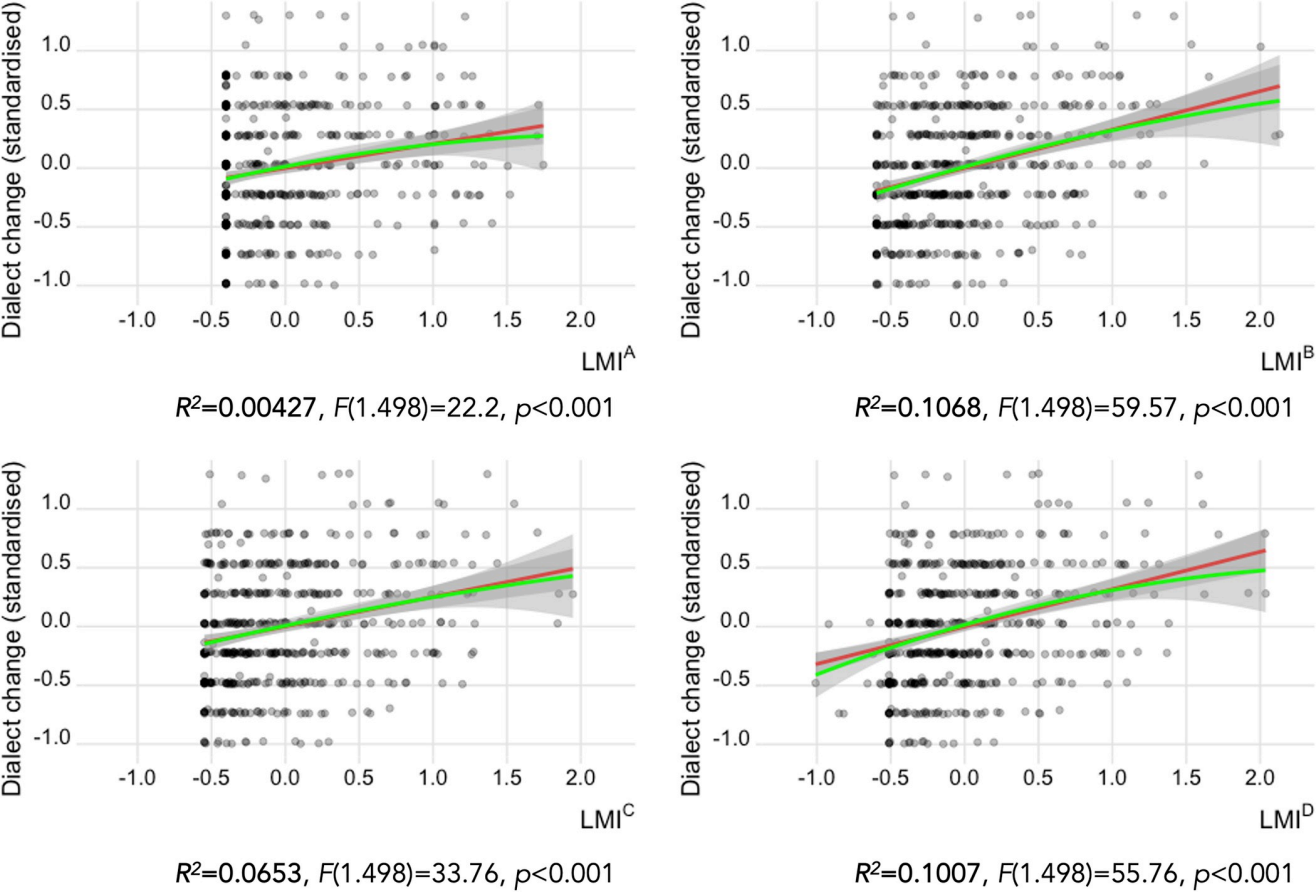
The relation between the four LMI prototypes and the dialect change rate. LMI values and dialect change rates are standardised. The panels also show the numerical results of the linear regression models. Linear (red) and second-order polynomial regression lines (green) show the major trends. The slope of the lines shows the positive correlation.

In addition, males show slightly greater change (*μ* = 40.77%, *SD* = 20.16%) than females (*μ* = 37.07%, *SD* = 19.13%). In terms of the four LMI prototypes (scaled and centred), although all four show significant predictive power, the correlation coefficients determine that LMI^B^ (the cumulative prototype, *R*^*2*^ = 0.1073) and LMI^D^ (the cohort-based prototype, *R*^*2*^ = 0.1014) are better as sole predictors of the dialect change rate than the other prototypes (Fig 4).

In the mixed-effects models, age, gender and LMI prove to be significant predictors, while educational background does not (Table 5). The LMI prototypes’ predictive power in increasing order of their *z*-values is A (minimal), C (comprehensive), B (cumulative), D (cohort-based). Their highly significant effects are coupled with low standard error (*SE*). The smallest *AIC* (Akaike Information Criterion), characterising model quality, also belongs to LMI^D^, which makes it the best model out of the four. Slope estimates and *SE* are on the log-odds scale and must be exponentiated for a more accessible interpretation. For example, two standard deviations of increase in LMI^B^, *e*^0.4395^ = 1.5519 and the corresponding *SE*, *e*^0.0784^ = 1.08155 mean a 55.19% (± 8.15%) increase in odds for dialect change.

**Table 5:** Output of the mixed-effects models involving the survey criteria of SDATS as independent variables.

**LMI^A^**	**Model quality**	*AIC*	**Random effects**	*SD*_*speaker*_	*SD*_*item*_
		6129.63		0.4809	0.6130
	**Fixed effects**	*estimate*	*SE*	*z-value*	*p*
	**LMI**	0.3486	0.0755	4.6152	<0.001
	**Age cohort**	0.7691	0.0771	9.9754	<0.001
	**Sex**	0.2022	0.0777	2.6025	0.0093
	**Education**	0.0901	0.0811	1.1105	0.2668
**LMI** ^**B**^	**Model quality**	*AIC*	**Random effects**	*SD*_*speaker*_	*SD*_*item*_
		6119.76		0.4663	0.6129
	**Fixed effects**	*estimate*	*SE*	*z-value*	*p*
	**LMI**	0.4395	0.0784	5.6041	<0.001
	**Age cohort**	0.6681	0.0789	8.4704	<0.001
	**Sex**	0.1919	0.0769	2.4942	0.0126
	**Education**	0.1374	0.0806	1.7055	0.0881
**LMI** ^**C**^	**Model quality**	*AIC*	**Random effects**	*SD*_*speaker*_	*SD*_*item*_
		6120.18		0.4672	0.613
	**Fixed effects**	*estimate*	*SE*	*z-value*	*p*
	**LMI**	0.4203	0.0755	5.5694	<0.001
	**Age cohort**	0.7596	0.0764	9.9386	<0.001
	**Sex**	0.1797	0.077	2.3325	0.0197
	**Education**	0.1228	0.0804	1.5264	0.1269
**LMI** ^**D**^	**Model quality**	*AIC*	**Random effects**	*SD*_*speaker*_	*SD*_*item*_
		6115.63		0.4602	0.6129
	**Fixed effects**	*estimate*	*SE*	*z-value*	*p*
	**LMI**	0.4602	0.077	5.9714	<0.001
	**Age cohort**	0.6934	0.0773	8.9693	<0.001
	**Sex**	0.1859	0.0766	2.4261	0.0153
	**Education**	0.1264	0.0801	1.5779	0.1146

For each model, containing one of LMI^A^, LMI^B^, LMI^C^ or LMI^D^, fixed-effect coefficients are shown in yellow, while the standard deviations of random effects are shown in green and the AIC of the model in blue.

In the case of each LMI prototype, the predictive power of age cohort shows a decisive effect. They are significant, with estimates larger than LMI’s estimates and a *SE* similar to LMI’s *SE* values in each case. Age cohort reaches the highest estimate and z-value in the case of LMI^A^ and LMI^C^ where, in turn, LMI estimates are lower compared to LMI^B^ and LMI^D^. The effects of sex are significant and show similar estimates across the prototypes. Relative to the estimates of age cohort and LMI, *SE* values of sex are higher. Educational background does not have a significant effect for any LMI prototype (although it is almost significant in LMI^B^), and its *SE* values are high. Comparing the different predictors, LMI emerges as a better predictor than sex and education in every case. The effect of age cohort dominates over the other variables, while the effect of sex is larger than that of education.

A heuristic explanation of the importance of the random effects is provided by comparing their standard deviation (*SD*) to the estimate of a fixed effect. If the *SD* of the random effects is larger than the LMI prototypes’ estimates, then the speaker and item effects are larger than the effect of LMI. This means that dialect change depends more on the linguistic items tested and the differences across speakers than the effect of linguistic mobility.

When we consider the interaction between age cohorts and LMI prototype in the models, we find a significant effect only for LMI^A^ (Table 6). This fact and the negative estimate of the interaction term indicate that the difference in LMI’s effect is smaller than expected within the younger cohort compared to the older cohort. The lack of a significant interaction between these variables in the other models tells us that the effect of LMI does not depend on age cohort and therefore LMI is a predictor of similar worth in both age cohorts. There is also no significant interaction between age cohorts and sex in the context of any of the prototypes either, which means that the effect of sex is very similar within the two age cohorts and vice versa. Submodel ranking, carried out using the *MuMIn* package [62] (version 1.43.17.) shows all fixed effects as significant contributors to the model quality. For details, please consult S2 **Appendix.**

**Table 6:** Addition of an interaction term between age cohort and the minimal LMI prototype.

**LMI** ^**A**^	**Model quality**	*AIC*	**Random effects**	*SD*_*speaker*_	*SD*_*item*_
		6125.35		0.4710	0.6132
	**Fixed effects**	*estimate*	*SE*	*z-value*	*p*
	**LMI**	0.3754	0.0756	4.9662	<0.001
	**Age cohort**	0.7716	0.0766	10.0723	<0.001
	**Sex**	0.1998	0.0772	2.5886	0.0096
	**Education**	0.0921	0.0806	1.1432	0.2529
	LMI*Age cohort	-0.3805	0.1509	-2.5206	0.0117

To contextualise linguistic mobility and dialect change with spatial processes, we provide our insights regarding the spatial distribution of dialect change and LMI. Although a Kruskal-Wallis test (Table 7) shows significant differences across the 125 localities with regard to the dialect change rate based on the ten lexical variables (*χ*^*2*^ = 266.82, *df* = 124, *p* < 0.001), these also stem from the different regional baselines regarding dialect change, i.e., there is also spatial variation in the lexical items in the original data from SDS, e.g. changes still ongoing in some parts of the dialect area have already finished earlier in other parts. Regarding LMI prototypes, we also find significant local differences using the Kruskal-Wallis test, except in the case of LMI^A^.

**Table 7:** Spatial characteristics of the dialect change rate and the LMI prototypes, using the Kruskal-Wallis test and measuring the spatial autocorrelation using Moran’s I.

	Dependence of distribution (Kruskal-Wallis test)			Spatial autocorrelation (Moran’s *I*)			
		*χ* ^*2*^	*df*					*p*	*I*	*I*_*expected*_	*SD*	*p*
**Dialect change rate**	266.82	124	< 0.001	-0.0223	-0.002	0.0012	< 0.001
**(10 variables)**							
**Minimal (LMI** ^**A**^ **)**	141.17	124	0.1388	-0.004	-0.002	0.0012	0.0946
**Cumulative (LMI** ^**B**^ **)**	155.14	124	0.0305	-0.005	-0.002	0.0012	0.0013
**Comprehensive (LMI** ^**C**^ **)**	152.06	124	0.0442	-0.0053	-0.002	0.0012	0.0068
**Cohort-based (LMI** ^**D**^ **)**	153.02	124	0.0394	-0.0055	-0.002	0.0012	0.0034

**Fig 5:**
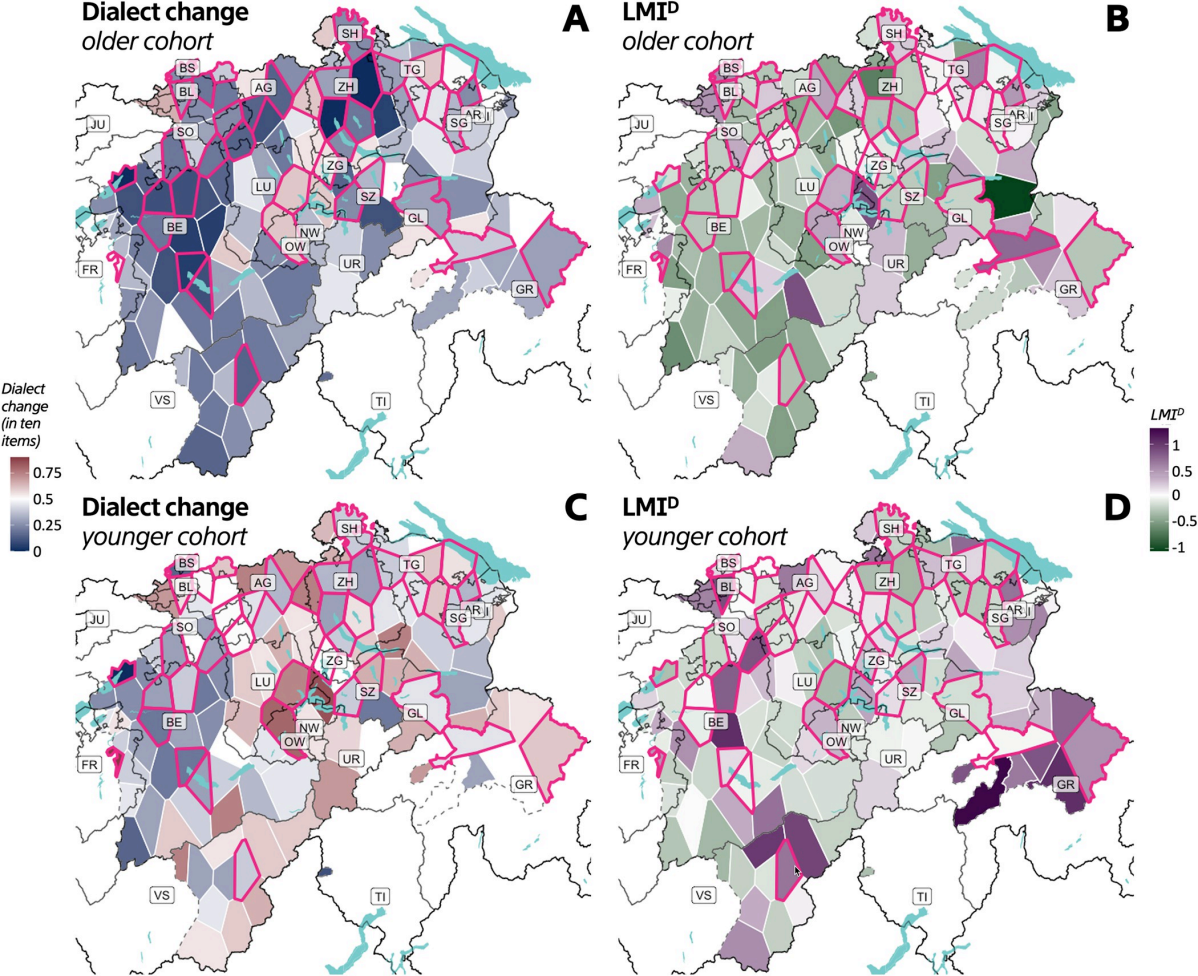
Spatial patterns of dialect change rates and LMI^D^. Panels (A) and (C): The average rates of dialect change are shown (on a scale of 0 to 1) in the polygons representing SDATS localities. Panels (B) and (D): The standardised LMI^D^ values of each SDATS locality are shown with a different colour scale. The darker blue and green colours (respectively) mean a lower value, while the darker red and purple colours mean higher values. In each map, those SDATS survey localities qualifying for the city rank in Switzerland (10000< inhabitants) are highlighted with magenta edges.

Due to the aforementioned, potentially different, regional baselines of dialect change and the regional variation present in Swiss German dialects, we will address the expected correspondence of spatial patterns in the dialect change trends and linguistic mobility. In the polygons of the 125 SDATS survey sites, Fig 5A and 5C chart the dialect change rates, while Fig 5B and 5D show the LMI^D^ values, for the two age cohorts. Studies have shown that urban areas are associated with greater dialect change [63]. SDATS localities that had more than 10,000 inhabitants in 2018, qualifying for the ‘city’ rank in Switzerland, are shown with magenta edges. It is visible from the maps in Fig 5A and 5C, however, that urban areas, especially in the cantons of Bern (BE) and Zurich (ZH), show the least dialect change in both age cohorts. Population of the survey localities shows a significant negative linear correlation with language change (*R*^*2*^ = 0.032, *F*(1,498) = 16.4824, *p* < 0.001), meaning that the higher the population, the less language change can be expected. Dialect change in the non-urban areas below 10,000 inhabitants (*μ* = 40.06%, *SD* = 19.12%) is almost significantly higher (*t* = 1.9069, *df* = 498, *p* = 0.057) than in the urban areas (*μ* = 36.47%, *SD* = 20.78%). As the largest urban localities change remarkably less than rural areas do, and urbanity is a fluid characteristic, this definition of ‘urban’ seems to influence the result of the test. Also, contrary to naive expectations about dialect preservation, rural and mountainous areas, especially in the centre and south-east (e.g. cantons of Lucerne, Nidwalden, Obwalden, Uri and Valais–LU, NW, OW, UR, VS, respectively), show the most dialect change in the younger cohort. Moran’s *I* analysis, however, shows significant negative spatial autocorrelation in dialect change rate (*I* = -0.0223, *SD* = 0.0012, *p* < 0.001). This means that there is a higher likelihood of finding different dialect change rates in nearby localities rather than similar ones. In other words, dialect change does not cluster in space in our sample. The reason for this may be the granularity or the survey site network. As the localities are often far apart and they are part of distinct spatial clusters characterised by differences regarding urbanity, commute network and identities, we may not assign a clear explanation to the spatial patterns of dialect change rate.

Geographic patterns of LMI^D^ also show a negative spatial autocorrelation (*I* = -0.0055, *SD* = 0.0012, *p* < 0.0034) with little visual similarity across the age cohorts (Fig 5B and 5D) except in rural parts of BE and LU, and the generally suburban region of ZH. Regarding the spatial autocorrelation of the other LMI prototypes, Moran’s I values are also negative, i.e., mobile or non-mobile speakers do not cluster in space. LMI, thus, cannot be safely interpreted as a function of geography in our sample. Although trends of slight correspondence were seen in Fig 4, visual inspection of Fig 5 does not reveal spatial correspondence between LMI^D^ and dialect change rates except for parts of BE and ZH being less mobile and showing less change. A table containing all 125 SDATS localities with their average dialect change rates and average LMI values per age cohort is included in S2 **Appendix (Section 5)**.

## Discussion

In this section we discuss LMI as a practical approach for researchers analysing language variation and change by reflecting on its composition and evaluation in our study. Following this, we present the limitations of LMI and its implementation, and make further recommendations about ways linguistic studies could adopt the LMI approach.

### LMI as a useful approach for language variation and change studies

With (spatial) mobility growing in the 20^th^ and 21^st^ century, any linguistic survey addressing with spatial variation must consider the mobility of its participants. Previous studies, exemplified by the Regionality Index (RI) [1,16–18], estimated the ‘localness’ of a dialect by measuring the exposure of the participants to a reference locality or a variety. Our proposed Linguistic Mobility Index (LMI), while not claiming universal superiority, quantifies exposure to variation outside a reference, making it a viable alternative under suitable data conditions. We recommend LMI as a quantitative indicator of exposure for purposes similar to that of RI, acknowledging the nuanced scenarios where each approach may excel.

Mobility has been shown for several languages to influence language change, including in theoretical work (e.g. [3,9,11]) and in studies on different linguistic levels [1,2,5,6,8]. In terms of the speakers’ biographical data used for establishing its effects, however, these studies were less complex. Thus, we suggest that a more extensive quantitative heuristic aggregated from biographical data is more appropriate for the task of elucidating the effect of geographical and linguistic mobility on language change. This paper has shown the wider applicability of LMI by evaluating four prototypes that simulate differences in the availability of biographical data and test different theoretical considerations. In addition, the two age cohorts in our dataset has allowed us to test different baselines of dialect change. Notably, implementing the LMI approach is flexible as the steps involved (i.e. calculation of linguistic distances, exposure weights, and relational weights) can be tailored to the researchers’ needs and the available data.

### Discussion of the findings specific to the application to SDATS data

This study was conducted by modelling dialect change in Swiss German using the LMI prototypes and the SDATS survey criteria (age, sex, educational background). Our evaluation tested whether the patterns of linguistic mobility characterising speakers that acquired their dialect under different circumstances (due to age, education, residence etc.) are suitable predictors of language change patterns that might not be the direct consequences of the speakers’ dialect acquisition. These complex patterns of language change make the evaluation of the predictors difficult in terms of establishing causality. Spatial diversity and covert prestige make Swiss German dialects an ideal environment for studying mobility-induced change, as opposed to e.g. media- or prestige-induced change. This is because there is a great deal of variety in a relatively small area, with people that use their own variety almost anywhere they go, thus diverse contact situations occur constantly.

In the Swiss German context, the results of the mixed-effects models can be interpreted in line with our expectations: speakers with higher linguistic mobility (LMI) show greater dialect change (Table 5). This corresponds to previous observations which show that being sedentary is a countereffect for dialect change in the Swiss German context (e.g. [20,41]). Each LMI prototype proved to be a significant predictor when controlling for the survey criteria acting as independent variables (Table 5). In addition to the mixed-effects model, we also showed the role of age and sex on dialect change in bivariate tests (visually in Fig 3). Linear regression models relating the LMI prototypes to language change (Fig 4) showed the cumulative LMI^B^ and cohort-based LMI^D^ as better sole predictors over the other prototypes. Regarding LMI, differences across the four prototypes also reflect the role of age (Fig 2A–2D): no clear age-related pattern is present for LMI^A^, but to a certain degree other prototypes show higher mobility for the younger cohort. The fact that younger speakers show more dialect change, which we expect LMI to predict, indicates that it is crucial to consider age when implementing LMI (for SDATS and elsewhere too).

Amongst the LMI prototypes, LMI^D^ holds the top position (based on *AIC* and *z*-values) as the best heuristic. We associate this success with LMI^D^ addressing the differences of biographical data availability between the two age cohorts, an operation that makes it more complex than LMI^C^. Its top position is not so clear, however, which means that the fixed effects regulate the explanatory power of the LMI prototypes. That is, the differences in the composition of the LMI prototypes mattered less for their explanatory power when we controlled for age, sex, and educational background. The cumulative prototype (LMI^B^), not involving exposure weights, performed similarly to LMI^D^. From this, we can infer that in our study, the critical aspect was not the intricate details of the LMI implementation but rather the accuracy of capturing the mobility-related factors that influence dialect change. In comparison to other predictors, LMI has a stronger effect on dialect change than sex and educational background do (i.e. an increase of two standard deviations in the LMI values increases the odds of dialect change more than binary switches in sex and educational background do).

The high speaker standard deviation (*SD*_*speaker*_) value compared to the estimates means that random effects capture more variance in dialect change than LMI, sex or educational background do. Therefore, between-speaker variation, not accounted for by the LMI prototypes, is still very important for dialect change. This means that parts of the speakers’ linguistic biographies that could not be included in LMI make a difference. These may include contacts not present in the biographical data elicited by the questionnaire, personality, dialect attitudes etc. Besides, local baselines of dialect change also involved in personal variation (random speaker effect) still contain crucial effects.

Corroborated by the strong age effect on dialect change (Fig 3), age cohorts maintain the highest estimate in each model, demonstrating that the manner of including an age effect in LMI is crucial when explaining language change. The significance of the interaction of age and LMI in the case of the minimal prototype (Table 6) means that LMI^A^ is a weaker predictor in terms of the age effect. The negative slope estimate of the interaction of age with LMI^A^ tells us that the difference in LMI’s effect is smaller than expected within the younger cohort than in the older cohort.

Sex is a significant predictor in the case of each LMI prototype. When keeping age cohort, educational background and linguistic mobility constant, more change is expected in men’s dialects compared to women’s. Estimates of sex are similar across the four models, which means that its effect is not influenced by the composition of the LMI prototypes. As a matter of fact, the LMI prototypes are constructed the same way for both sexes.

Educational background is not significant in any of the models, with a large standard error (*SE*) which shows its lower value as a predictor. The reason for this may partly be due to the suboptimal categorisation of various occurring educational backgrounds into two groups (also recall that the dataset includes many young speakers attending higher education who are not yet eligible for the ‘with tertiary education’ label), as well as the fact that social stratification does not highly influence dialectal differences in Switzerland.

The spatial analysis showed that LMI values and dialect change rate do not cluster or correspond to each other in space. This lack of relationship indicates that the trends within and correspondence between LMI and dialect change are independent from their spatial variation. In other words, low mobility may not necessarily be the cause of little dialect change. Similarly, change may not occur even in very mobile speakers. Because of the relatively low number of linguistic items tested and since the SDATS survey criteria prescribed participants to be more sedentary than average, we can only speculate on a cause. Perhaps the pattern is an artefact of the selected lexical variables. The levelling of Swiss German variation [20,41] can also play a role in this pattern, suggesting that locally there may no longer be a more prestigious variant for people to adopt.

Urban lifestyle is often associated with higher general mobility, but in our sample linguistic mobility does not correspond to this preconception (Fig 5B and 5D, magenta-edged polygons). Also contrary to this preconception, the population of SDATS survey localities shows a slightly negative correlation with dialect change rate across the ten lexical items, which means that there is less dialect change in localities with a larger population. Correspondingly, rural areas in Fig 5A and 5C, represented by white polygon edges, show more change (white to redder hues) than cities (magenta edges), especially in the younger age cohorts. Considering ongoing dialect levelling in Switzerland, the rural population seems to align with the local urban varieties [64], although given that rural dialects often define their own identities by rejecting urban variants, this pattern might be an artefact of the linguistic sample. Intuitively, however, the spatial pattern of dialect change corresponds to the increasing geographical mobility, and thus it is more difficult to unravel based on our restricted sample of linguistic items. In order to find true regional and geographic effects in dialect change, a more detailed dialectometric study would be necessary, investigating a larger set of linguistic items.

### Limitations

Beyond the standard limitations and potential drawbacks associated with dialect surveys and their data elicitation practices, such as (socio)linguistic interviews and (unsupervised) collection of biographical data, the following limitations may also hinder the implementation of the LMI approach.

Owing to the limitations of the unsupervised collection of biographical data, inconsistent answers may always occur and some data may not be sufficient for inferences to be drawn on speakers’ mobilities. For example, issues with the automated geolocation arise due to the inconsistency or ambiguity of localities indicated by the speakers. The COVID-19 pandemic ongoing throughout the data collection affected several SDATS questionnaire items directed at short-term mobility and social networks. Effects of short-term mobility were tested in two unpublished master theses in relation to dialect change [65,66], but no predictive effect was found. Additionally, questionnaire items regarding *current* linguistic connections to peers do not necessarily characterise long-term contact. Therefore, this study could not consider such questionnaire items.

Although the SDATS questionnaire serves a wide array of research purposes, the concept of the present study had not directly affected its composition. Specific problems with the SDATS questionnaire items impact the composition of LMI, as the collection of biographical data varied by kinds of agents, i.e. different sorts of information were elicited on each kind of agent. For example, information is available about the speakers’ parents, their current most significant peer (the partner), place of education (if currently attending higher education), and workplace. It is also possible to make inferences on the reference locality from different kinds of aggregate information, which (indirectly) indicate childhood and adolescent peer effects. There is, however, no information about the duration of contact between the speaker and the people elicited from their social networks, such as their flatmates and their closest personal and work-related peers or the size of their social circles. Regarding residence outside the reference locality, information is available about locations and durations, but not the age at which the speaker lived there. As a result, it was not possible to evaluate LMI by testing its predictive performance against mobility predictors based on the number of years spent away from the reference locality (cf. [6,8]). Our study is also limited by the disparity in available information on adolescence and other potential LMI components between the younger and older cohorts. For instance, information is missing on pensioners’ last workplace, as is the last place of education for those not attending any education at the time of the survey. Due to their age, the older cohort’s baseline regarding dialect change is closer to the SDS, whereas for the younger cohort there is a higher probability that the dialectal forms learnt have already undergone change in comparison to the SDS baseline in previous generations. This makes the older cohort more optimal subjects in the present study for assessing whether linguistic mobility impacts dialect change directly, as dialect change may have happened during their lives that was actually caused by some of the agents or environments included in LMI.

Given that SDS data recorded from NORMs and NORFs in the 1950s and ‘60s was used for calculating linguistic distance, estimates of exposure may be biased. In our study, the linguistic distance values based on this 70-year-old data are somewhat conservative, as linguistic distance values on the well-connected Swiss Plateau (Ger.: *Schweizer Mittelland*) have probably decreased more since the 1950s than they did in mountainous, more isolated areas.

### Recommendations for applying the LMI approach in other studies

In this section, we summarise a few issues to consider in order to facilitate the adoption of LMI in other studies, organised according to the steps outlined for the implementation of the LMI approach. Following this, we provide a few scenarios where we see the implementation of LMI warranted.

Most importantly, a researcher must fine-tune the approach for their own goals. There is no single best LMI implementation. Every composition will have a certain degree of subjectivity, and LMI’s predictive power will depend on the speakers in the dataset and on the linguistic settings of the research. Prior to implementing LMI, crucial influences on the outcome variable in the specific study should also be determined. Such agents may include, in addition to those tested in the present study (age, sex, educational background, spatial variation), the role of the standard language, the strength of local identity (e.g. [67]) and the power relationship between urban and rural dialects [64,68], which might boost or counter the effects of mobility. Moreover, an inclination or reluctance towards dialect change may be associated with personality, for example, openness, extraversion, or pride in one’s dialect [43]. In terms of language change studies, age and the time elapsed between points of comparison may be crucial as change accumulates over time, meaning that younger speakers would typically show more dialect change. This is exemplified by the emergence of the age-cohort-based LMI^D^ as the best predictor in our study. In order to determine which questionnaire items to include in the composition of LMI, we advise testing the items as single predictors of the phenomenon investigated and also as aggregates, similarly to the model we used for establishing relational weights. In this manner, it is possible detect those items that affect the outcome variable the most.

Determining the linguistic distance between the speaker and the dialectal location of an agent is a possible way to estimate potential linguistic effects despite the fact that the exact effects of the agent are unknown. Given the considerable spatial autocorrelation of linguistic variation [38], alternative assessments may be considered in cases where the computation of linguistic distances is not feasible. Such alternatives may involve employing proxies such as geographical distance or travel time (e.g. [5,49,69]). Regarding the imperfect linear correlation (which is in several cases logarithmic) between growing linguistic and geographic distances, however, caution should be exercised.

Quantifying the actual linguistic impact of people encountered on a speaker is close to impossible. By implementing average values such as the flat relational weights for types of agents and for the linguistic distance to Standard German, a certain dialect variety or place can be assigned to agents and environments, and available data about the relationship to places (through specific people encountered) can be quantitatively used. Linguistic distances between pairs of localities are also flat rates in our implementation, but other studies may choose other estimations of linguistic differences. Nevertheless, any composition should be a valid, comparable measure for all speakers in the sample. Another factor one might also consider is prestige. It would be possible to collect linguistic variables where the variation is dictated by prestige or one could devise asymmetric linguistic distances or asymmetric weights between localities whose dialects are less or more prestigious. In a related manner, one must consider the role of local identities and prestige of dialectal communication in the language or region of concern, as dialectal forms used may vary depending on the social situation.

Exposure weights are also implemented as flat rates in our study. In other studies, they will be specific to available questionnaire items about speaker biography, research questions, language and culture, beside practical and theoretical considerations. In our study, relational weights are modelled based on influential agents, but other researchers may estimate them through their own assessment. Our LMI prototypes confirm several significant effects, demonstrating their effectiveness at capturing long-term exposure to dialect variation. It means that the global meaningfulness of LMI may not depend heavily on the minor details of its construction but whether it successfully captures long-term exposure to dialectal variation from (often noisy) data. Using a double-weighting system similar to our study is, however, not the only way to operationalise individuals’ relationships and exposure to influencing agents and environments. The determination of relational weights, other than defining them as flat rates, is especially challenging given the vast between-speaker variation and it would require the elicitation of very specific biographical data.

LMI can also be implemented with minimal biographical data collected. Naturally, the implementation of LMI has greater explanatory power when a larger number of agents is considered that are especially important in shaping one’s language, such as information on peers before and around adolescence [22], or a comprehensive collection of sociodemographic information and biographical data that allows researchers to assess the duration, intensity and chronological order of linguistic effects on an individual. Nevertheless, the significant performance of LMI^A^, despite its composition containing a limited number of agents, indicates that implementing LMI is possible in a wide range of linguistic studies. LMI^A^’s different effects in the two age cohorts, however, raise caution for implementations using limited biographical data. Through the success of the cumulative strategy that did not use exposure weights (LMI^B^), implementing LMI by adding up influential agents in the biographical data may also be a possibility. In certain cases, especially for studies with a smaller number of speakers, setting LMI components manually may also be reasonable, e.g. based on qualitative or anecdotal information available, but comparability across speakers should be maintained.

Beyond similar language change studies to the one presented in this paper, the suitability of LMI could also be tested in lifespan studies (e.g. [70]), investigating real-time effects of mobility and exposure to variation, rather than apparent-time effects through points in time recorded in different surveys.

Sociolinguistic studies found that women [28], people with a higher level of education [29], people displaying extraversion [52], or social centrality [35] show more proneness to language change. LMI may also be useful for contributing to researching those sociodemographic characteristics that increase the likelihood of people adopting innovations. Further studies, thus, might find that the linguistically mobile (i.e. those with high LMI, thus a higher exposure to varied dialects or languages, and in the case of most languages, exposure to the standard) are more likely to be linguistic innovators, while the linguistically non-mobile are the laggards more resistant to change.

Dialectological research mostly assigns their participants to a single survey locality. The LMI approach could be a step towards the goal of addressing the phenomenon of mobility causing dialect change. This could also be achieved in a multifaceted localization of speakers, by separately measuring the degree to which an individual is exposed to different localities and varieties. Linguistic mobility could be implemented as a spatially directed predictor based on the geographic mobility network of the population, supporting Trudgill’s theory of linguistic gravity [9]. For example, (historic) commuting and relocation patterns could be used to quantitatively investigate whether the individual patterns of linguistic change (e.g. levelling of dialects) correspond to their own mobility patterns, or whether language change patterns of an individual conform to the mobility patterns of the population. In addition, the term ‘mobility’ may represent the linguistic effects of exposure not only to other regions but other, social factors (e.g. social classes, other social groups or networks) as well.

Parallel to society becoming more mobile, the numbers of classic NORMs and NORFs are dwindling and the potential of language change reaches the most outlying villages, it seems imperative for linguists to consider the mobility of speakers in their samples (cf. [3]). We estimated speakers’ potential exposure to linguistic variation and successfully modelled its effects on dialect change. This allows us to endorse the modified implementation of LMI to other researchers which would contribute to the methodological adaption of linguistic surveys to the challenges of a changing and mobile world.

## Supporting information

S1 Appendix Constructing LMI–R Markdown report in html format. The report describes with code the construction of LMI components from data of the SDATS survey questionnaire.
(HTML)

S2 Appendix Mixed-effect models–R Markdown report in html format. The report presents the composition of the four LMI prototypes evaluated in this article, conducting the statistical tests and modelling reported here including their summary results. The reproducible code for both reports can be accessed at https://osf.io/hfbpk/.
(HTML)
