# Supplementary material for: Effects of mobility on dialect change: Introducing the linguistic mobility index
Source: PLoS One. 2024 Apr 16;19(4):e0300735. doi: 10.1371/journal.pone.0300735 (PMC11020475; doi:10.1371/journal.pone.0300735)
Supplement: S1 Appendix — The report describes with code the construction of LMI components from data of the SDATS survey questionnaire. (HTML) [file pone.0300735.s001.html]

Effects of mobility on dialect change: Introducing the Linguistic Mobility Index, Part 1 - Setting up the LMI


Code 

- Show All Code
- Hide All Code

# Effects of mobility on dialect change: Introducing the Linguistic Mobility Index, Part 1 - Setting up the LMI

#### Péter Jeszenszky, Carina Steiner, Adrian Leemann

#### 2024-03-01

# 1 Preface

This Supplementary Material describes the construction of
**Linguistic Mobility Index** and its components using data
from the SDATS survey (Leemann et al., 2020) questionnaire, with code.
Click on the `Code` button in the top right to show the code.
Some code chunks are still hidden, but they can be followed in the .Rmd
file of the same name.

In **Supplementary Material Part 2**, four LMI
prototypes are composed and they are evaluated by testing them in
mixed-effect models with dialect change rate as the outcome
variable.

For setting up the LMI components we calculate linguistic distances
between speaker and the long-term linguistically influential effect
(*“agent”*), and weight them with exposure weights which depend
on the questionnaire item values of speakers regarding their exposure to
the agent. Then, relational weights are calculated which are uniform
across speakers and denote a generally assumed average influence of each
agent considered.

The linguistic distances are calculated between the reference
locality of the speaker and a locality assigned to each agent. For this
study, the following agents are used:

- mother’s origin,
- father’s origin,
- partner’s origin,
- place of work,
- place of ongoing education (if exists), and
- additional places of residence through one’s life (external
  residences).

*Agents and connecting quetionnaire items
used in the study are highlighted in black*

Importantly, unlike other (sociolinguistic) studies that involve
social circles and closest relationships in studies, we did not include
friends’ circles in our analysis because the information recorded in
these regards was only related to a few people with which the speakers
currently had the most intense relationships whereas the other agents
involved would signal more reliable information about longer term
effects.

For this research, we use pre-calculated linguistic distances which
was done for a previous paper, defining the reference localities of the
SDATS dialect survey (Jeszenszky, Steiner and Leemann, 2021). The
linguistic distances needed are searched from a data base in this
supplementary material. Details on the calculation of the linguistic
distance are in the manuscript and in Jeszenszky, Steiner and Leemann,
2021.

# 2 Calculation of the LMI components

First we import the SDATS sample speakers’ (*n*=500)
biographic dataset, containing the information based on which agents and
environments used in the LMI prototypes are composed (it is a subset of
the more than 300 questionnaire items elicited in an online
questionnaire from the speakers, after the dialect interviews, in an
unsupervised manner.)

Then, we set up the data frame which we can use for subsetting for
the calculations of various LMI components.

```
LMI_df <- read.csv("metadata_SpeakerSample.csv", header = T, stringsAsFactors = F)
```

Coloumn names’ meaning:

- **UID** - Unique IDentifier for each SDATS
  participant
- **Age** - Actual age given in the SDATS
  questionnaire
- **Sex** - biological sex
- **site\_code** - identifier of the SDATS reference
  locality
- **LAT** - geographical latitude of the center of the
  Voronoi-polygon assigned to the reference locality
- **LONG** - geographical longitude of the center of
  the Voronoi-polygon assigned to the reference locality
- **SDS\_CODE\_standard** - identifier of the reference
  locaility in the SDS survey (Hotzenköcherle et al. 1962 - 2003). SDATS
  survey localities are a subset of these
- **SDS\_canton** - canton (Swiss administrative unit)
  of the reference locaility during the SDS survey
- **SDATS\_canton** - canton (Swiss administrative
  unit) of the reference locaility during the SDATS survey
- **Age\_cohort** - age cohort, young (18-35 years old)
  or old (60-85 years old)
- **Edu\_bkgr\_4cat** - highest finished education
  categorised in four categories
- **Edu\_bkgr\_2cat** - highest finished education
  categorised in two categories
- **Origin\_loc\_father** - father’s locality of
  origin
- **Origin\_canton\_father** - canton of father’s
  locality of origin
- **Origin\_loc\_mother** - mother’s locality of
  origin
- **Origin\_canton\_mother** - canton of mother’s
  locality of origin
- **Housing\_arrangements** - who does the speaker live
  with (alone, with parents (with or without siblings present), with
  partner or with flatmates )
- **PrivatePerson(1-3)\_relationship** - speaker’s
  relationship to the three private contacts listed as those most
  frequently contacted
- **PrivatePerson(1-3)\_Origin\_loc** - where this
  private contact comes from
- **PrivatePerson(1-3)\_Origin\_canton** - from which
  canton this private contact comes from
- **Origin\_Flatmate1** - where does the first listed
  flatmate of the speaker come from (needed for identifying where the
  speaker’s partner comes from)
- **AdditResidence(1-10)\_duration** - how long has the
  speaker lived in a place outside their reference locality (10 places
  could be listed)
- **AdditResidence(1-10)\_name** - names of these
  places
- **Occupational\_situation** - does the speaker work,
  study or are they pensioners
- **OnlyWorkplace\_loc** - locality of workplace (if
  only one workplace is listed)
- **OnlyWorkplace\_canton** - canton of workplace (if
  only one workplace is listed)
- **Workplace1\_loc** - locality of workplace (if more
  than one workplace is listed)
- **Workplace1\_canton** - canton of workplace (if more
  than one workplace is listed)
- **Current\_Workplace\_duration** - how long has the
  speaker been working in the current workplace
- **Language\_work\_CHD** - Proportion of Swiss German
  (dialect) used at work
- **Language\_work\_HD** - Proportion of Standard German
  (incl. Swiss Standard German) used at work
- **Edu\_loc** - location of the educational institute
  the speaker is attending
- **Language\_edu\_CHD** - Proportion of Swiss German
  (dialect) used at education
- **Language\_edu\_HD** - Proportion of Standard German
  (incl. Swiss Standard German) used at education

## 2.1 Set up the final data frame into which LMI components will be saved

This data frame will contain the *linguistic distances* and
*exposure weights* that will be used to set up the LMI prototypes
in Part 2. More on what exposure weights mean in the manuscript.

```
LMI_dfEnd <- LMI_df %>%
  dplyr::select(UID,SDATS_canton,Age_cohort,Sex,site_code,SDS_CODE_standard, LAT,LONG,
         Edu_bkgr_2cat, Edu_bkgr_4cat, Occupational_situation)
```

## 2.2 Import linguistic distances too

The tables contains linguistic distances calculated between the
survey localities (*n*=565) used in the SDS atlas (Sprachatlas
der deutschen Schweiz, Hotzenköcherle & Baumgartner, 1962-2003 –
data recorded mostly in the 1950’s). The linguistic distance
calculations are based on Yves Scherrer (2021) and colleagues’
digitisation of 289 variables in the SDS.

The imported table contains these linguistic distances for every pair
of SDS survey localities. The SDATS reference localities are a subset of
the SDS survey localities (details in Jeszenszky, Steiner & Leemann,
2021).

```
dyadicLingDists <- read.csv("SDS_dyadicLingDists.csv", header = T, stringsAsFactors = F)
```

Next, we calculate the components of LMI agent by agent, saving them
into the final data frame `LMI_dfEnd`.

By a component of LMI we mean the agents included in LMI (i.e. the
index components representing the linguistic exposure to the
**father, mother, partner, last workplace, place of education and
external residences** (the latter as an aggregate)),
i.e. linguistic distances weighted by the exposure weights calculated
below.

Below we calculate an *exposure weight* that is associated
with each agent. Doing the following calculations would also be possible
in a shorter manner but we chose more explicit coding.

# 3 Calculating linguistic distances and exposure weights for each agent

We aim to estimate the intensity of the potential exposure to
different places through the agents based on information in the SDATS
questionnaire.

## 3.1 Origin of parents

Calculate the linguistic distances and the exposure weights. Lacking
further actionable information among the questionnaire data about the
exposure to parents, the linguistic distance associated with a parent
that comes from outside the reference locality of the speaker receives
the maximal exposure weight (i.e. 1).

Participants were required to have at least one of their parents
coming from the region of the reference locality. However, it is not
rare for participants to have two local parents.

```
# Put UID, reference locality, SDS_code + influencing location copied into a data frame
df_Parents <- LMI_df %>%
  dplyr::select(UID,site_code, SDS_CODE_standard, 
         Origin_loc_father, Origin_canton_father, 
         Origin_loc_mother, Origin_canton_mother) %>% 
  mutate(Origin_father = paste0(Origin_loc_father, ", ", Origin_canton_father),
         Origin_mother = paste0(Origin_loc_mother, ", ", Origin_canton_mother)) %>% # the location is merged with its canton to ameliorate the automatic geocoding results
  dplyr::select(-c(Origin_loc_father, Origin_canton_father, Origin_loc_mother, Origin_canton_mother))
```

Through geocoding, we assign a linguistic distance between the
speaker and their parents.

We import a previously prepared dictionary for repairing geocoding
errors. This dictionary resulted from previous runs of the
`tidygeocoder` geocoding method and manual checks of
erroneous results. False geocoding stemmed mostly from imprecise data
entry by speakers into the SDATS questionnaire (e.g. typos, settlements
with the same name in different cantons or countries, too specific
locations within administrative areas etc.)

*A set of geocoded localities in and around
Switzerland, featuring the Voronoi-polygons of the SDS survey, based on
which they are assigned to an SDS survey locality*

Using this dictionary we change some of the place names among the
parents’ origins so that the geocoding will add the right
coordinates.

[The below code chunk is not run - as it runs for a long time. Its
result is later imported as a table.]

```
mapped <- read.csv("geocoded_factors.csv", header = T, stringsAsFactors = F) 

df_Parents$Origin_father <- plyr::mapvalues(df_Parents$Origin_father, from=mapped$loc, to=mapped$change_to)
df_Parents$Origin_mother <- plyr::mapvalues(df_Parents$Origin_mother, from=mapped$loc, to=mapped$change_to)

# spotwise change
df_Parents$Origin_father[which(df_Parents$UID=="XMZW")] <- "Blatten,VS"

# run the geocoder, resulting in latitude and longitude coordinates for both parents' places of origin
# one item takes 2-3 minutes on average
geocoded_mother_origin <- df_Parents %>%
  tidygeocoder::geocode(Origin_mother, method = 'arcgis', lat = lat_loc_mother , long = lon_loc_mother)
df_Parents <- geocoded_mother_origin %>%
  tidygeocoder::geocode(Origin_father, method = 'arcgis', lat = lat_loc_father, long = lon_loc_father)


#write.csv(df_Parents,"df_Parents_geocoded.csv")
```

Next, we solve the point-in-polygon problem for the coordinate pairs
provided by the geocoding: we find out in the Voronoi-polygon of which
SDS survey location the parents’ origin falls in.

```
#### import library needed for the point-in-polygon process
library(sf)
# if it does not load, try loading or reinstalling 'Rcpp' package first

# read in the csv file produced in the previous code chunk

df_Parents <- read.csv("df_Parents_geocoded.csv", header = T)

# read in the shapefiles of Germany and Austria
DEA_sf <- read_sf(dsn="Shapefiles/", layer="Germany_Austria") %>%
  st_transform(4326) %>% # we need to have the same coordinate reference system (CRS)
  dplyr::select(NAME_LONG) # remove unneeded coloumns


#read in SDS' Voronoi-polygons as geoJSON 
# the SDS codes ca not be brought to a standardised form yet, though
SDS_vor_sf <- read_sf("Shapefiles/sds_voronois.geojson") %>%
  st_transform(4326) %>%
  dplyr::select(SDS_CODE:SDS_KT)


SDS_vor_sf_valid <- st_make_valid(SDS_vor_sf) # validate the Voronoi-polygons

# convert parents' locations into sf objects
df_ParentsFather_sf <- as_tibble(df_Parents) %>%
  dplyr::select(-c(lon_loc_mother,lat_loc_mother)) %>%
  filter(!is.na(lon_loc_father)) %>% 
  sf::st_as_sf(
    coords= c("lon_loc_father", "lat_loc_father"),
    agr = "constant",
    crs=4326, # CRS of WGS84
    stringsAsFactors = FALSE,
    remove = TRUE
  ) 

# first, join places that are in Germany (DE) and Austria (A)
points_in_DEA<- st_join(df_ParentsFather_sf, DEA_sf, join = st_within)

# overwrite this by joining the results into the SDS Voronoi-polygons
parentsFather_in_SDS <- st_join(points_in_DEA, SDS_vor_sf_valid, join = st_within) %>%
  mutate(SDS_CODE.y = ifelse(NAME_LONG=="Austria" & is.na(SDS_CODE),"AU001",
                             ifelse(NAME_LONG=="Germany" & is.na(SDS_CODE),"DE001",
                                    ifelse(NAME_LONG=="Liechtenstein" & is.na(SDS_CODE),"FL001",
                                           ifelse(NAME_LONG=="Luxembourg" & is.na(SDS_CODE),"LX001",SDS_CODE))))
  )

# bring the SDS code to a standard form ('XY007' or 'XY123' rather than 'XY 7' and 'XY 123') to be able to make joins across data frames
parentsFather_in_SDS$SDS_CODE_x1 <- str_sub(parentsFather_in_SDS$SDS_CODE.y, 1,2)
parentsFather_in_SDS$SDS_CODE_x2 <- str_sub(parentsFather_in_SDS$SDS_CODE.y, 3,-1)

parentsFather_in_SDS <- as.data.frame(parentsFather_in_SDS) %>%
  mutate(SDS_CODE_x2 = str_pad(SDS_CODE_x2,3,"left","0")) %>%
  mutate(SDS_CODE = paste0(SDS_CODE_x1,SDS_CODE_x2)) %>%
  dplyr::select(-c(SDS_CODE_x1, SDS_CODE_x2, SDS_CODE.y))

# based on the two, now standard SDS codes we can search the dyadiclingDist table for the corresponding linguistic distances
# we do it in a loop. First we test if we are looking for a pair that contains the same SDS codes (self-pair) or a pair that includes NANA (which denote places in foreign, non-German-speaking countries). These pairs get a 0 distance, otherwise by combining to xors, we find the only row where the two SDS codes in two coloumns match
# This search runs very quickly

parentsFather_in_SDS$father_meanLingDist <- NA # initiate an empty coloumn
for (i in 1:nrow(parentsFather_in_SDS)) {
  if(parentsFather_in_SDS$SDS_CODE_standard[i]==parentsFather_in_SDS$SDS_CODE[i] | parentsFather_in_SDS$SDS_CODE[i]=="NANA") {
    parentsFather_in_SDS$father_meanLingDist[i] <- 0
  } else {
    parentsFather_in_SDS$father_meanLingDist[i] <- dyadicLingDists %>%
      filter(xor(origin==parentsFather_in_SDS$SDS_CODE_standard[i],
                 dest==parentsFather_in_SDS$SDS_CODE_standard[i])) %>% # behold XOR
      filter(xor(origin==parentsFather_in_SDS$SDS_CODE[i],
                 dest==parentsFather_in_SDS$SDS_CODE[i])) %>%
      dplyr::select(mean_lingDist)
  }
}

# the resulting value has to be unlisted
parentsFather_in_SDS$father_meanLingDist <- unlist(as.double(parentsFather_in_SDS$father_meanLingDist))

### Perform the same process for the mother's side too

df_ParentsMother_sf <- as_tibble(df_Parents) %>%
  dplyr::select(-c(lon_loc_father,lat_loc_father)) %>%
  filter(!is.na(lon_loc_mother)) %>% 
  sf::st_as_sf(
    coords= c("lon_loc_mother", "lat_loc_mother"),
    agr = "constant",
    crs=4326, # CRS of WGS84
    stringsAsFactors = FALSE,
    remove = TRUE
  ) 

points_in_DEA<- st_join(df_ParentsMother_sf, DEA_sf, join = st_within)

parentsMother_in_SDS <- st_join(points_in_DEA, SDS_vor_sf_valid, join = st_within) %>%
  mutate(SDS_CODE.y = ifelse(NAME_LONG=="Austria" & is.na(SDS_CODE),"AU001",
                             ifelse(NAME_LONG=="Germany" & is.na(SDS_CODE),"DE001",
                                    ifelse(NAME_LONG=="Liechtenstein" & is.na(SDS_CODE),"FL001",
                                           ifelse(NAME_LONG=="Luxembourg" & is.na(SDS_CODE),"LX001",SDS_CODE))))
  )
  
parentsMother_in_SDS$SDS_CODE_x1 <- str_sub(parentsMother_in_SDS$SDS_CODE.y, 1,2)
parentsMother_in_SDS$SDS_CODE_x2 <- str_sub(parentsMother_in_SDS$SDS_CODE.y, 3,-1)

parentsMother_in_SDS <- as.data.frame(parentsMother_in_SDS) %>%
  mutate(SDS_CODE_x2 = str_pad(SDS_CODE_x2,3,"left","0")) %>%
  mutate(SDS_CODE = paste0(SDS_CODE_x1,SDS_CODE_x2)) %>%
  dplyr::select(-c(SDS_CODE_x1, SDS_CODE_x2, SDS_CODE.y))

# assign the linguistic distances from dyadicLingDist list
parentsMother_in_SDS$mother_meanLingDist <- NA
for (i in 1:nrow(parentsMother_in_SDS)) {
  if(parentsMother_in_SDS$SDS_CODE_standard[i]==parentsMother_in_SDS$SDS_CODE[i] | parentsMother_in_SDS$SDS_CODE[i]=="NANA") {
    parentsMother_in_SDS$mother_meanLingDist[i] <- 0
  } else {
    parentsMother_in_SDS$mother_meanLingDist[i] <- dyadicLingDists %>%
      filter(xor(origin==parentsMother_in_SDS$SDS_CODE_standard[i],
                 dest==parentsMother_in_SDS$SDS_CODE_standard[i])) %>% # behold XOR
      filter(xor(origin==parentsMother_in_SDS$SDS_CODE[i],
                 dest==parentsMother_in_SDS$SDS_CODE[i])) %>%
      dplyr::select(mean_lingDist)
  }
}

parentsMother_in_SDS$mother_meanLingDist <- unlist(as.double(parentsMother_in_SDS$mother_meanLingDist))

# and save these distances into the result table we've set up earlier
LMI_dfEnd %<>%
  left_join(parentsMother_in_SDS %>% dplyr::select(UID, mother_meanLingDist),
            by= "UID") %>%
  left_join(parentsFather_in_SDS %>% dplyr::select(UID, father_meanLingDist),
            by= "UID")
```

## 3.2 Origin of partner

The questionnaire elicits information on the flatmates and the three
closest personal contacts of the speaker, which may contain mentions of
the long-term partner. Information was elicited about whether the
long-term partner, when mentioned, lives with the speaker. The duration
of the relationship, however, was not elicited.

The calculation of the linguistic distance happens similarly to the
parents. In addition, the exposure weight of the partner is determined
here.

The exposure weight differs depending on whether they live together
(0.5) or whether the partner is mentioned among the three closest
personal contacts of the speaker (0.3). For older speakers, living
together with a partner may mean there is a higher certainty that they
have been together for longer, accounting for more linguistic exposure.
Therefore, the weight is maximised in these cases (i.e. 1).

Note that we do not have the information on how long the speaker has
been together with the partner mentioned, therefore we cannot estimate
the exposure, only indirectly, based on age.

[The below code chunk is not run - as it runs for a long time. Its
result is later imported as a table.]

```
# Put UID, reference locality, SDS_code + influencing location copied into a data frame
# In addition Wohnsituation (residential situation , i.e. if the speaker lives alone, with a partner, with parents etc.), Herkunft (origin of the partner), Mitbewohner (flatmates), Privatperson1_3 (the three people from private life with whom the speaker communicates most often), Herkunft (origin of these people). The partner may appear among the flatmates or the communication partners.

# determine the exposure weight of the partner (if it is mentioned) depending on whether they live together or separately
LMI_dfEnd$weight_Partner <- ifelse(LMI_df$Housing_arrangements=="Mit (Ehe-)Partner/in",0.5, # case of living together
                                    ifelse(LMI_df$PrivatePerson1_relationship=="(Ehe-)Partner/in" |     
                                             LMI_df$PrivatePerson2_relationship=="(Ehe-)Partner/in" |
                                             LMI_df$PrivatePerson3_relationship=="(Ehe-)Partner/in",0.3,0))
# in the above three cases the partner is mentioned among the most frequently contacted private contacts.

# double the weight assigned in the previous step if the speaker is in the older age cohort, as that suggests a higher probability that they had been together for long with their partner.
for(i in 1:nrow(LMI_dfEnd)){ # loop through all speakers
  if(LMI_dfEnd$Age_cohort[i] == "older" && LMI_df$Housing_arrangements[i]=="Mit (Ehe-)Partner/in"){
    LMI_dfEnd$weight_Partner[i] <- 2*LMI_dfEnd$weight_Partner[i]
  }
}

df_Partner <- LMI_df %>%
  dplyr::select(UID,site_code, SDS_CODE_standard,Housing_arrangements,PrivatePerson1_relationship,PrivatePerson2_relationship,
                PrivatePerson3_relationship,
                Origin_Flatmate1, PrivatePerson1_Origin_loc, PrivatePerson1_Origin_canton,
                PrivatePerson2_Origin_loc, PrivatePerson2_Origin_canton,
                PrivatePerson3_Origin_loc, PrivatePerson3_Origin_canton) %>%
  mutate(Origin_partner = ifelse(Housing_arrangements =="Mit (Ehe-)Partner/in", 
                                   Origin_Flatmate1,
                                   ifelse(PrivatePerson1_relationship =="(Ehe-)Partner/in", 
                                          paste0(PrivatePerson1_Origin_loc, ", ", PrivatePerson1_Origin_canton),
                                          ifelse(PrivatePerson2_relationship =="(Ehe-)Partner/in", 
                                                 paste0(PrivatePerson2_Origin_loc, ", ", PrivatePerson2_Origin_canton), 
                                                 ifelse(PrivatePerson3_relationship =="(Ehe-)Partner/in",
                                                        paste0(PrivatePerson3_Origin_loc, ", ", PrivatePerson3_Origin_canton),
                                                        NA))))) %>% # find the partner among the connections and save their location
  dplyr::select(-c(Origin_Flatmate1, PrivatePerson1_Origin_loc, PrivatePerson1_Origin_canton,PrivatePerson2_Origin_loc,
                   PrivatePerson2_Origin_canton,PrivatePerson3_Origin_loc, PrivatePerson3_Origin_canton))

df_Partner$Origin_partner <- plyr::mapvalues(df_Partner$Origin_partner, from=mapped$loc, to=mapped$change_to)

# run the geocoder
df_Partner %<>%
  tidygeocoder::geocode(Origin_partner, method = 'arcgis', lat = lat_loc_partner , long = lon_loc_partner)

#write.csv(df_Partner, "df_Partner_geocoded.csv")
```

Import the table resulting from the previous chunk and assign the
linguistic distances similarly to the parents above.

```
df_Partner <- read.csv("df_Partner_geocoded.csv", header = T)

# convert partner' locations into sf
df_Partner_sf <- as_tibble(df_Partner) %>%
  filter(!is.na(lon_loc_partner)) %>% 
  sf::st_as_sf(
    coords= c("lon_loc_partner", "lat_loc_partner"),
    agr = "constant",
    crs=4326, # CRS of WGS84
    stringsAsFactors = FALSE,
    remove = TRUE
  ) 

points_in_DEA<- st_join(df_Partner_sf, DEA_sf, join = st_within)

partner_in_SDS <- st_join(points_in_DEA, SDS_vor_sf_valid, join = st_within) %>%
  mutate(SDS_CODE.y = ifelse(NAME_LONG=="Austria" & is.na(SDS_CODE),"AU001",
                             ifelse(NAME_LONG=="Germany" & is.na(SDS_CODE),"DE001",
                                    ifelse(NAME_LONG=="Liechtenstein" & is.na(SDS_CODE),"FL001",
                                           ifelse(NAME_LONG=="Luxembourg" & is.na(SDS_CODE),"LX001",SDS_CODE))))
  )

partner_in_SDS$SDS_CODE_x1 <- str_sub(partner_in_SDS$SDS_CODE.y, 1,2)
partner_in_SDS$SDS_CODE_x2 <- str_sub(partner_in_SDS$SDS_CODE.y, 3,-1)

partner_in_SDS <- as.data.frame(partner_in_SDS) %>%
  mutate(SDS_CODE_x2 = str_pad(SDS_CODE_x2,3,"left","0")) %>%
  mutate(SDS_CODE = paste0(SDS_CODE_x1,SDS_CODE_x2)) %>%
  dplyr::select(-c(SDS_CODE_x1, SDS_CODE_x2, SDS_CODE.y))

# assignment of the linguistic distance similarly to parents
partner_in_SDS$partner_meanLingDist <- NA
for (i in 1:nrow(partner_in_SDS)) {
  if(partner_in_SDS$SDS_CODE_standard[i]==partner_in_SDS$SDS_CODE[i] | partner_in_SDS$SDS_CODE[i]=="NANA") {
    partner_in_SDS$partner_meanLingDist[i] <- 0
  } else {
    partner_in_SDS$partner_meanLingDist[i] <- dyadicLingDists %>%
      filter(xor(origin==partner_in_SDS$SDS_CODE_standard[i],
                 dest==partner_in_SDS$SDS_CODE_standard[i])) %>% # behold XOR
      filter(xor(origin==partner_in_SDS$SDS_CODE[i],
                 dest==partner_in_SDS$SDS_CODE[i])) %>%
      dplyr::select(mean_lingDist)
  }
}

partner_in_SDS$partner_meanLingDist <- unlist(as.double(partner_in_SDS$partner_meanLingDist))

# and save these distances into the table already set up
LMI_dfEnd %<>%
  left_join(partner_in_SDS %>% dplyr::select(UID, partner_meanLingDist),
            by= "UID")
```

## 3.3 Residence outside the reference locality (external residence)

Calculate the linguistic distances and the exposure weights to places
the speaker has lived outside the reference locality.

In the questionnaire, it was possible to detail ten additional places
of residence. The amount of time spent at each of these localities is
given in the original questionnaire as categories.

Translation: - “3-11 Monate” = 3-11 months  
- “1-2 Jahre” = 1-2 years  
- “3-5 Jahre” = 3-5 years  
- “6-10 Jahre” = 6-10 years  
- “11-20 Jahre” = 11-20 years  
- “mehr als 20 Jahre” = more than 20 years

Based on the duration of residence, collected in a categorical
manner, weights of external residence increase logarithmically. 15 is
the middle of the category (11-20 years) by which we assume that the
effect of a residence at another location would reach its sill on
average. i.e. it may grow afterwards in reality, but we assume that the
full linguistic effect of living somewhere else is reached, also
considering that in most cases, SDATS speakers have not lived outside
their reference locality before the age of ~18-20. Living somewhere for
more than 20 years yields an additional +0.5 to the weight.

The values are calculated as *w = log(x+1)/log(15+1)*, where
*x* is the mid-point of the categories in years, and 15 years is
the mid-point of the category where the maximum value is reached. We
also have to make sure that the logarithmic curve starts from 0
(intercept). `log(1)=0`, thus we have to shift the scale to
the right and we place y=1 at 16, and shift all categories’ central
values to the right by 1. `log(16)`=2.772589.

Thus, the weights given below are calculated by
`log(x)/2.772589`

[The below code chunk is not run - as it takes a long time to run it.
Its result is later imported as a table.]

```
#copy the time spent as weights first
LMI_dfEnd$weight_AdditResidence1 <- LMI_df$AdditResidence1_duration
LMI_dfEnd$weight_AdditResidence2 <- LMI_df$AdditResidence2_duration
LMI_dfEnd$weight_AdditResidence3 <- LMI_df$AdditResidence3_duration
LMI_dfEnd$weight_AdditResidence4 <- LMI_df$AdditResidence4_duration
LMI_dfEnd$weight_AdditResidence5 <- LMI_df$AdditResidence5_duration
LMI_dfEnd$weight_AdditResidence6 <- LMI_df$AdditResidence6_duration
LMI_dfEnd$weight_AdditResidence7 <- LMI_df$AdditResidence7_duration
LMI_dfEnd$weight_AdditResidence8 <- LMI_df$AdditResidence8_duration
LMI_dfEnd$weight_AdditResidence9 <- LMI_df$AdditResidence9_duration
LMI_dfEnd$weight_AdditResidence10 <- LMI_df$AdditResidence10_duration

# recode time spent there as weight
LMI_dfEnd %<>%
  mutate_at(vars(contains("weight_Ad")), ~recode(.,
                                                 "3-11 Monate"= 0.1462406,
                                          "1-2 Jahre"= 0.330482,
                                          "3-5 Jahre"= 0.580482,
                                          "6-10 Jahre"= 0.7924812,
                                          "11-20 Jahre"= 1, # full estimated effect is reached
                                          "mehr als 20 Jahre" = 1.5, # higher weight but of course it is only available for older cohort, 32 cases in 500 people
                                          .missing = 0
                                        )) 

# select the coloumns needed for the geocoding
df_AdditResidence <- LMI_df %>%
  dplyr::select(UID,site_code, SDS_CODE_standard, 
         starts_with("Ad") & ends_with("name")) 

# change the location names that need change for the geocoding
df_AdditResidence[,4:13] <- apply(df_AdditResidence[,4:13], 2, plyr::mapvalues, from=mapped$loc, to=mapped$change_to)

# implement the above code (which parents and partner uses) in a for loop
# noone lived in 10 additional places, the only 9th place is also abroad, therefore for (z 1:9) is enough
for (z in 1:9) {
  #subset the AdditResidence table
  temp_df <- df_AdditResidence %>% 
    dplyr::select(UID,site_code,SDS_CODE_standard, paste0("AdditResidence",z,"_name")) %>%
    rename(coloumnInQuestion = last_col()) # rename the last coloumn
  
  temp_df %<>% # run the geocoder
    tidygeocoder::geocode(coloumnInQuestion, method = 'arcgis', lat = lat_loc_additRes , long = lon_loc_additRes)
  
  # convert additional location into sf
  df_AdditRes_sf <- as_tibble(temp_df) %>%
    filter(!is.na(lon_loc_additRes)) %>% 
    sf::st_as_sf(
      coords= c("lon_loc_additRes", "lat_loc_additRes"),
      agr = "constant",
      crs=4326, # CRS of WGS84
      stringsAsFactors = FALSE,
      remove = TRUE
    ) 
  
  # join first places in Germany and Austria
  points_in_DEA<- st_join(df_AdditRes_sf, DEA_sf, join = st_within)
  
  # overwrite this for Switzerland
  AdditRes_in_SDS <- st_join(points_in_DEA, SDS_vor_sf_valid, join = st_within) %>%
    mutate(SDS_CODE.y = ifelse(NAME_LONG=="Austria" & is.na(SDS_CODE),"AU001",
                               ifelse(NAME_LONG=="Germany" & is.na(SDS_CODE),"DE001",
                                      ifelse(NAME_LONG=="Liechtenstein" & is.na(SDS_CODE),"FL001",
                                             ifelse(NAME_LONG=="Luxembourg" & is.na(SDS_CODE),"LX001",SDS_CODE))))
    )
  
  # standardise the SDS code here too
  AdditRes_in_SDS$SDS_CODE_x1 <- str_sub(AdditRes_in_SDS$SDS_CODE.y, 1,2)
  AdditRes_in_SDS$SDS_CODE_x2 <- str_sub(AdditRes_in_SDS$SDS_CODE.y, 3,-1)

  AdditRes_in_SDS <- as.data.frame(AdditRes_in_SDS) %>%
    mutate(SDS_CODE_x2 = str_pad(SDS_CODE_x2,3,"left","0")) %>%
    mutate(SDS_CODE = paste0(SDS_CODE_x1,SDS_CODE_x2)) %>%
    dplyr::select(-c(SDS_CODE_x1, SDS_CODE_x2, SDS_CODE.y))
  
  # assignment of the linguistic distance
  AdditRes_in_SDS$AdditRes_meanLingDist <- NA
  for (i in 1:nrow(AdditRes_in_SDS)) {
    if(AdditRes_in_SDS$SDS_CODE_standard[i]==AdditRes_in_SDS$SDS_CODE[i] | AdditRes_in_SDS$SDS_CODE[i]=="NANA") {
      AdditRes_in_SDS$AdditRes_meanLingDist[i] <- 0
    } else {
      AdditRes_in_SDS$AdditRes_meanLingDist[i] <- dyadicLingDists %>%
        filter(xor(origin==AdditRes_in_SDS$SDS_CODE_standard[i],
                   dest==AdditRes_in_SDS$SDS_CODE_standard[i])) %>% # behold XOR
        filter(xor(origin==AdditRes_in_SDS$SDS_CODE[i],
                   dest==AdditRes_in_SDS$SDS_CODE[i])) %>%
        dplyr::select(mean_lingDist)
    }
  }
  
  AdditRes_in_SDS$AdditRes_meanLingDist <- unlist(as.double(AdditRes_in_SDS$AdditRes_meanLingDist))
  
  # and save these distances into the table already set up
  actualname <- paste0("AdditRes",z)
  LMI_dfEnd %<>%
    left_join(AdditRes_in_SDS %>% dplyr::select(UID, AdditRes_meanLingDist),
              by= "UID")
  colnames(LMI_dfEnd)[ncol(LMI_dfEnd)] <- actualname
  
}
```

## 3.4 Workplace

Calculate the linguistic distance and estimate an exposure weight to
the current workplace of the speaker.

The locality of the current workplace and duration of employment to
date were elicited. The linguistic distance associated with the
workplace receives a logarithmically growing weight, which reaches a
maximum after 10 years. The values are calculated as *w =
log(x+1)/log(10+1)*, where *x* is the years spent working at
the current locality, and 10 is used for reaching the maximum value
(i.e. 1). This is lower than for residences because here exact years are
given in the questionnaire. Also, workplace is mostly known for the
younger cohort only, which is less resistant to change than the old one
(Baxter & Croft, 2016). The value is weighted further by the
proportions of Swiss German and Standard German at work (elicited as a
percentage). Exposure to Standard German is counted as an exposure to a
place with 0.5 linguistic distance.

We do not take into account the number of days commuting also
elicited in the SDATS questionnaire, as COVID influenced the answers
through home office regulations at the times of recording SDATS, as seen
from several primary workplace commute days given as 0 or explicitly
mentioning home office.

[The below code chunk is not run - as it runs long. Its result is
later imported as a table.]

```
# Into a new data frame, subset reference locality, SDS code, name of the place of work and its canton, the years since working there, and the percentage of Swiss German and Standard German used at work
df_Workplace <- LMI_df %>%
  dplyr::select(UID,site_code, SDS_CODE_standard,
                OnlyWorkplace_loc,OnlyWorkplace_canton, Workplace1_loc, Workplace1_canton, Current_Workplace_duration, Language_work_CHD, Language_work_HD) %>%
  mutate(Workplace = ifelse(!is.na(OnlyWorkplace_loc),paste0(OnlyWorkplace_loc, ", ", OnlyWorkplace_canton),
                            ifelse(!is.na(Workplace1_loc), paste0(Workplace1_loc, ", ", Workplace1_canton), NA 
                            ))) %>% # determine the locality of the workplace
  dplyr::select(-c(OnlyWorkplace_loc, OnlyWorkplace_canton, Workplace1_loc, Workplace1_canton))

# change the location names that need change
df_Workplace$Workplace <- plyr::mapvalues(df_Workplace$Workplace, from=mapped$loc, to=mapped$change_to)

df_Workplace %<>%
    tidygeocoder::geocode(Workplace, method = 'arcgis', lat = lat_loc_workplace , long = lon_loc_workplace)

#write.csv(df_Workplace, "df_Workplace_geocoded.csv")
```

Import the resulting table and do the point-in-polygon method, and
assign the linguistic distances.

```
df_Workplace <- read.csv("df_Workplace_geocoded.csv", header = T)
# convert locations into sf
df_Workplace_sf <- as_tibble(df_Workplace) %>%
  filter(!is.na(lon_loc_workplace)) %>% 
  sf::st_as_sf(
    coords= c("lon_loc_workplace", "lat_loc_workplace"),
    agr = "constant",
    crs=4326, # CRS of WGS84
    stringsAsFactors = FALSE,
    remove = TRUE
  ) 

points_in_DEA<- st_join(df_Workplace_sf, DEA_sf, join = st_within)

workplace_in_SDS <- st_join(points_in_DEA, SDS_vor_sf_valid, join = st_within) %>%
  mutate(SDS_CODE.y = ifelse(NAME_LONG=="Austria" & is.na(SDS_CODE),"AU001",
                             ifelse(NAME_LONG=="Germany" & is.na(SDS_CODE),"DE001",
                                    ifelse(NAME_LONG=="Liechtenstein" & is.na(SDS_CODE),"FL001",
                                           ifelse(NAME_LONG=="Luxembourg" & is.na(SDS_CODE),"LX001",SDS_CODE))))
  )

workplace_in_SDS$SDS_CODE_x1 <- str_sub(workplace_in_SDS$SDS_CODE.y, 1,2)
workplace_in_SDS$SDS_CODE_x2 <- str_sub(workplace_in_SDS$SDS_CODE.y, 3,-1)

workplace_in_SDS <- as.data.frame(workplace_in_SDS) %>%
  mutate(SDS_CODE_x2 = str_pad(SDS_CODE_x2,3,"left","0")) %>%
  mutate(SDS_CODE = paste0(SDS_CODE_x1,SDS_CODE_x2)) %>%
  dplyr::select(-c(SDS_CODE_x1, SDS_CODE_x2, SDS_CODE.y))

workplace_in_SDS$workplace_meanLingDist <- NA
for (i in 1:nrow(workplace_in_SDS)) {
  if(workplace_in_SDS$SDS_CODE_standard[i]==workplace_in_SDS$SDS_CODE[i] | workplace_in_SDS$SDS_CODE[i]=="NANA") {
    workplace_in_SDS$workplace_meanLingDist[i] <- 0
  } else {
    workplace_in_SDS$workplace_meanLingDist[i] <- dyadicLingDists %>%
      filter(xor(origin==workplace_in_SDS$SDS_CODE_standard[i],
                 dest==workplace_in_SDS$SDS_CODE_standard[i])) %>% # behold XOR
      filter(xor(origin==workplace_in_SDS$SDS_CODE[i],
                 dest==workplace_in_SDS$SDS_CODE[i])) %>%
      dplyr::select(mean_lingDist)
  }
}

workplace_in_SDS$workplace_meanLingDist <- unlist(as.double(workplace_in_SDS$workplace_meanLingDist))


## ADD EXPOSURE WEIGHT

# weight it with a logarithmic curve which reaches a sill at 10 years. 
workplace_in_SDS$DurationMax10 <- ifelse(workplace_in_SDS$Current_Workplace_duration >=10, 
                                                 11,
                                                 workplace_in_SDS$Current_Workplace_duration+1)
#Shifting the curve, thus we add 1 . We divide the logarithm of the years spent at the company with the log of 11 (=2.397895). This way working there for 0 years does not count, 1 year counts already
workplace_in_SDS$weight_Workplace <- log(workplace_in_SDS$DurationMax10)/2.397895

#multiplying it by the percentage of Swiss German spoken at the workplace
workplace_in_SDS$weight_Workplace <- workplace_in_SDS$weight_Workplace*(workplace_in_SDS$Language_work_CHD/100)

# we add standard german as a weighted exposure to 0.5 (the flat rate we decided for a linguistic distance between all swiss german localities and standard german)
workplace_in_SDS$weight_Workplace <- workplace_in_SDS$weight_Workplace + 0.5*workplace_in_SDS$Language_work_HD/100

# keep it to max 1 
workplace_in_SDS$weight_Workplace[which(workplace_in_SDS$weight_Workplace>1)] <- 1


# put the weight coloumn into LMI_endDf
LMI_dfEnd <- LMI_dfEnd %>%
  left_join(workplace_in_SDS %>% dplyr::select(UID, workplace_meanLingDist, weight_Workplace, Current_Workplace_duration), 
            by= "UID")
```

## 3.5 Place of ongoing education

Calculate the linguistic distance and the exposure weight to the
place of education (if ongoing).

First, the linguistic distance is weighted by the days per week spent
at the institution (maximised in 5 days). The value is weighted further
by the proportion of Swiss German and Standard German at work (elicited
as a percentage). Exposure to Standard German is counted as an exposure
to a place with 0.5 linguistic distance.

[The below code chunk is not run - as it runs long. Its result is
later imported as a table.]

```
# Into a new data frame, subset reference locality, SDS code, working situation i.e. working, studying, both, neither or pensioner (Occupational_situation), the location of studies (Edu_loc), days per week at the institute (Edu_DaysPerWeek),  and the percentage of Swiss German and Standard German used at school (Language_edu_CHD, Language_edu_HD)
df_Eduplace <- LMI_df %>%
  dplyr::select(UID,site_code, SDS_CODE_standard, Occupational_situation,
         Edu_loc, Edu_DaysPerWeek,  Current_Workplace_duration, 
         Language_edu_CHD, Language_edu_HD)

df_Eduplace$Edu_loc <- plyr::mapvalues(df_Eduplace$Edu_loc, from=mapped$loc, to=mapped$change_to)


df_Eduplace %<>%
  tidygeocoder::geocode(Edu_loc, method = 'arcgis', lat = lat_loc_eduplace, long = lon_loc_eduplace)

#write.csv(df_Eduplace, "df_Eduplace_geocoded.csv")
```

```
df_Eduplace <- read.csv("df_Eduplace_geocoded.csv", header = T)

df_Eduplace_sf <- as_tibble(df_Eduplace) %>%
  filter(!is.na(lon_loc_eduplace)) %>% 
  sf::st_as_sf(
    coords= c("lon_loc_eduplace", "lat_loc_eduplace"),
    agr = "constant",
    crs=4326, # CRS of WGS84
    stringsAsFactors = FALSE,
    remove = TRUE
  ) 

points_in_DEA<- st_join(df_Eduplace_sf, DEA_sf, join = st_within)

eduplace_in_SDS <- st_join(points_in_DEA, SDS_vor_sf_valid, join = st_within) %>%
  mutate(SDS_CODE.y = ifelse(NAME_LONG=="Austria" & is.na(SDS_CODE),"AU001",
                             ifelse(NAME_LONG=="Germany" & is.na(SDS_CODE),"DE001",
                                    ifelse(NAME_LONG=="Liechtenstein" & is.na(SDS_CODE),"FL001",
                                           ifelse(NAME_LONG=="Luxembourg" & is.na(SDS_CODE),"LX001",SDS_CODE))))
  )

eduplace_in_SDS$SDS_CODE_x1 <- str_sub(eduplace_in_SDS$SDS_CODE.y, 1,2)
eduplace_in_SDS$SDS_CODE_x2 <- str_sub(eduplace_in_SDS$SDS_CODE.y, 3,-1)

eduplace_in_SDS <- as.data.frame(eduplace_in_SDS) %>%
  mutate(SDS_CODE_x2 = str_pad(SDS_CODE_x2,3,"left","0")) %>%
  mutate(SDS_CODE = paste0(SDS_CODE_x1,SDS_CODE_x2)) %>%
  dplyr::select(-c(SDS_CODE_x1, SDS_CODE_x2, SDS_CODE.y))


eduplace_in_SDS$eduplace_meanLingDist <- NA
for (i in 1:nrow(eduplace_in_SDS)) {
  if(eduplace_in_SDS$SDS_CODE_standard[i]==eduplace_in_SDS$SDS_CODE[i] | eduplace_in_SDS$SDS_CODE[i]=="NANA") {
    eduplace_in_SDS$eduplace_meanLingDist[i] <- 0
  } else {
    eduplace_in_SDS$eduplace_meanLingDist[i] <- dyadicLingDists %>%
      filter(xor(origin==eduplace_in_SDS$SDS_CODE_standard[i],
                 dest==eduplace_in_SDS$SDS_CODE_standard[i])) %>% # behold XOR
      filter(xor(origin==eduplace_in_SDS$SDS_CODE[i],
                 dest==eduplace_in_SDS$SDS_CODE[i])) %>%
      dplyr::select(mean_lingDist)
  }
}

eduplace_in_SDS$eduplace_meanLingDist <- unlist(as.double(eduplace_in_SDS$eduplace_meanLingDist))

### EXPOSURE WEIGHT
# Start with the number of days per week at the institute
eduplace_in_SDS$proportion <- eduplace_in_SDS$Edu_DaysPerWeek  
eduplace_in_SDS$proportion[which(eduplace_in_SDS$proportion >5)] <- 5 # maximise education days in 5 and divide it by 5
eduplace_in_SDS$proportion <- eduplace_in_SDS$proportion/5

#multiplying it by the percentage of Swiss German spoken at school
eduplace_in_SDS$weight_Eduplace <- eduplace_in_SDS$proportion*(eduplace_in_SDS$Language_edu_CHD/100)

# we add standard german as a weighted exposure to 0.5 (the flat rate we decided for a linguistic distance between all swiss german localities and standard german)
eduplace_in_SDS$weight_Eduplace <- eduplace_in_SDS$weight_Eduplace + 0.5*eduplace_in_SDS$Language_edu_HD/100


LMI_dfEnd %<>%
  left_join(eduplace_in_SDS %>% dplyr::select(UID, eduplace_meanLingDist,weight_Eduplace),
            by= "UID")
```

## 3.6 Spotwise changes

In some cases the reference locality was not placed correctly in the
corresponding SDS Voronoi polygons. We introduce a few reductions here
back to 0 in the case of Glarus, Igis, Sursee, Weinfelden, Blatten,
Alosen (Oberägeri).

Missing linguistic distances are also inserted by hand. These ones
are found by commands similar to the one in the below, hidden code.
[code chunk available in the corresponding .Rmd file]

[The below code chunk with spotwise changes also hidden, but it
runs.]

## 3.7 Combine the LMI components with the variables of dialectal change

Import the transcribed lexical variables.

```
#import dialect change data, the 10 items coded for the 500 speakers
coded_df <-  read.csv("Lexical_Change_SDStoSdats_500.csv", header = T, stringsAsFactors = F) %>% # have UID as first col and sort it according to that
  dplyr::select(UID, everything()) %>% # and order it based on UID
  arrange(UID)
```

And import the table we will use as input for setting up the LMI
prototypes (the product of all the previous chunks).

```
LMI_input<-read.csv("LMI_dfEnd.csv", header = T)
```

### 3.7.1 Sum up the amount of dialect change

```
AendSDS_SDATS_cols <- c("Aenderung_Butter",
                            "Aenderung_Kuss",
                            "Aenderung_Bonbon", 
                            "Aenderung_Zwiebel", 
                            "Aenderung_Wange",
                            "Aenderung_Schmetterling",
                            "Aenderung_Pfütze",
                            "Aenderung_Taschentuch",
                            "Aenderung_Sommersprossen",
                            "Aenderung_Schluckauf")

# count how many times we can assert the change, per row (there might be NA's which we do not need)
coded_df$n_potChange <- rowSums(!is.na(coded_df[AendSDS_SDATS_cols]))

coded_df %<>%
  rowwise() %>%
  mutate(sumChangeFromSDS = sum(c(Aenderung_Butter,
                            Aenderung_Kuss,
                            Aenderung_Bonbon, 
                            Aenderung_Zwiebel, 
                            Aenderung_Wange,
                            Aenderung_Schmetterling,
                            Aenderung_Pfütze,
                            Aenderung_Taschentuch,
                            Aenderung_Sommersprossen,
                            Aenderung_Schluckauf),
                         na.rm = TRUE)/n_potChange)
```

# 4 Determining the relational weights for agents in LMI

Due to the variable nature of the relationships and the uncertainties
coupled with the questionnaire data in our sample, we implement models
where we check the effect of the linguistic distances associated with
the agents in LMI, on the dialect change. The stronger this effect, the
more influence the agent has on the dialect change rate, therefore the
more it should count towards estimating the exposure to dialectal
varieties.

Relational weight stems from implicit information on the relation to
the people through which the speaker is exposed to the place of
interest.

We add a few repairs to the data first.

```
LMI_input$Sex[which(LMI_input$UID=="CDSL")] <- "Männlich" # for the time, let's have CDSL as a male (=männlich). They added maybe by mistake, maybe on purpose 'no answer' to the question on gender but thes represent a male speaker in the overview records of SDATS.
#for this study we have to code those people for whom originally it is undecided (thus NA) if their education background has tertiary education or not. Mostly it's unambiguous based on further information elicited
LMI_input$Edu_bkgr_2cat[which(LMI_input$UID=="VUYF")] <- "mit tertiärem Bildungsabschluss" # with tertiary education background
LMI_input$Edu_bkgr_2cat[which(LMI_input$UID=="WYIV")] <- "mit tertiärem Bildungsabschluss"
LMI_input$Edu_bkgr_2cat[which(LMI_input$UID=="SXMH")] <- "ohne tertiären Bildungsabschluss" # without tertiary education background
LMI_input$Edu_bkgr_2cat[which(LMI_input$UID=="RMNW")] <- "ohne tertiären Bildungsabschluss"
LMI_input$Edu_bkgr_2cat[which(LMI_input$UID=="BBCP")] <- "ohne tertiären Bildungsabschluss"
LMI_input$Edu_bkgr_2cat[which(LMI_input$UID=="GOSY")] <- "ohne tertiären Bildungsabschluss"
LMI_input$Edu_bkgr_2cat[which(LMI_input$UID=="JLQZ")] <- "ohne tertiären Bildungsabschluss"
LMI_input$Edu_bkgr_2cat[which(LMI_input$UID=="KSJX")] <- "ohne tertiären Bildungsabschluss"
LMI_input$Edu_bkgr_2cat[which(LMI_input$UID=="OASX")] <- "ohne tertiären Bildungsabschluss"
LMI_input$Edu_bkgr_2cat[which(LMI_input$UID=="WOZO")] <- "ohne tertiären Bildungsabschluss"
LMI_input$Edu_bkgr_2cat[which(LMI_input$UID=="XBSQ")] <- "ohne tertiären Bildungsabschluss"
LMI_input$Edu_bkgr_2cat[which(LMI_input$UID=="ZPGU")] <- "ohne tertiären Bildungsabschluss"

# we impute also for the 4-cat level
LMI_input$Edu_bkgr_4cat[which(LMI_input$UID=="VUYF")] <- "Höhere Berufsbildung (Meisterdiplom, HF, o.ä.)" # with tertiary education background
LMI_input$Edu_bkgr_4cat[which(LMI_input$UID=="WYIV")] <- "Höhere Berufsbildung (Meisterdiplom, HF, o.ä.)"
LMI_input$Edu_bkgr_4cat[which(LMI_input$UID=="SXMH")] <- "Berufslehre oder Berufsmaturität" # without tertiary education background
LMI_input$Edu_bkgr_4cat[which(LMI_input$UID=="RMNW")] <- "Berufslehre oder Berufsmaturität"
LMI_input$Edu_bkgr_4cat[which(LMI_input$UID=="BBCP")] <- "Berufslehre oder Berufsmaturität"
LMI_input$Edu_bkgr_4cat[which(LMI_input$UID=="GOSY")] <- "Berufslehre oder Berufsmaturität"
LMI_input$Edu_bkgr_4cat[which(LMI_input$UID=="JLQZ")] <- "Berufslehre oder Berufsmaturität"
LMI_input$Edu_bkgr_4cat[which(LMI_input$UID=="KSJX")] <- "Berufslehre oder Berufsmaturität"
LMI_input$Edu_bkgr_4cat[which(LMI_input$UID=="OASX")] <- "Berufslehre oder Berufsmaturität"
LMI_input$Edu_bkgr_4cat[which(LMI_input$UID=="WOZO")] <- "Berufslehre oder Berufsmaturität"
LMI_input$Edu_bkgr_4cat[which(LMI_input$UID=="XBSQ")] <- "Berufslehre oder Berufsmaturität"
LMI_input$Edu_bkgr_4cat[which(LMI_input$UID=="ZPGU")] <- "Berufslehre oder Berufsmaturität"
```

## 4.1 Find the relational weights through mixed-effects modelling

We find out the relational weights of the LMI components (mother,
father, partner, external residence, workplace, place of education) by
modelling the degree to which these agents (as unweighted linguistic
distances) affect the dialect change recorded in our sample speakers. In
order to do this, we:

1. Scale linguistic distances between 0 and their maxima
2. Aggregate linguistic distances related to external residences
3. Impute values for some of the components using *bootstrapped
   regression imputation* and based on further information
   available
4. Set up mixed-effects models for subsets of the sample to model the
   dialect change effects of different agents

Regarding the treatment of external residences, we have all (max. 10)
of them recorded in the speaker’s life.  
In contrast, we only have one value for workplace, if at all, and for
long-term partner, both of which might have not been the only one.

To be able to treat the external residences at the same scale, we
summarise their effect and weight them by the years spent there. Thus,
basically we use the exposure weighted result for the external
residences, summed.

Unite data from `coded_df` and
`LMI_input`.  
Scale it as specified above. Regarding the external residences, we
aggregate them as weighted averages, to have it on the same scale as
other agents.

```
df_forImput <- LMI_input %>% # select the needed coloumns
  dplyr::select(UID,site_code, Age_cohort,Sex, Edu_bkgr_2cat,Edu_bkgr_4cat,
         mother_meanLingDist,father_meanLingDist,partner_meanLingDist, workplace_meanLingDist, eduplace_meanLingDist, 
         matches("AdditRes\\d"),
         matches("AdditResidence\\d")) %>%  
  left_join(coded_df %>%  
              dplyr::select(UID, !!AendSDS_SDATS_cols,
                     sumChangeFromSDS),
            by="UID") %>% 
  rename("Aenderung_Pfuetze" = "Aenderung_Pfütze")
  
  
  # scale all ling dist values in between 0 and the maximal occurring value (/0.67722519)
df_forImput %<>% 
  mutate(mother_meanLingDist = mother_meanLingDist/0.67722519,
         father_meanLingDist = father_meanLingDist/0.67722519,
         partner_meanLingDist = partner_meanLingDist/0.67722519,
         workplace_meanLingDist = workplace_meanLingDist/0.67722519,
         eduplace_meanLingDist = eduplace_meanLingDist/0.67722519,
         AdditRes1 = AdditRes1/0.67722519,
         AdditRes2 = AdditRes2/0.67722519,
         AdditRes3= AdditRes3/0.67722519,
         AdditRes4= AdditRes4/0.67722519,
         AdditRes5 = AdditRes5/0.67722519,
         AdditRes6 = AdditRes6/0.67722519,
         AdditRes7 = AdditRes7/0.67722519,
         AdditRes8 = AdditRes8/0.67722519,
         AdditRes9 = AdditRes9/0.67722519,
         ) 

# multiply the distances to the external residences with the weight calculated based on the time spent there, and sum it  
df_forImput %<>%  
  mutate(LMI_AdditRes1 = AdditRes1*weight_AdditResidence1,
         LMI_AdditRes2 = AdditRes2*weight_AdditResidence2,
         LMI_AdditRes3 = AdditRes3*weight_AdditResidence3,
         LMI_AdditRes4 = AdditRes4*weight_AdditResidence4,
         LMI_AdditRes5 = AdditRes5*weight_AdditResidence5,
         LMI_AdditRes6 = AdditRes6*weight_AdditResidence6,
         LMI_AdditRes7 = AdditRes7*weight_AdditResidence7,
         LMI_AdditRes8 = AdditRes8*weight_AdditResidence8,
         LMI_AdditRes9 = AdditRes9*weight_AdditResidence9) %>% 
  rowwise() %>%
  mutate(LMI_Residence_total = sum(c(LMI_AdditRes1,LMI_AdditRes2 ,LMI_AdditRes3,LMI_AdditRes4,LMI_AdditRes5,LMI_AdditRes6,LMI_AdditRes7, LMI_AdditRes8,LMI_AdditRes9), na.rm = TRUE)) %>% # reorder some coloumns
  dplyr::select(sumChangeFromSDS, everything(), -c(contains("AdditRes")), -c(contains("weight")))

ggplot(data=df_forImput) + geom_jitter(aes(x=LMI_Residence_total, y=sumChangeFromSDS, color=Age_cohort), alpha=0.5)+
  xlab("External residence LMI component") + ylab("Dialect change rate")
```

```
# write.csv(df_forImput, "inputForImputation.csv")
```

Thus the external residence predictor is still on the same scale as
other predictors in the below models, but possibly outside their
bounds.

#### 4.1.0.1 Imputing the missing values into df\_forImput

We impute some missing values so the later mixed models determining
the relational weights will not have to skip certain speakers due to
`NA`‘s. We use bootstrapped regression imputation to impute
values for the ’partner’s linguistic distance’ and the ‘workplace
linguistic distance’.

Basically, those speakers with information about their workplace
unavailable (pensioners), should not influence the weight ‘linguistic
distance to workplace’ gets (same goes for place of education),
therefore a model is needed with subsets of only these people. Besides,
partners actually represent to some degree peers, in the sense that we
have no information about the origins of most peers of our speakers, and
we do not know how long the partner that we have information about has
exerted their influence. Surely there is peer influence and possibly
there are partner influences that our questionnaire does not catch. Thus
imputing partners for those that did not note one in the questionnaire
(due to being single at the moment or the partner having deceased),
makes sense if we think of partners as the representation of
peer-effect. Thus we impute partners also for those speakers that did
not indicate having one.

In the first step we impute that those speakers that study only
possibly have not ever worked yet so the ‘workplace linguistic distance’
is set to 0.

```
library(mice)
set.seed(7778)

df_forImput <- read.csv("inputForImputation.csv", header=T)%>% 
# imputing 0 for student's workplace ling dist - having no work means that it should not have affected language change
  mutate(workplace_meanLingDist = ifelse(is.na(workplace_meanLingDist) & !is.na(eduplace_meanLingDist),
                                         0,
                                         workplace_meanLingDist)
         )
```

Repairing a few values that were ruined through the update of the
tidygeocoder package for the final version of the supplementary
material.

```
df_forImput[239,"mother_meanLingDist"] <- 0.13660693  
df_forImput[325,"LMI_Residence_total"]<-0.40520460  
df_forImput[399,"partner_meanLingDist"]<-0.22251233  
df_forImput[481,"partner_meanLingDist"]<-0.5978235  
df_forImput[147,"partner_meanLingDist"]<-0.7384934
```

Impute ‘partner linguistic distance’ and ‘workplace linguistic
distance’ together, making sure that the imputed values stay between the
0,1 interval.

```
imp_input <- df_forImput %>% 
  select(UID,mother_meanLingDist, father_meanLingDist, partner_meanLingDist, workplace_meanLingDist, LMI_Residence_total) 

imp <- mice(imp_input, method = "norm.boot",m = 1) # Impute data for partner and workplace simultaneously
```

```
## 
##  iter imp variable
##   1   1  partner_meanLingDist  workplace_meanLingDist
##   2   1  partner_meanLingDist  workplace_meanLingDist
##   3   1  partner_meanLingDist  workplace_meanLingDist
##   4   1  partner_meanLingDist  workplace_meanLingDist
##   5   1  partner_meanLingDist  workplace_meanLingDist
```

```
# setting up a postprocessing restriction for mice, to stop mice from imputing anything outside 0 and 1
post <- imp$post
post[c("partner_meanLingDist","workplace_meanLingDist")] <- "imp[[j]][, i] <- squeeze(imp[[j]][, i], c(0, 1))"

# imputation again with the actual postprocessing restriction
imp <- mice(imp_input, method = "norm.boot", post=post,m = 1)
```

```
## 
##  iter imp variable
##   1   1  partner_meanLingDist  workplace_meanLingDist
##   2   1  partner_meanLingDist  workplace_meanLingDist
##   3   1  partner_meanLingDist  workplace_meanLingDist
##   4   1  partner_meanLingDist  workplace_meanLingDist
##   5   1  partner_meanLingDist  workplace_meanLingDist
```

```
data_boot <- complete(imp) # Store data
```

Plot the imputed values (red) of ‘partner linguistic distance’ with
the existing ones and a histogram.

[The code is not public, for the protection of the SDATS
participants’ anonimity]

Plot the imputed values (red) of ‘workplace linguistic distance’ with
the existing ones and a histogram.

[The code is not public, for the protection of the SDATS
participants’ anonimity]

It is visible that each coloumn in the histogram grows, thus the
distribution of the values does not change a lot

Put the coloumns of ‘partner linguistic distance’ and ‘workplace
linguistic distance’ as new coloumns back into the original table. When
we imported it, the 0 workplace distances were already imputed for
students.

```
df_forImput %<>%
  left_join(toplot %>% select(UID,partner_lingdist_imp = partner_meanLingDist.y, workplace_lingdist_imp = workplace_meanLingDist.y), by="UID")
```

#### 4.1.0.2 Preparing long table for modelling

We need a long table with all dialect data utterance of each speaker
as a row and then we will take different subsets, and do different mixed
models (UID and item will be the random effects).

```
df_forRelWModel_longer <- df_forImput %>%
  pivot_longer(cols=c(Aenderung_Butter:Aenderung_Schluckauf), 
               names_to = "Item",
               values_to = "Change") %>%
  filter(!is.na(Change)) %>%
  mutate(UID = as.factor(UID),
         Edu_bkgr_2cat = as.factor(Edu_bkgr_2cat),
         Edu_bkgr_4cat = as.factor(Edu_bkgr_4cat),
         Sex = as.factor(Sex),
         Age_cohort = as.factor(Age_cohort),
         Item = as.factor(Item))

# change item names
df_forRelWModel_longer %<>%
  mutate(Item_EN = dplyr::recode(Item,
                                 "Aenderung_Butter" = "butter", 
                                 "Aenderung_Kuss" = "kiss",
                                 "Aenderung_Bonbon" = "candy",
                                 "Aenderung_Zwiebel" = "onion",
                                 "Aenderung_Wange" = "cheek",
                                 "Aenderung_Schmetterling" = "butterfly",
                                 "Aenderung_Pfuetze" = "puddle",
                                 "Aenderung_Taschentuch" = "tissue",
                                 "Aenderung_Sommersprossen" = "freckles",
                                 "Aenderung_Schluckauf" = "hickup"
  ))
  
levels(df_forRelWModel_longer$Item_EN) <- c("butter","butterfly","candy","cheek","freckles","hickup","kiss","onion","puddle", "tissue")
# write.csv(df_forRelWModel_longer, "inputForRelationalWeightModelling.csv")
```

## 4.2 Models for relational weights

For these models all speakers are used. We use the imputed values of
the partners and workplace (it is either 0 if we know that the speaker
is only studying, or it is imputed as seen above), and we do not use
‘education place ling dist’. We control for the variables used as
criteria for recruiting participants i.e. *age cohort, sex and
educational background (split in two categories)* and use Item and
UID as grouping effects (random effects).

We set up four mixed-effect models for estimating relational weights
and from each of them we use the beta effect estimate for the agents for
which each speaker in the give model subsample has a value assigned.

### 4.2.1 Full general model

Out of the results of this model, we only use the effect associated
with the mother, father and external residences.

```
df_forRelWModel_longer <- read.csv("inputForRelationalWeightModelling.csv", header = T)

fullmodel <- glmer(Change ~ mother_meanLingDist + father_meanLingDist + partner_lingdist_imp + LMI_Residence_total + workplace_lingdist_imp +
                  Age_cohort + 
                  Sex +
                  Edu_bkgr_2cat +
                  (1|UID) + (1|Item_EN), 
                data=df_forRelWModel_longer, family=binomial(),
                control = glmerControl(optimizer = "bobyqa")
)

summ(fullmodel, digits = 4)
```

|  |  |
| --- | --- |
| Observations | 4983 |
| Dependent variable | Change |
| Type | Mixed effects generalized linear model |
| Family | binomial |
| Link | logit |

|  |  |
| --- | --- |
| AIC | 6128.0759 |
| BIC | 6199.7276 |
| Pseudo-R² (fixed effects) | 0.0510 |
| Pseudo-R² (total) | 0.1962 |

| Fixed Effects | | | | |
| --- | --- | --- | --- | --- |
|  | Est. | S.E. | z val. | p |
| (Intercept) | -1.1444 | 0.2193 | -5.2187 | 0.0000 |
| mother\_meanLingDist | 0.6351 | 0.1540 | 4.1242 | 0.0000 |
| father\_meanLingDist | 0.3170 | 0.2124 | 1.4924 | 0.1356 |
| partner\_lingdist\_imp | 0.2938 | 0.1668 | 1.7616 | 0.0781 |
| LMI\_Residence\_total | 0.0319 | 0.1387 | 0.2302 | 0.8180 |
| workplace\_lingdist\_imp | 0.3723 | 0.1958 | 1.9009 | 0.0573 |
| Age\_cohortyounger | 0.7581 | 0.0774 | 9.8004 | 0.0000 |
| SexWeiblich | -0.1972 | 0.0779 | -2.5303 | 0.0114 |
| Edu\_bkgr\_2catohne tertiären Bildungsabschluss | 0.1262 | 0.0829 | 1.5216 | 0.1281 |

| Random Effects | | |
| --- | --- | --- |
| Group | Parameter | Std. Dev. |
| UID | (Intercept) | 0.4673 |
| Item\_EN | (Intercept) | 0.6131 |

| Grouping Variables | | |
| --- | --- | --- |
| Group | # groups | ICC |
| UID | 500 | 0.0562 |
| Item\_EN | 10 | 0.0968 |

Standard error is often high, ruining the statistic significnance,
but in any case, the estimates are averages.

Out of these results, we only use the effect associated with the
**mother, father and the external residences.**

#### 4.2.1.1 Ten-fold cross-validation

```
library(cv)
```

```
## Warning: package 'cv' was built under R version 4.3.2
```

```
## Loading required package: doParallel
```

```
## Warning: package 'doParallel' was built under R version 4.3.2
```

```
## Loading required package: foreach
```

```
## Warning: package 'foreach' was built under R version 4.3.2
```

```
## Loading required package: iterators
```

```
## Warning: package 'iterators' was built under R version 4.3.2
```

```
## Loading required package: parallel
```

```
var(df_forRelWModel_longer$Change)
```

```
## [1] 0.237754
```

```
cv::cv(fullmodel, k=10, clusterVariables="UID", seed=5240)
```

```
## R RNG seed set to 5240
```

```
## 10-Fold Cross Validation based on 500 {UID} clusters
## cross-validation criterion = 0.2302788
## bias-adjusted cross-validation criterion = 0.2302104
## full-sample criterion = 0.2291337
```

The sample variance (SD^2) of Change in the data, 0.237754 to which
we can compare the cross-fold average MSE (Mean Squared Error). An MSE
lower than the variance of the target variable suggests that the model
is providing predictions that are, on average, better than simply
predicting the mean of the ‘Change’ variable for all observations. Here
we actually only want to know if there may be outlying groups thus
whether the modelling is accurate for our purposes.

The MSE value is smaller than the Variance value. It means that our
model captures some of the underlying patterns in the data and provides
predictions that deviate less from the true values than a naive approach
(like predicting the mean for all observations).

#### 4.2.1.2 Cohort-based models

The estimate about external residence is low possibly because it is
the older people (who show less language change) that have had the
chance to reside elsewhere longer, therefore it shows less effect.

Because of this, we run the model in a partitioned fashion too to
find out about the effect estimates in the two age cohorts
separately.

##### 4.2.1.2.1 Older cohort

```
df_forRelWModel_longer_old <- df_forRelWModel_longer %>% filter(Age_cohort=="older")
df_forRelWModel_longer_young <- df_forRelWModel_longer %>% filter(Age_cohort=="younger")

fullmodel_old <- glmer(Change ~ mother_meanLingDist + father_meanLingDist + partner_lingdist_imp + LMI_Residence_total + workplace_lingdist_imp +
                  #Age_cohort + 
                  Sex +
                  Edu_bkgr_2cat +
                  (1|UID) + (1|Item_EN), 
                data=df_forRelWModel_longer_old, 
                family=binomial(),
                control = glmerControl(optimizer = "bobyqa")
)

summ(fullmodel_old, digits = 4)
```

|  |  |
| --- | --- |
| Observations | 2491 |
| Dependent variable | Change |
| Type | Mixed effects generalized linear model |
| Family | binomial |
| Link | logit |

|  |  |
| --- | --- |
| AIC | 2962.8420 |
| BIC | 3021.0464 |
| Pseudo-R² (fixed effects) | 0.0203 |
| Pseudo-R² (total) | 0.1292 |

| Fixed Effects | | | | |
| --- | --- | --- | --- | --- |
|  | Est. | S.E. | z val. | p |
| (Intercept) | -1.1905 | 0.2014 | -5.9125 | 0.0000 |
| mother\_meanLingDist | 1.0636 | 0.2291 | 4.6418 | 0.0000 |
| father\_meanLingDist | 0.5284 | 0.2954 | 1.7885 | 0.0737 |
| partner\_lingdist\_imp | 0.4130 | 0.2218 | 1.8621 | 0.0626 |
| LMI\_Residence\_total | 0.0206 | 0.1658 | 0.1240 | 0.9013 |
| workplace\_lingdist\_imp | -0.0616 | 0.3207 | -0.1922 | 0.8476 |
| SexWeiblich | -0.0542 | 0.1191 | -0.4545 | 0.6495 |
| Edu\_bkgr\_2catohne tertiären Bildungsabschluss | 0.0630 | 0.1267 | 0.4977 | 0.6187 |

| Random Effects | | |
| --- | --- | --- |
| Group | Parameter | Std. Dev. |
| UID | (Intercept) | 0.4097 |
| Item\_EN | (Intercept) | 0.4934 |

| Grouping Variables | | |
| --- | --- | --- |
| Group | # groups | ICC |
| UID | 250 | 0.0454 |
| Item\_EN | 10 | 0.0658 |

##### 4.2.1.2.2 10-fold cross-validation

```
var(df_forRelWModel_longer_old$Change)
```

```
## [1] 0.2133406
```

```
cv::cv(fullmodel_old, k=10, clusterVariables="UID", seed=5240)
```

```
## R RNG seed set to 5240
```

```
## 10-Fold Cross Validation based on 250 {UID} clusters
## cross-validation criterion = 0.2122342
## bias-adjusted cross-validation criterion = 0.2121315
## full-sample criterion = 0.2103722
```

##### 4.2.1.2.3 Younger cohort

```
fullmodel_young <- glmer(Change ~ mother_meanLingDist + father_meanLingDist + partner_lingdist_imp + LMI_Residence_total + workplace_lingdist_imp +
                  #Age_cohort + 
                  Sex +
                  Edu_bkgr_2cat +
                  (1|UID) + (1|Item_EN), 
                data=df_forRelWModel_longer_young, 
                family=binomial(),
                control = glmerControl(optimizer = "bobyqa")
)

summ(fullmodel_young, digits = 4)
```

|  |  |
| --- | --- |
| Observations | 2492 |
| Dependent variable | Change |
| Type | Mixed effects generalized linear model |
| Family | binomial |
| Link | logit |

|  |  |
| --- | --- |
| AIC | 3129.7164 |
| BIC | 3187.9248 |
| Pseudo-R² (fixed effects) | 0.0155 |
| Pseudo-R² (total) | 0.2558 |

| Fixed Effects | | | | |
| --- | --- | --- | --- | --- |
|  | Est. | S.E. | z val. | p |
| (Intercept) | -0.3049 | 0.3177 | -0.9596 | 0.3372 |
| mother\_meanLingDist | 0.3395 | 0.2125 | 1.5980 | 0.1100 |
| father\_meanLingDist | 0.0222 | 0.3050 | 0.0728 | 0.9419 |
| partner\_lingdist\_imp | 0.2718 | 0.2520 | 1.0784 | 0.2808 |
| LMI\_Residence\_total | 0.1663 | 0.2650 | 0.6276 | 0.5302 |
| workplace\_lingdist\_imp | 0.6317 | 0.2563 | 2.4650 | 0.0137 |
| SexWeiblich | -0.3118 | 0.1110 | -2.8100 | 0.0050 |
| Edu\_bkgr\_2catohne tertiären Bildungsabschluss | 0.1661 | 0.1195 | 1.3909 | 0.1643 |

| Random Effects | | |
| --- | --- | --- |
| Group | Parameter | Std. Dev. |
| UID | (Intercept) | 0.5036 |
| Item\_EN | (Intercept) | 0.8995 |

| Grouping Variables | | |
| --- | --- | --- |
| Group | # groups | ICC |
| UID | 250 | 0.0583 |
| Item\_EN | 10 | 0.1859 |

##### 4.2.1.2.4 10-fold cross-validation

```
var(df_forRelWModel_longer_young$Change)
```

```
## [1] 0.2491942
```

```
cv::cv(fullmodel_young, k=10, clusterVariables="UID", seed=5240)
```

```
## R RNG seed set to 5240
```

```
## 10-Fold Cross Validation based on 250 {UID} clusters
## cross-validation criterion = 0.2495723
## bias-adjusted cross-validation criterion = 0.249374
## full-sample criterion = 0.2463797
```

For the cohort-based LMI prototype, we will use these estimates,
respectively.

It also makes sense that for the younger cohort parents’ origin shows
a smaller effect, as the language change might have been inherited by
them rather than happened during their (so far shorter) lifespan.
External residence shows a larger effect, assumedly because the younger
cohort has had less time to live somewhere else but once they did, its
effect shows more easily, due to the adolescent peak of language change
(Baxter & Croft, 2016).

### 4.2.2 Education model

Subset of speakers with an actual ‘education linguistic distance’,
i.e. those speakers that currently study (*n*=119). We use the
imputed values for the partners and for work (it is either 0 if we know
that the speaker is only studying, or imputed as seen above). We control
for the variables age cohort, sex and educational background and use
item and UID as grouping effects (random effects).

Out of the results, we only use the effect associated with the
**place of** **education**.

```
dataformodel <- df_forRelWModel_longer %>% filter(!is.na(eduplace_meanLingDist))

edu_model <- glmer(Change ~ 
                     mother_meanLingDist + father_meanLingDist + partner_lingdist_imp + LMI_Residence_total + workplace_lingdist_imp + eduplace_meanLingDist +
                  Age_cohort + 
                  Sex +
                  Edu_bkgr_2cat +
                  (1|UID) + (1|Item_EN), 
                data=dataformodel,
                family=binomial(),
                control = glmerControl(optimizer = "bobyqa")
)

summ(edu_model, digits = 4)
```

|  |  |
| --- | --- |
| Observations | 1186 |
| Dependent variable | Change |
| Type | Mixed effects generalized linear model |
| Family | binomial |
| Link | logit |

|  |  |
| --- | --- |
| AIC | 1512.8434 |
| BIC | 1573.7835 |
| Pseudo-R² (fixed effects) | 0.0187 |
| Pseudo-R² (total) | 0.2706 |

| Fixed Effects | | | | |
| --- | --- | --- | --- | --- |
|  | Est. | S.E. | z val. | p |
| (Intercept) | -0.2216 | 0.7276 | -0.3046 | 0.7607 |
| mother\_meanLingDist | 0.1232 | 0.3444 | 0.3576 | 0.7206 |
| father\_meanLingDist | 0.8697 | 0.4655 | 1.8682 | 0.0617 |
| partner\_lingdist\_imp | 0.2496 | 0.4122 | 0.6054 | 0.5449 |
| LMI\_Residence\_total | 0.0597 | 0.4824 | 0.1237 | 0.9015 |
| workplace\_lingdist\_imp | 0.6255 | 0.4063 | 1.5394 | 0.1237 |
| eduplace\_meanLingDist | 0.2023 | 0.3888 | 0.5202 | 0.6029 |
| Age\_cohortyounger | 0.0069 | 0.6737 | 0.0103 | 0.9918 |
| SexWeiblich | -0.1821 | 0.1722 | -1.0577 | 0.2902 |
| Edu\_bkgr\_2catohne tertiären Bildungsabschluss | -0.1150 | 0.2082 | -0.5526 | 0.5806 |

| Random Effects | | |
| --- | --- | --- |
| Group | Parameter | Std. Dev. |
| UID | (Intercept) | 0.5829 |
| Item\_EN | (Intercept) | 0.8925 |

| Grouping Variables | | |
| --- | --- | --- |
| Group | # groups | ICC |
| UID | 119 | 0.0768 |
| Item\_EN | 10 | 0.1800 |

#### 4.2.2.1 10-fold cross-validation

```
var(dataformodel$Change)
```

```
## [1] 0.2499541
```

```
cv::cv(edu_model, k=10, clusterVariables="UID", seed=5240)
```

```
## R RNG seed set to 5240
```

```
## 10-Fold Cross Validation based on 119 {UID} clusters
## cross-validation criterion = 0.2520112
## bias-adjusted cross-validation criterion = 0.2516625
## full-sample criterion = 0.2466516
```

Importantly for this cohort the origins of the father make a decisive
difference (however, we do not use that).

### 4.2.3 Partner model

Subset of speakers which gave ‘partner linguistic distance’
(*n*= 351). We use the imputed values for work (it is either 0 if
we know that the speaker is only studying, or imputed as seen above) and
we do not use ‘education linguistic distance’ as a predictor in this
model.

Out of the results, we only use the effect associated with the
partner.

```
dataformodel <- df_forRelWModel_longer %>% filter(!is.na(partner_meanLingDist))

partner_model <- glmer(Change ~ mother_meanLingDist + father_meanLingDist + partner_meanLingDist + LMI_Residence_total + workplace_lingdist_imp +
                  Age_cohort + 
                  Sex +
                  Edu_bkgr_2cat +
                  (1|UID) + (1|Item_EN), 
                data=dataformodel,
                family=binomial(),
                control = glmerControl(optimizer = "bobyqa")
)

summ(partner_model, digits = 4)
```

|  |  |
| --- | --- |
| Observations | 3498 |
| Dependent variable | Change |
| Type | Mixed effects generalized linear model |
| Family | binomial |
| Link | logit |

|  |  |
| --- | --- |
| AIC | 4287.2985 |
| BIC | 4355.0579 |
| Pseudo-R² (fixed effects) | 0.0534 |
| Pseudo-R² (total) | 0.1791 |

| Fixed Effects | | | | |
| --- | --- | --- | --- | --- |
|  | Est. | S.E. | z val. | p |
| (Intercept) | -1.1686 | 0.2164 | -5.4003 | 0.0000 |
| mother\_meanLingDist | 0.8682 | 0.1820 | 4.7694 | 0.0000 |
| father\_meanLingDist | 0.0631 | 0.2677 | 0.2356 | 0.8137 |
| partner\_meanLingDist | 0.3461 | 0.1861 | 1.8592 | 0.0630 |
| LMI\_Residence\_total | -0.0147 | 0.1529 | -0.0959 | 0.9236 |
| workplace\_lingdist\_imp | 0.2236 | 0.2320 | 0.9639 | 0.3351 |
| Age\_cohortyounger | 0.7744 | 0.0886 | 8.7363 | 0.0000 |
| SexWeiblich | -0.1066 | 0.0910 | -1.1715 | 0.2414 |
| Edu\_bkgr\_2catohne tertiären Bildungsabschluss | 0.0848 | 0.0948 | 0.8953 | 0.3706 |

| Random Effects | | |
| --- | --- | --- |
| Group | Parameter | Std. Dev. |
| UID | (Intercept) | 0.4016 |
| Item\_EN | (Intercept) | 0.5852 |

| Grouping Variables | | |
| --- | --- | --- |
| Group | # groups | ICC |
| UID | 351 | 0.0425 |
| Item\_EN | 10 | 0.0903 |

#### 4.2.3.1 10-fold cross-validation

```
var(dataformodel$Change)
```

```
## [1] 0.2343884
```

```
cv::cv(partner_model, k=10, clusterVariables="UID", seed=5240)
```

```
## R RNG seed set to 5240
```

```
## 10-Fold Cross Validation based on 351 {UID} clusters
## cross-validation criterion = 0.2271217
## bias-adjusted cross-validation criterion = 0.2270248
## full-sample criterion = 0.2254399
```

#### 4.2.3.2 Cohort-based models

We run the model in a partitioned fashion too to find out about the
effect estimates in the two cohorts separately.

##### 4.2.3.2.1 Older cohort

```
dataformodel_old <- dataformodel %>% filter(Age_cohort=="older")
dataformodel_young <- dataformodel %>% filter(Age_cohort=="younger")

partner_model_old <- glmer(Change ~ mother_meanLingDist + father_meanLingDist + partner_meanLingDist + LMI_Residence_total + workplace_lingdist_imp +
                  #Age_cohort + 
                  Sex +
                  Edu_bkgr_2cat +
                  (1|UID) + (1|Item_EN), 
                data=dataformodel_old, 
                family=binomial(),
                control = glmerControl(optimizer = "bobyqa")
)

summ(partner_model_old, digits = 4)
```

|  |  |
| --- | --- |
| Observations | 1983 |
| Dependent variable | Change |
| Type | Mixed effects generalized linear model |
| Family | binomial |
| Link | logit |

|  |  |
| --- | --- |
| AIC | 2339.6679 |
| BIC | 2395.5916 |
| Pseudo-R² (fixed effects) | 0.0268 |
| Pseudo-R² (total) | 0.1331 |

| Fixed Effects | | | | |
| --- | --- | --- | --- | --- |
|  | Est. | S.E. | z val. | p |
| (Intercept) | -1.2755 | 0.2099 | -6.0775 | 0.0000 |
| mother\_meanLingDist | 1.3060 | 0.2578 | 5.0664 | 0.0000 |
| father\_meanLingDist | 0.3157 | 0.3528 | 0.8948 | 0.3709 |
| partner\_meanLingDist | 0.4508 | 0.2454 | 1.8368 | 0.0662 |
| LMI\_Residence\_total | -0.0250 | 0.1842 | -0.1355 | 0.8922 |
| workplace\_lingdist\_imp | 0.0093 | 0.3415 | 0.0273 | 0.9783 |
| SexWeiblich | 0.0239 | 0.1357 | 0.1762 | 0.8601 |
| Edu\_bkgr\_2catohne tertiären Bildungsabschluss | 0.0600 | 0.1383 | 0.4338 | 0.6645 |

| Random Effects | | |
| --- | --- | --- |
| Group | Parameter | Std. Dev. |
| UID | (Intercept) | 0.3946 |
| Item\_EN | (Intercept) | 0.4976 |

| Grouping Variables | | |
| --- | --- | --- |
| Group | # groups | ICC |
| UID | 199 | 0.0422 |
| Item\_EN | 10 | 0.0671 |

##### 4.2.3.2.2 10-fold cross-validation

```
var(dataformodel_old$Change)
```

```
## [1] 0.211526
```

```
cv::cv(partner_model_old, k=10, clusterVariables="UID", seed=5240)
```

```
## R RNG seed set to 5240
```

```
## 10-Fold Cross Validation based on 199 {UID} clusters
## cross-validation criterion = 0.2098807
## bias-adjusted cross-validation criterion = 0.2097554
## full-sample criterion = 0.207554
```

##### 4.2.3.2.3 Younger cohort

```
partner_model_young <- glmer(Change ~ mother_meanLingDist + father_meanLingDist + partner_meanLingDist + LMI_Residence_total + workplace_lingdist_imp +
                  #Age_cohort + 
                  Sex +
                  Edu_bkgr_2cat +
                  (1|UID) + (1|Item_EN), 
                data=dataformodel_young, 
                family=binomial(),
                control = glmerControl(optimizer = "bobyqa")
)

summ(partner_model_young, digits = 4)
```

|  |  |
| --- | --- |
| Observations | 1515 |
| Dependent variable | Change |
| Type | Mixed effects generalized linear model |
| Family | binomial |
| Link | logit |

|  |  |
| --- | --- |
| AIC | 1939.7538 |
| BIC | 1992.9855 |
| Pseudo-R² (fixed effects) | 0.0127 |
| Pseudo-R² (total) | 0.2126 |

| Fixed Effects | | | | |
| --- | --- | --- | --- | --- |
|  | Est. | S.E. | z val. | p |
| (Intercept) | -0.2939 | 0.3104 | -0.9468 | 0.3437 |
| mother\_meanLingDist | 0.3878 | 0.2645 | 1.4661 | 0.1426 |
| father\_meanLingDist | -0.4463 | 0.4090 | -1.0912 | 0.2752 |
| partner\_meanLingDist | 0.3944 | 0.2878 | 1.3705 | 0.1705 |
| LMI\_Residence\_total | 0.1652 | 0.3004 | 0.5499 | 0.5824 |
| workplace\_lingdist\_imp | 0.4363 | 0.3204 | 1.3617 | 0.1733 |
| SexWeiblich | -0.2028 | 0.1310 | -1.5485 | 0.1215 |
| Edu\_bkgr\_2catohne tertiären Bildungsabschluss | 0.1107 | 0.1414 | 0.7829 | 0.4337 |

| Random Effects | | |
| --- | --- | --- |
| Group | Parameter | Std. Dev. |
| UID | (Intercept) | 0.3707 |
| Item\_EN | (Intercept) | 0.8352 |

| Grouping Variables | | |
| --- | --- | --- |
| Group | # groups | ICC |
| UID | 152 | 0.0333 |
| Item\_EN | 10 | 0.1691 |

##### 4.2.3.2.4 10-fold cross-validation

```
var(dataformodel_young$Change)
```

```
## [1] 0.2491396
```

```
cv::cv(partner_model_young, k=10, clusterVariables="UID", seed=5240)
```

```
## R RNG seed set to 5240
```

```
## 10-Fold Cross Validation based on 152 {UID} clusters
## cross-validation criterion = 0.2503295
## bias-adjusted cross-validation criterion = 0.2501099
## full-sample criterion = 0.2466887
```

### 4.2.4 Workplace model

Subset of speakers which have a ‘workplace linguistic distance’
(*n*= 306). We use the imputed values for partners (imputed as
seen above) and we do not use ‘education ling dist’.

The only exception is for ‘workplace linguistic distance’: those that
only study get an imputed 0 here as in case of these people we can
assume that they have not worked yet.

Out of the results, we only use the effect associated with the
workplace.

```
dataformodel <- df_forRelWModel_longer %>% filter(!is.na(workplace_meanLingDist))

workplace_model <- glmer(Change ~ mother_meanLingDist + father_meanLingDist + partner_lingdist_imp + LMI_Residence_total + workplace_meanLingDist +
                  Age_cohort + 
                  Sex +
                  Edu_bkgr_2cat +
                  (1|UID) + (1|Item_EN), 
                data=dataformodel,
                family=binomial(),
                control = glmerControl(optimizer = "bobyqa")
)

summ(workplace_model, digits = 4)
```

|  |  |
| --- | --- |
| Observations | 3051 |
| Dependent variable | Change |
| Type | Mixed effects generalized linear model |
| Family | binomial |
| Link | logit |

|  |  |
| --- | --- |
| AIC | 3795.6319 |
| BIC | 3861.8873 |
| Pseudo-R² (fixed effects) | 0.0394 |
| Pseudo-R² (total) | 0.2425 |

| Fixed Effects | | | | |
| --- | --- | --- | --- | --- |
|  | Est. | S.E. | z val. | p |
| (Intercept) | -1.0979 | 0.2976 | -3.6888 | 0.0002 |
| mother\_meanLingDist | 0.5006 | 0.2032 | 2.4641 | 0.0137 |
| father\_meanLingDist | 0.3118 | 0.2723 | 1.1451 | 0.2522 |
| partner\_lingdist\_imp | 0.2434 | 0.2278 | 1.0685 | 0.2853 |
| LMI\_Residence\_total | 0.1538 | 0.2315 | 0.6645 | 0.5063 |
| workplace\_meanLingDist | 0.4728 | 0.2405 | 1.9656 | 0.0493 |
| Age\_cohortyounger | 0.7742 | 0.1359 | 5.6976 | 0.0000 |
| SexWeiblich | -0.2669 | 0.1010 | -2.6431 | 0.0082 |
| Edu\_bkgr\_2catohne tertiären Bildungsabschluss | 0.1015 | 0.1082 | 0.9383 | 0.3481 |

| Random Effects | | |
| --- | --- | --- |
| Group | Parameter | Std. Dev. |
| UID | (Intercept) | 0.5171 |
| Item\_EN | (Intercept) | 0.7841 |

| Grouping Variables | | |
| --- | --- | --- |
| Group | # groups | ICC |
| UID | 306 | 0.0641 |
| Item\_EN | 10 | 0.1474 |

```
# 10-fold cross-validation
var(dataformodel$Change)
```

```
## [1] 0.246223
```

```
cv::cv(workplace_model, k=10, clusterVariables="UID", seed=5240)
```

```
## R RNG seed set to 5240
```

```
## 10-Fold Cross Validation based on 306 {UID} clusters
## cross-validation criterion = 0.2417336
## bias-adjusted cross-validation criterion = 0.2416073
## full-sample criterion = 0.2396904
```

#### 4.2.4.1 Cohort-based models

We run the model in a partitioned fashion too to find out about the
effect estimates in the two cohorts separately.

##### 4.2.4.1.1 Older cohort

```
dataformodel_old <- dataformodel %>% filter(Age_cohort=="older")
dataformodel_young <- dataformodel %>% filter(Age_cohort=="younger")

workplace_model_old <- glmer(Change ~ mother_meanLingDist + father_meanLingDist + partner_lingdist_imp + LMI_Residence_total + workplace_meanLingDist +
                  #Age_cohort + 
                  Sex +
                  Edu_bkgr_2cat +
                  (1|UID) + (1|Item_EN), 
                data=dataformodel_old, 
                family=binomial(),
                control = glmerControl(optimizer = "bobyqa")
)

summ(workplace_model_old, digits = 4)
```

|  |  |
| --- | --- |
| Observations | 579 |
| Dependent variable | Change |
| Type | Mixed effects generalized linear model |
| Family | binomial |
| Link | logit |

|  |  |
| --- | --- |
| AIC | 690.2060 |
| BIC | 733.8190 |
| Pseudo-R² (fixed effects) | 0.0512 |
| Pseudo-R² (total) | 0.1731 |

| Fixed Effects | | | | |
| --- | --- | --- | --- | --- |
|  | Est. | S.E. | z val. | p |
| (Intercept) | -1.0065 | 0.3172 | -3.1730 | 0.0015 |
| mother\_meanLingDist | 2.0136 | 0.6037 | 3.3354 | 0.0009 |
| father\_meanLingDist | 0.6547 | 0.5493 | 1.1919 | 0.2333 |
| partner\_lingdist\_imp | 0.2000 | 0.4991 | 0.4007 | 0.6887 |
| LMI\_Residence\_total | -0.1315 | 0.4732 | -0.2778 | 0.7811 |
| workplace\_meanLingDist | -0.8728 | 0.7531 | -1.1590 | 0.2465 |
| SexWeiblich | 0.0402 | 0.2619 | 0.1536 | 0.8780 |
| Edu\_bkgr\_2catohne tertiären Bildungsabschluss | -0.3585 | 0.2802 | -1.2796 | 0.2007 |

| Random Effects | | |
| --- | --- | --- |
| Group | Parameter | Std. Dev. |
| UID | (Intercept) | 0.4315 |
| Item\_EN | (Intercept) | 0.5466 |

| Grouping Variables | | |
| --- | --- | --- |
| Group | # groups | ICC |
| UID | 58 | 0.0493 |
| Item\_EN | 10 | 0.0792 |

##### 4.2.4.1.2 10-fold cross-validation

```
var(dataformodel_old$Change)
```

```
## [1] 0.2119392
```

```
cv::cv(workplace_model_old, k=10, clusterVariables="UID", seed=5240)
```

```
## R RNG seed set to 5240
```

```
## 10-Fold Cross Validation based on 58 {UID} clusters
## cross-validation criterion = 0.2124925
## bias-adjusted cross-validation criterion = 0.2120058
## full-sample criterion = 0.2041742
```

##### 4.2.4.1.3 Younger cohort

```
workplace_model_young <- glmer(Change ~ mother_meanLingDist + father_meanLingDist + partner_lingdist_imp + LMI_Residence_total + workplace_meanLingDist +
                  #Age_cohort + 
                  Sex +
                  Edu_bkgr_2cat +
                  (1|UID) + (1|Item_EN), 
                data=dataformodel_young, 
                family=binomial(),
                control = glmerControl(optimizer = "bobyqa")
)

summ(workplace_model_young, digits = 4)
```

|  |  |
| --- | --- |
| Observations | 2472 |
| Dependent variable | Change |
| Type | Mixed effects generalized linear model |
| Family | binomial |
| Link | logit |

|  |  |
| --- | --- |
| AIC | 3108.2701 |
| BIC | 3166.3980 |
| Pseudo-R² (fixed effects) | 0.0141 |
| Pseudo-R² (total) | 0.2536 |

| Fixed Effects | | | | |
| --- | --- | --- | --- | --- |
|  | Est. | S.E. | z val. | p |
| (Intercept) | -0.2995 | 0.3168 | -0.9454 | 0.3444 |
| mother\_meanLingDist | 0.3193 | 0.2153 | 1.4831 | 0.1380 |
| father\_meanLingDist | 0.0714 | 0.3082 | 0.2316 | 0.8168 |
| partner\_lingdist\_imp | 0.2476 | 0.2524 | 0.9810 | 0.3266 |
| LMI\_Residence\_total | 0.1873 | 0.2651 | 0.7063 | 0.4800 |
| workplace\_meanLingDist | 0.6061 | 0.2567 | 2.3612 | 0.0182 |
| SexWeiblich | -0.2975 | 0.1112 | -2.6763 | 0.0074 |
| Edu\_bkgr\_2catohne tertiären Bildungsabschluss | 0.1519 | 0.1197 | 1.2690 | 0.2044 |

| Random Effects | | |
| --- | --- | --- |
| Group | Parameter | Std. Dev. |
| UID | (Intercept) | 0.5017 |
| Item\_EN | (Intercept) | 0.8965 |

| Grouping Variables | | |
| --- | --- | --- |
| Group | # groups | ICC |
| UID | 248 | 0.0579 |
| Item\_EN | 10 | 0.1849 |

##### 4.2.4.1.4 10-fold cross-validation

```
var(dataformodel_young$Change)
```

```
## [1] 0.2491556
```

```
cv::cv(workplace_model_young, k=10, clusterVariables="UID", seed=5240)
```

```
## R RNG seed set to 5240
```

```
## 10-Fold Cross Validation based on 248 {UID} clusters
## cross-validation criterion = 0.2495657
## bias-adjusted cross-validation criterion = 0.2493773
## full-sample criterion = 0.2465791
```

#### 4.2.4.2 Recap of the models for relational weights

As a consequence of these models, for the LMI models we will be using
using the following multipliers, except the age-cohort-based prototype
(as mentioned above):

**mother\_meanLingDist** `0.63506`  
**father\_meanLingDist** `0.31703`  
**partner\_meanLingDist** `0.34607`  
**LMI\_Residence\_total** `0.03193`  
**workplace\_meanLingDist** `0.4728`  
**eduplace\_meanLingDist** `0.202255`

These multipliers affect the LMI of a person only if the specific
agent is actually present in the biographical dataset of the speaker
(i.e. if a speaker does not mention a partner or a workplace in the
questionnaire, we do not use the values imputed before the modelling,
but disregard the agent).

# 5 Going forward with LMI

Based on the `LMI_input` dataframe, through the
multiplication of the linguistic distances and the exposure weights, we
set up the LMI components, based on which the LMI prototypes are
assembled in **Supplementary Material Part 2**.

In the next code chunks we scale all linguistic distances between 0
and the maximal occurring value `0.67722519`. Then we set up
the components of the LMI by multiplying the linguistic distances with
the **exposure weights**, except for the parents where,
lacking further data about the intensity of the relationship, the full
linguistic distance is kept.

```
dat <- LMI_input %>% # impute 0 as workplace linguistic distance for those that only study (n=17)
  mutate(workplace_meanLingDist = ifelse(is.na(workplace_meanLingDist) & !is.na(eduplace_meanLingDist),
                                         0,
                                         workplace_meanLingDist)
         )

# scale all ling dist values in between 0 and the maximal occurring value (/0.67722519)
dat %<>% 
  mutate(mother_meanLingDist = mother_meanLingDist/0.67722519,
         father_meanLingDist = father_meanLingDist/0.67722519,
         partner_meanLingDist = partner_meanLingDist/0.67722519,
         workplace_meanLingDist = workplace_meanLingDist/0.67722519,
         eduplace_meanLingDist = eduplace_meanLingDist/0.67722519,
         AdditRes1 = AdditRes1/0.67722519,
         AdditRes2 = AdditRes2/0.67722519,
         AdditRes3= AdditRes3/0.67722519,
         AdditRes4= AdditRes4/0.67722519,
         AdditRes5 = AdditRes5/0.67722519,
         AdditRes6 = AdditRes6/0.67722519,
         AdditRes7 = AdditRes7/0.67722519,
         AdditRes8 = AdditRes8/0.67722519,
         AdditRes9 = AdditRes9/0.67722519,
         ) 
  
dat %<>%  # mutate weights and distance into the _components_ of LMI
  mutate(LMI_partner = partner_meanLingDist*weight_Partner,
         LMI_workplace = workplace_meanLingDist*weight_Workplace,
         LMI_eduplace = eduplace_meanLingDist*weight_Eduplace,
         LMI_AdditRes1 = AdditRes1*weight_AdditResidence1,
         LMI_AdditRes2 = AdditRes2*weight_AdditResidence2,
         LMI_AdditRes3 = AdditRes3*weight_AdditResidence3,
         LMI_AdditRes4 = AdditRes4*weight_AdditResidence4,
         LMI_AdditRes5 = AdditRes5*weight_AdditResidence5,
         LMI_AdditRes6 = AdditRes6*weight_AdditResidence6,
         LMI_AdditRes7 = AdditRes7*weight_AdditResidence7,
         LMI_AdditRes8 = AdditRes8*weight_AdditResidence8,
         LMI_AdditRes9 = AdditRes9*weight_AdditResidence9) %>% # select coloumns needed
  dplyr::select(UID,site_code, Age_cohort,Sex,Occupational_situation, Edu_bkgr_2cat,
         mother_meanLingDist,father_meanLingDist,LMI_partner, LMI_workplace, LMI_eduplace,
         LMI_AdditRes1, LMI_AdditRes2, LMI_AdditRes3, LMI_AdditRes4, LMI_AdditRes5, LMI_AdditRes6, LMI_AdditRes7, LMI_AdditRes8, LMI_AdditRes9) %>%  # add coloumns from coded_df
  left_join(LMI_df %>%
              dplyr::select(UID,Current_Workplace_duration),
            by="UID") %>%
  left_join(coded_df %>% 
              dplyr::select(UID, !!AendSDS_SDATS_cols,
                     sumChangeFromSDS),
            by="UID") %>% 
  dplyr::rename("Aenderung_Pfuetze" = "Aenderung_Pfütze")

# recode agents that have 'exotic' characters in them
dat %<>%
  mutate(Sex = recode(Sex,
                             "Männlich" = "M" ,
                            "Weiblich" = "F"
                             ),
         Occupational_situation = recode(Occupational_situation,
                                  "Ich bin im Ruhestand." = "pensioner",
                                  "Ich arbeite und bin auch in Ausbildung (z.B. berufstätig und in Weiterbildung, StudentIn mit Nebenjob, etc.)" = "work_study",
                                 "Ich arbeite und befinde mich&nbsp;  nicht  &nbsp;in einer Ausbildung." = "only_work",
                                 "Ich befinde mich in einer Ausbildung und arbeite&nbsp; nicht ." = "only_study"))


# unite the effects of external residences


dat$LMI_AdditRes_total <- rowSums(dat %>% dplyr::select(LMI_AdditRes1,LMI_AdditRes2 ,LMI_AdditRes3,LMI_AdditRes4,LMI_AdditRes5,LMI_AdditRes6,LMI_AdditRes7, LMI_AdditRes8,LMI_AdditRes9), na.rm = TRUE)

# reorder some coloumns
dat <- dat %>% 
  dplyr::select(sumChangeFromSDS, everything(), -c(LMI_AdditRes1,LMI_AdditRes2,LMI_AdditRes3,LMI_AdditRes4,LMI_AdditRes5, LMI_AdditRes6,LMI_AdditRes7,LMI_AdditRes8,LMI_AdditRes9))

# saving this out to be used in Part 2
# write.csv(dat,"df_global.csv")
```

A separate table is set up for the cumulative LMI prototype used in
**Supplementary Material Part 2**.

```
df_cumul  <- LMI_input

# scale all ling dist values in between 0 and the maximal occurring value (/0.67722519)
df_cumul %<>% 
  mutate(mother_meanLingDist = mother_meanLingDist/0.67722519,
         father_meanLingDist = father_meanLingDist/0.67722519,
         partner_meanLingDist = partner_meanLingDist/0.67722519,
         workplace_meanLingDist = workplace_meanLingDist/0.67722519,
         eduplace_meanLingDist = eduplace_meanLingDist/0.67722519,
         AdditRes1 = AdditRes1/0.67722519,
         AdditRes2 = AdditRes2/0.67722519,
         AdditRes3= AdditRes3/0.67722519,
         AdditRes4= AdditRes4/0.67722519,
         AdditRes5 = AdditRes5/0.67722519,
         AdditRes6 = AdditRes6/0.67722519,
         AdditRes7 = AdditRes7/0.67722519,
         AdditRes8 = AdditRes8/0.67722519,
         AdditRes9 = AdditRes9/0.67722519,
         ) 

df_cumul %<>%
  mutate(Sex = recode(Sex,
                             "Männlich" = "M" ,
                            "Weiblich" = "F"
                             ),
         Occupational_situation = recode(Occupational_situation,
                                  "Ich bin im Ruhestand." = "pensioner",
                                  "Ich arbeite und bin auch in Ausbildung (z.B. berufstätig und in Weiterbildung, StudentIn mit Nebenjob, etc.)" = "work_study",
                                 "Ich arbeite und befinde mich&nbsp;  nicht  &nbsp;in einer Ausbildung." = "only_work",
                                 "Ich befinde mich in einer Ausbildung und arbeite&nbsp; nicht ." = "only_study"))

df_cumul <- df_cumul %>% # keep only the linguistic distances without exposure weights
  dplyr::select(UID,site_code, Age_cohort,Sex,Occupational_situation, Edu_bkgr_2cat,
         mother_meanLingDist,father_meanLingDist,partner_meanLingDist,workplace_meanLingDist,eduplace_meanLingDist, AdditRes1, AdditRes2, AdditRes3, AdditRes4, AdditRes5, AdditRes6, AdditRes7, AdditRes8, AdditRes9 ) %>%  
  left_join(coded_df %>% 
              dplyr::select(UID, !!AendSDS_SDATS_cols, sumChangeFromSDS),
            by="UID") %>% 
  dplyr::rename("Aenderung_Pfuetze" = "Aenderung_Pfütze") 

# saving this out to be used in Part 2
# write.csv(df_cumul,"df_cumul.csv")
```

# 6 References:

Baxter, G. J. and Croft, W. (2016) ‘Modeling language change across
the lifespan: Individual trajectories in community change’, *Language
Variation and Change*, 28(2), pp. 129–173. doi:
10.1017/S0954394516000077

Jeszenszky, P., Steiner, C., & Leemann, A. (2021). Reduction of
Survey Sites in Dialectology: A New Methodology Based on Clustering.
*Frontiers in Artificial Intelligence*, 4(642505), 1–23. https://doi.org/10.3389/frai.2021.642505

Leemann, A., Jeszenszky, P., Steiner, C., Studerus, M., &
Messerli, J. (2020). SDATS Corpus – Swiss German dialects across time
and space. Retrieved from osf.io/s9z4q

Scherrer, Y. (2021) ‘dialektkarten.ch - Interactive dialect maps for
German-speaking Switzerland and other European dialect areas’, in
Krefeld, T., Lücke, S., and Mutter, C. (eds) *Berichte aus der
digitalen Geolinguistik (II): Akten der zweiten Arbeitstagung des
DFG-Langfristvorhabens VerbaAlpina und seiner Kooperationspartner am
18.06.2019*. Munich: Korpus im Text, University of Munich.

SDS = Hotzenköcherle, Rudolf & Baumgartner, Heinrich.
(1962-2003). Sprachatlas der deutschen Schweiz. Bern: Francke (Vols.
1-6), Basel: Francke (Vols. 7, 8).

SDATS = Leemann, A., Jeszenszky, P., Steiner, C., Studerus, M., &
Messerli, J. (2020). SDATS Corpus – Swiss German dialects across time
and space. Retrieved from osf.io/s9z4q
