# Supplementary material for: Effects of mobility on dialect change: Introducing the linguistic mobility index
Source: PLoS One. 2024 Apr 16;19(4):e0300735. doi: 10.1371/journal.pone.0300735 (PMC11020475; doi:10.1371/journal.pone.0300735)
Supplement: S2 Appendix — The report presents the composition of the four LMI prototypes evaluated in this article, conducting the statistical tests and modelling reported here including their summary results. The reproducible code for both reports can be accessed at https://osf.io/hfbpk/. (HTML) [file pone.0300735.s002.html]

Effects of mobility on dialect change: Introducing the Linguistic Mobility Index. Part 2 - Mixed-effect models


Code 

- Show All Code
- Hide All Code

# Effects of mobility on dialect change: Introducing the Linguistic Mobility Index. Part 2 - Mixed-effect models

#### Péter Jeszenszky, Carina Steiner, Adrian Leemann

#### 2024-03-01

# 1 Preface

While **Supplementary Material Part 1** describes the
construction of the **Linguistic Mobility Index** and its
components using data from the SDATS survey (Leemann et al., 2020), in
the present **Supplementary Material Part 2**, four LMI
prototypes are composed, after which they are evaluated by testing them
in mixed-effect models with dialect change rate as the outcome variable.
Click on the `Code` button in the top right to show the code.
Some code chunks are still hidden, but they can be followed in the
`.Rmd` file of the same name.

# 2 Importing data and setting up a table for modelling

## 2.1 Data import

Import the two tables resulting from **Supplementary Material
Part 1**, containing the LMI components, dialect change data and
the survey design variables.

```
LMI_globals <- read.csv("df_global.csv", header = T) 
LMI_cumul <- read.csv("df_cumul.csv",  header = T)
```

## 2.2 Setting up the four LMI prototypes

We will use the following relational weights, where relevant, as
calculated in **Supplementary Material Part 1**. These
multipliers affect the LMI of a person only if the specific agent is
actually present in the biographical dataset of the speaker.

**mother\_meanLingDist** `0.63506`  
**father\_meanLingDist** `0.31703`  
**partner\_meanLingDist** `0.34607`  
**LMI\_Residence\_total** `0.03193`  
**workplace\_meanLingDist** `0.4728`  
**eduplace\_meanLingDist** `0.202255`

Composition of the four LMI prototypes with relational weights
are indicated numerically.


|  |  |  |  | agent | **LMIA** | **LMIB** | **LMIC** | **LMID** | |
| --- | --- | --- | --- | --- |
| **Mother’s origin** | Ling. dist.\* 0.6351 | Ling. dist.\* 0.6351 | Ling. dist.\* 0.6351 | Ling. dist.\* 0.6351 |
| **Father’s origin** | Ling. dist.\* 0.317 | Ling. dist.\* 0.317 | Ling. dist.\* 0.317 | Ling. dist.\* 0.317 |
| **Partner’s origin** | - | Ling. Dist. \* 0.3461 | Ling. Dist. \* *weightexposure \** 0.3461 | Ling. Dist. \* *weightexposure \** 0.3461 |
| **External residence** | - | Σ Ling. Dist. \* 0.0319 | Σ Ling. Dist. \* *weightexposure \** 0.0319 | *Older cohort*:  Σ Ling. Dist. \* *weightexposure \** 0.0206 |
|  |  |  |  | *Younger cohort:* ΣLing. Dist. \* *weightexposure \** 0.1663 |
| **Workplace** | - | Ling. Dist. \* 0.4728 | Ling. Dist. \* *weightexposure \** 0.4728 | *Older cohort:* Ling. Dist. \* *weightexposure \* -*0.8728 |
|  |  |  |  | *Younger cohort:* Ling. Dist. \* *weightexposure \** 0.6061 |
| **Place of education** | - | Ling. Dist. \* 0.2023 | Ling. Dist. \* *weightexposure \** 0.2023 | Ling. Dist. \* *weightexposure \** 0.2023 |

### 2.2.1 **LMIA** (minimal prototype)

Considering only the origins of the parents.
**`LMI_minimal`** is used for this prototype in
the code and LMI\_A in the text.

Set up LMI\_A and standardise it.

```
LMI_globals %<>%
  rowwise() %>% # sum the linguistic distances of mother's and father's origins, weighted by the relational weight 
  mutate(LMI_minimal_unscaled = sum(mother_meanLingDist*0.63506,
           father_meanLingDist*0.31703,
           na.rm = TRUE)) 

#standardisation
LMI_globals$LMI_minimal <- (LMI_globals$LMI_minimal_unscaled - mean(LMI_globals$LMI_minimal_unscaled)) / (2*sd(LMI_globals$LMI_minimal_unscaled))
```

### 2.2.2 **LMIB** (cumulative prototype)

**`LMI_cumulated`** is used for this
prototype in the code and LMI\_B in the text.

LMI\_B cumulates, in a linear manner, the linguistically influential
agents weighted by their relational weights only (disregarding their
exposure weights). Thereby we admittedly lessen the effect of family and
strengthening the effect of peers and possible experiences connected to
areas outside the participant’s reference locality. This prototype
essentially checks if counting the number of influential agents in the
biographical data is a meaningful idea. Thus it simulates a survey with
some information about the locations in the speaker’s linguistic
biography, but not enough biographical data to fine-tune the
quantification (e.g. by calculating exposure weights).

For the older cohort in SDATS, place of education and workplace are
usually not recorded, therefore LMI\_B values may be biased.

Set up LMI\_B and standardise it.

```
LMI_cumul %<>%
  rowwise()%>%
  mutate(AdditRes_cumul = sum(AdditRes1, AdditRes2, AdditRes3, AdditRes4, AdditRes5, AdditRes6, AdditRes7, AdditRes8, AdditRes9,
                            na.rm = TRUE)) #cumulate linguistic distances belonging to additional places lived


# set up LMI^B
LMI_cumul %<>%
  mutate(LMI_cumul_unscaled = sum( mother_meanLingDist*0.63506,
                                   father_meanLingDist*0.31703,
                                   partner_meanLingDist*0.34607,
                                   AdditRes_cumul*0.03193,
                                   workplace_meanLingDist*0.4728,
                                   eduplace_meanLingDist*0.202255,
                                   na.rm = TRUE))

# z-standardisation of  LMI^B using Gelman & Hill's (2006:55-57) recommendation
LMI_cumul$LMI_cumulated <- (LMI_cumul$LMI_cumul_unscaled - mean(LMI_cumul$LMI_cumul_unscaled)) / (2*sd(LMI_cumul$LMI_cumul_unscaled))
```

### 2.2.3 **LMIC** (comprehensive prototype)

This prototype involves all LMI components (father, mother, partner,
external residences, workplace, place of education), using their
relational and exposure weights, without any specific
considerations.

**`LMI_compreh`** is used for this prototype
in the code and LMI\_C in the text.

Set up LMI\_C and standardise it.

```
# set up LMI^C
LMI_globals %<>%
  rowwise() %>%
  mutate(LMI_compreh_unscaled = sum(mother_meanLingDist*0.63506,
           father_meanLingDist*0.31703,
           LMI_partner*0.34607,
           LMI_AdditRes_total*0.03193,
           LMI_workplace*0.4728,
           LMI_eduplace*0.202255,
           na.rm = TRUE)) # we use sum because then we can skip NA's

# scale it
LMI_globals$LMI_compreh <- (LMI_globals$LMI_compreh_unscaled - mean(LMI_globals$LMI_compreh_unscaled)) / (2*sd(LMI_globals$LMI_compreh_unscaled))
```

### 2.2.4 **LMID** (age cohort-based prototype)

**`LMI_cohort`** represents the age
cohort-based prototype LMID in the code and LMI\_D in the
text.

Using age cohort-based relational weights (calculated in
**Supplementary Material Part 1**) for the agents
‘workplace’ and ‘external residence’, this prototype handles some
components of the LMI in a manner partitioned by age cohorts,
channelling in the information that age provides about the speakers.
This model attempts to take a more informed estimate depending on the
age cohort of the participants as age affects the pattern of dialect
change in the most crucial manner: the younger cohort shows much more
change, due to time elapsed since SDS.

Due to the fact that the age cohort effect is already built into the
exposure weight of the partner, the uniformly modelled relational weight
is used. As for parents, their main linguistic impact occurred in the
first few years of the speaker for both cohorts and due to the fact that
we do not have an exposure weight in this component, the uniformly
modelled relational weight is used. Workplace is treated separately, due
to the fact that for the older cohort the workplace is often missing due
to pension, and those that indicate a workplace (n=58) often do so
because they work on their own farm at the reference locality. As for
the external residence it is possible to assume that these effects are
stronger in the younger cohort due to the adolescence peak (Baxter &
Croft, 2016). However, one might also speculate that older people might
have returned a longer time ago to their reference locality (hence they
could be included in the SDATS survey) and the influence of the
additional place of residence might have (partly) faded. Also, the
experiment design suggests that participants should have grown up and
lived in the reference locality for most of their lives. Another
possibility that also decreases the effect of external residence for
older people is that they might not have lived there in their youth.

The presence of workplace and/or place of education in the younger
cohort already adds an advantage for the potential of getting a higher
LMI. Also in case of the older cohort, there is a higher chance of
having no information about a high impact long-term partner due to their
death.

First, we show a few graphs that depict the differences across the
age cohorts regarding linguistic distances of the speaker and their
workplace.

- A histogram about the linguistic distance to the workplace in the
  older cohort. It is visible that only a few speakers in the older cohort
  work in places that are linguistically different.

```
LMI_dfEnd <- read.csv("LMI_dfEnd.csv", header=T)
LMI_globals %<>%
  left_join(LMI_dfEnd %>% select(UID,workplace_meanLingDist), by="UID")

# histogram of linduistic distance and LMI_workplace of the older cohort
LMI_globals %>% filter(Age_cohort=="older") %>% ggplot(aes(x=workplace_meanLingDist)) + geom_histogram(binwidth = 0.05)
```

- A histogram about the LMI\_workplace component, only for the older
  cohort.

```
LMI_globals %>% filter(Age_cohort=="older") %>% ggplot(aes(x=LMI_workplace)) + geom_histogram(binwidth = 0.05)
```

- Scatterplots about the older cohort’s age and linguistic distance to
  workplace and LMI\_workplace, respectively, showing that it is those
  around and below the age of pension that have an LMI\_workplace over
  0.

[The code of the scatterplots are not public, for the protection of
the SDATS speakers’ anonimity]

- And a scatterplot about the younger cohort’s age and linguistic
  distance to workplace, for contrast.

### 2.2.5 Further steps in setting up the LMI prototypes

Set up LMI\_D, standardise it and plot it against age.

```
LMI_globals %<>%
  rowwise() %>%
  mutate(LMI_cohort_unscaled = sum(
           mother_meanLingDist*0.63506,
           father_meanLingDist*0.31703,
           LMI_partner*0.34607,
           ifelse(Age_cohort=="younger",LMI_AdditRes_total*0.16632,LMI_AdditRes_total*0.02056),
           ifelse(Age_cohort=="younger",LMI_workplace*0.60605,LMI_workplace*(-0.87281)),
           LMI_eduplace*0.202255,
           na.rm = TRUE)) # we use sum because then we can skip NA's

LMI_globals$LMI_cohort <- (LMI_globals$LMI_cohort_unscaled - mean(LMI_globals$LMI_cohort_unscaled)) / (2*sd(LMI_globals$LMI_cohort_unscaled))
```

Those with negative outlying values are the speakers in the older
cohort that are still working (often in a linguistically quite different
locality), and the corresponding relational weight brings down their
whole LMI\_D.

Plotting summed dialect change in the same fashion for the same
predictors.

[code for the plots not public, for the protection of the SDATS
speakers’ anonimity]

```
LMI_globals$SumChangeSDS_scaled <- (LMI_globals$sumChangeFromSDS - mean(LMI_globals$sumChangeFromSDS)) / (2*sd(LMI_globals$sumChangeFromSDS))
```

*Distribution of LMI and dialect change with
regards to age. In Panels (A)-(D) the distribution of standardised LMIA,
LMIB, LMIC and LMID values are shown (y-axes) against the age of the
speakers (x-axes). Panel (E) shows the distribution of standardised
dialect change rates, against the age of the speakers. Each point
represents a speaker (n=500). Point colour represents educational level
and shape represents sex. The blue concentrical lines show the density
of speakers*.

Export the dialect change rate and the values of the LMI prototype in
order to map it (we used the resulting table to produce the maps in
Figure 5 in the manuscript, in a separate script).

```
toexport <- LMI_globals %>% 
  select(UID, site_code, Age_cohort, LMI_minimal, LMI_compreh, LMI_cohort, sumChangeFromSDS) %>% 
  left_join(LMI_cumul %>% select(UID, LMI_cumulated), by="UID")
#write.csv(toexport, "dataForMap.csv")
```

In Section 5, you can find a table with the average dialect change
rates and the values of the four LMI prototypes in all 125 SDATS survey
localities.

Join the table of the the survey design variables and those where LMI
prototypes are set up. This table will serve as the basis for the
mixed-effects modelling.

```
compara <- LMI_globals %>%
  select(UID, Age_cohort, site_code, Sex, SumChangeSDS_scaled, LMI_minimal, LMI_compreh, LMI_cohort,
         Occupational_situation, Edu_bkgr_2cat, Aenderung_Butter:Aenderung_Schluckauf) %>%
  left_join(LMI_cumul %>%
              select(UID, LMI_cumulated),
            by="UID") %>% # reorder coloumns
  select(UID, LMI_cumulated, LMI_minimal, LMI_compreh, LMI_cohort, everything())
```

Show trends of LMI and language change against each other,
accompanied by linear and second-order polynomial regression lines.

[code for the plots not public, for the protection of the SDATS
speakers’ anonimity]

*The relation of the four LMI prototypes to
the dialect change rate. LMI values and dialect change rates are
standardised. The panels also show the numerical results of the linear
regression models. Linear (red) and second-order polynomial regress ion
lines (green) show the major trends. The slope of the lines shows the
positive correlation*

Linear regression models with LMI prototypes only. The expected
effect is linear: the higher the mobility, the higher the language
change. The regression lines show a similar, positive correlation in
case of each prototype.

**LMIA**

```
m1 <- lm(formula = SumChangeSDS_scaled ~ LMI_minimal, data=compara )
summ(m1, digits = 4)
```

|  |  |
| --- | --- |
| Observations | 500 |
| Dependent variable | SumChangeSDS\_scaled |
| Type | OLS linear regression |

|  |  |
| --- | --- |
| F(1,498) | 22.2014 |
| R² | 0.0427 |
| Adj. R² | 0.0408 |

|  | Est. | S.E. | t val. | p |
| --- | --- | --- | --- | --- |
| (Intercept) | 0.0000 | 0.0219 | 0.0000 | 1.0000 |
| LMI\_minimal | 0.2066 | 0.0438 | 4.7118 | 0.0000 |
|  |
| --- |
| Standard errors: OLS |

```
confint(m1, level=0.95)
```

```
##                   2.5 %     97.5 %
## (Intercept) -0.04302832 0.04302832
## LMI_minimal  0.12044473 0.29273038
```

```
#plot(fitted(m1),residuals(m1))
```

**LMIB**

```
m2 <- lm(formula = SumChangeSDS_scaled ~ LMI_cumulated, data=compara )
summ(m2, digits = 4)
```

|  |  |
| --- | --- |
| Observations | 500 |
| Dependent variable | SumChangeSDS\_scaled |
| Type | OLS linear regression |

|  |  |
| --- | --- |
| F(1,498) | 59.5741 |
| R² | 0.1068 |
| Adj. R² | 0.1051 |

|  | Est. | S.E. | t val. | p |
| --- | --- | --- | --- | --- |
| (Intercept) | 0.0000 | 0.0212 | 0.0000 | 1.0000 |
| LMI\_cumulated | 0.3269 | 0.0423 | 7.7184 | 0.0000 |
|  |
| --- |
| Standard errors: OLS |

```
confint(m2, level=0.95)
```

```
##                     2.5 %     97.5 %
## (Intercept)   -0.04156127 0.04156127
## LMI_cumulated  0.24366591 0.41007750
```

```
#plot(fitted(m2),residuals(m2))
```

**LMIC**

```
m3 <- lm(formula = SumChangeSDS_scaled ~ LMI_compreh, data=compara )
summ(m3, digits = 4)
```

|  |  |
| --- | --- |
| Observations | 500 |
| Dependent variable | SumChangeSDS\_scaled |
| Type | OLS linear regression |

|  |  |
| --- | --- |
| F(1,498) | 33.7612 |
| R² | 0.0635 |
| Adj. R² | 0.0616 |

|  | Est. | S.E. | t val. | p |
| --- | --- | --- | --- | --- |
| (Intercept) | 0.0000 | 0.0217 | 0.0000 | 1.0000 |
| LMI\_compreh | 0.2520 | 0.0434 | 5.8104 | 0.0000 |
|  |
| --- |
| Standard errors: OLS |

```
confint(m3, level=0.95)
```

```
##                   2.5 %     97.5 %
## (Intercept) -0.04255806 0.04255806
## LMI_compreh  0.16676963 0.33717236
```

```
#plot(fitted(m3),residuals(m3))
```

**LMID**

```
m4 <- lm(formula = SumChangeSDS_scaled ~ LMI_cohort, data=compara )
summ(m4, digits = 4)
```

|  |  |
| --- | --- |
| Observations | 500 |
| Dependent variable | SumChangeSDS\_scaled |
| Type | OLS linear regression |

|  |  |
| --- | --- |
| F(1,498) | 55.7624 |
| R² | 0.1007 |
| Adj. R² | 0.0989 |

|  | Est. | S.E. | t val. | p |
| --- | --- | --- | --- | --- |
| (Intercept) | 0.0000 | 0.0212 | 0.0000 | 1.0000 |
| LMI\_cohort | 0.3173 | 0.0425 | 7.4674 | 0.0000 |
|  |
| --- |
| Standard errors: OLS |

```
confint(m4, level=0.95)
```

```
##                   2.5 %     97.5 %
## (Intercept) -0.04170407 0.04170407
## LMI_cohort   0.23383668 0.40082002
```

```
#plot(fitted(m4),residuals(m4))
```

The effect is significant in all cases, the slopes are steeper in the
cases of LMI\_B and LMI\_D.

We put data into long format and contrast code the binary categorical
variables in a manner corresponding to the
**z-standardisation**, suggested by Gelman & Hill
(2006:55-57). If all variables are z-standardised, it will be possible
to directly compare the z-scores in the outputs of mixed-effect
regression models across LMI values and the survey design variables.

The long format, having one row that corresponds for each speaker’s
utterance of each linguistic item is needed so we can use the grouping
terms **speaker** and **item** as random
effects in the mixed models.

```
compara <- compara %>%
  pivot_longer(cols=c(Aenderung_Butter:Aenderung_Schluckauf), 
               names_to = "Item",
               values_to = "Change") %>%
  filter(!is.na(Change)) %>%
  mutate(UID = as.factor(UID),
         Edu_bkgr_2cat = as.factor(Edu_bkgr_2cat),
         Sex = as.factor(Sex),
         Age_cohort = as.factor(Age_cohort),
         Item = as.factor(Item))

# z-standardisation of the binary variables corresponding to the results of (x-mean(x))/(2*sd(x)), as if the contrast coded values had been 0 and 1 beforehand
# we need to expressly put dplyr before recode, as the 'car' package messes with this command
compara %<>%
  mutate(Edu_bkgr_2cat.z = dplyr::recode(Edu_bkgr_2cat,
                             "ohne tertiären Bildungsabschluss" = 0.5 , # we know that dialect change is higher in this group
                            "mit tertiärem Bildungsabschluss" = -0.5
                             ),
         Sex.z = dplyr::recode(Sex,
                             "M" = 0.5 , # we know that dialect change is higher in this group
                            "F" = -0.5
                             ),
         Age_cohort.z = dplyr::recode(Age_cohort,
                             "younger" = 0.5 , # we know that dialect change is higher in this group
                            "older" = -0.5
                             ))

# change item names
compara2 <- compara %>%
  mutate(Item_EN = dplyr::recode(Item,
                             "Aenderung_Butter" = "butter", 
                            "Aenderung_Kuss" = "kiss",
                            "Aenderung_Bonbon" = "candy",
                            "Aenderung_Zwiebel" = "onion",
                            "Aenderung_Wange" = "cheek",
                            "Aenderung_Schmetterling" = "butterfly",
                            "Aenderung_Pfuetze" = "puddle",
                            "Aenderung_Taschentuch" = "tissue",
                            "Aenderung_Sommersprossen" = "freckles",
                            "Aenderung_Schluckauf" = "hickup"
                             ))

levels(compara2$Item_EN) <- c("butter","butterfly","candy","cheek","freckles","hickup","kiss","onion","puddle", "tissue")

# plot change rates for the 10 lexical variables
(itemChangePlot <-   ggplot(data=compara2, aes(Item_EN, Change)) +
    geom_col(fill="gray50") +
    # ggtitle("Between-item Variation") +
    ylab("Change in speakers (max. 500)") + xlab("") +
    coord_flip() +
    # geom_text(aes(label = Change), hjust = -0.5) +
    scale_x_discrete(limits = rev(levels(compara2$Item_EN))) )
```

# 3 Preliminary tests

## 3.1 Relationship of the survey design variables and dialect change rate

Violin plots showing the correspondence across age, sex and
educational background, as binary variables to dialect change rate.

[code chunk available in the corresponding .Rmd file]

### 3.1.1 Bivariate test with the design variables as predictors

Ahead of inferential modelling, we examined the effect of the survey
design variables age cohort, sex and educational background on dialect
change using bivariate tests. The above plots shows the differences
between the distributions of the dialect change rates found in the ten
items. And below we summarise the descriptive statistics regarding the
groupwise differences.

The mean change per group and the standard deviation are as
follows.

**Age cohorts**

```
group_by(LMI_globals, Age_cohort) %>%
     summarise(
         count = n(),
         mean = mean(sumChangeFromSDS, na.rm = TRUE),
         sd = sd(sumChangeFromSDS, na.rm = TRUE)
     )
```

```
## # A tibble: 2 × 4
##   Age_cohort count  mean    sd
##   <chr>      <int> <dbl> <dbl>
## 1 older        250 0.308 0.172
## 2 younger      250 0.470 0.188
```

```
model <- lm(sumChangeFromSDS  ~ Age_cohort, data = LMI_globals)
summ(model, digits=4)
```

|  |  |
| --- | --- |
| Observations | 500 |
| Dependent variable | sumChangeFromSDS |
| Type | OLS linear regression |

|  |  |
| --- | --- |
| F(1,498) | 101.8003 |
| R² | 0.1697 |
| Adj. R² | 0.1681 |

|  | Est. | S.E. | t val. | p |
| --- | --- | --- | --- | --- |
| (Intercept) | 0.3080 | 0.0114 | 27.0691 | 0.0000 |
| Age\_cohortyounger | 0.1624 | 0.0161 | 10.0896 | 0.0000 |
|  |
| --- |
| Standard errors: OLS |

**Sex**

```
group_by(LMI_globals, Sex) %>%
     summarise(
         count = n(),
         mean = mean(sumChangeFromSDS, na.rm = TRUE),
         sd = sd(sumChangeFromSDS, na.rm = TRUE)
     )
```

```
## # A tibble: 2 × 4
##   Sex   count  mean    sd
##   <chr> <int> <dbl> <dbl>
## 1 F       250 0.371 0.191
## 2 M       250 0.408 0.202
```

```
model <- lm(sumChangeFromSDS  ~ Sex, data = LMI_globals)
summ(model, digits=4)
```

|  |  |
| --- | --- |
| Observations | 500 |
| Dependent variable | sumChangeFromSDS |
| Type | OLS linear regression |

|  |  |
| --- | --- |
| F(1,498) | 4.4557 |
| R² | 0.0089 |
| Adj. R² | 0.0069 |

|  | Est. | S.E. | t val. | p |
| --- | --- | --- | --- | --- |
| (Intercept) | 0.3706 | 0.0124 | 29.8126 | 0.0000 |
| SexM | 0.0371 | 0.0176 | 2.1108 | 0.0353 |
|  |
| --- |
| Standard errors: OLS |

**Educational background (two categories)**

```
group_by(LMI_globals, Edu_bkgr_2cat) %>%
     summarise(
         count = n(),
         mean = mean(sumChangeFromSDS, na.rm = TRUE),
         sd = sd(sumChangeFromSDS, na.rm = TRUE)
     )
```

```
## # A tibble: 2 × 4
##   Edu_bkgr_2cat                    count  mean    sd
##   <chr>                            <int> <dbl> <dbl>
## 1 mit tertiärem Bildungsabschluss    179 0.388 0.196
## 2 ohne tertiären Bildungsabschluss   321 0.390 0.198
```

```
model <- lm(sumChangeFromSDS  ~ Edu_bkgr_2cat, data = LMI_globals)
summ(model, digits=4)
```

|  |  |
| --- | --- |
| Observations | 500 |
| Dependent variable | sumChangeFromSDS |
| Type | OLS linear regression |

|  |  |
| --- | --- |
| F(1,498) | 0.0068 |
| R² | 0.0000 |
| Adj. R² | -0.0020 |

|  | Est. | S.E. | t val. | p |
| --- | --- | --- | --- | --- |
| (Intercept) | 0.3882 | 0.0148 | 26.3060 | 0.0000 |
| Edu\_bkgr\_2catohne tertiären Bildungsabschluss | 0.0015 | 0.0184 | 0.0822 | 0.9345 |
|  |
| --- |
| Standard errors: OLS |

Age cohort shows the largest difference with a *t*-test
confirming (*t* = -10.09, *p* < 0.001) that more change
is occurring in the younger cohort (*μ* = 47.03%, *SD* =
18.75%) than in the older cohort (*μ* = 30.8%, *SD* =
17.18%). The difference between genders is much smaller (*t* =
-2.11, *p* = 0.035) with slightly more change occurring among
males (*μ* = 40.77%, *SD* = 20.16%) than among females
(*μ* = 37.07%, *SD* = 19.13%). The effect of whether the
speakers’ educational background involves tertiary education, however,
is minor and not significant in the case of the test sample (*t*
= -0.082, *p* = 0.935).

For a general test of spatial patterns in change, we test whether
more urban localities show more or less dialect change. It is
operationalised by the population of the localities in 2018 - the higher
the population the more urban the perception.

```
# import the 2018 population of the survey sites
survey_sites <- read.csv("sdats_surveysites_pops.csv", header = T, stringsAsFactors = F)

LMI_globals %<>% 
  left_join(survey_sites %>% select(site_code, POPS2018), by = "site_code")

# standardise the population data
LMI_globals$POPS2018_scaled <- (LMI_globals$POPS2018 - mean(LMI_globals$POPS2018)) / (2*sd(LMI_globals$POPS2018))

model <- lm(SumChangeSDS_scaled ~ POPS2018_scaled, data = LMI_globals)
summ(model, digits=4)
```

|  |  |
| --- | --- |
| Observations | 500 |
| Dependent variable | SumChangeSDS\_scaled |
| Type | OLS linear regression |

|  |  |
| --- | --- |
| F(1,498) | 16.4824 |
| R² | 0.0320 |
| Adj. R² | 0.0301 |

|  | Est. | S.E. | t val. | p |
| --- | --- | --- | --- | --- |
| (Intercept) | 0.0000 | 0.0220 | 0.0000 | 1.0000 |
| POPS2018\_scaled | -0.1790 | 0.0441 | -4.0598 | 0.0001 |
|  |
| --- |
| Standard errors: OLS |

A graph with log population vs dialect change, coloured by age

```
ggplot(LMI_globals, aes(x=log(POPS2018),y=sumChangeFromSDS)) +
  geom_jitter(alpha=0.5) + 
  geom_smooth(method = "lm",formula = y ~ poly(x, 1), linewidth = 1, col="red") +
  geom_smooth(method = "lm",formula = y ~ poly(x, 2), linewidth = 1, col="green") +
  geom_rug(col=rgb(.5,0,0,alpha=.2)) +
  ylab("Dialect change rate") + xlab("Logarithm of the population (2018)")
```

Population shows a significant negative linear correlation with
language change, meaning that the higher the population, the less
language change will be expected. We can speculate that this can be at
least partly due to urban prestige. In the above figure we do not show
the scaled values but the actual rate of dialect change and the
logarithm of population in 2018.

Beside the above models, we use two-sampled t-tests to find out if
there is a significant difference between the dialect change of the the
groups in these (binary) variables.

```
# Shapiro-Wilk normality test for older cohort's dialect change
with(LMI_globals, shapiro.test(sumChangeFromSDS[Age_cohort == "older"])) # p = 3.544e-07
```

```
## 
##  Shapiro-Wilk normality test
## 
## data:  sumChangeFromSDS[Age_cohort == "older"]
## W = 0.95354, p-value = 3.544e-07
```

```
# Shapiro-Wilk normality test for younger cohort's dialect change
with(LMI_globals, shapiro.test(sumChangeFromSDS[Age_cohort == "younger"])) # p = 4.387e-05
```

```
## 
##  Shapiro-Wilk normality test
## 
## data:  sumChangeFromSDS[Age_cohort == "younger"]
## W = 0.97024, p-value = 4.387e-05
```

```
hist(LMI_globals$sumChangeFromSDS[LMI_globals$Age_cohort == "older"])
```

```
hist(LMI_globals$sumChangeFromSDS[LMI_globals$Age_cohort == "younger"])
```

```
# Do the two populations have the same variances? We’ll use F-test to test for homogeneity in variances.
 var.test(sumChangeFromSDS ~ Age_cohort, data = LMI_globals)
```

```
## 
##  F test to compare two variances
## 
## data:  sumChangeFromSDS by Age_cohort
## F = 0.83961, num df = 249, denom df = 249, p-value = 0.1685
## alternative hypothesis: true ratio of variances is not equal to 1
## 95 percent confidence interval:
##  0.6545537 1.0769881
## sample estimates:
## ratio of variances 
##          0.8396109
```

```
 # Question : Is there any significant difference between the older and and younger cohort's language change?
t.test(sumChangeFromSDS ~ Age_cohort, data = LMI_globals, var.equal = TRUE)
```

```
## 
##  Two Sample t-test
## 
## data:  sumChangeFromSDS by Age_cohort
## t = -10.09, df = 498, p-value < 2.2e-16
## alternative hypothesis: true difference in means between group older and group younger is not equal to 0
## 95 percent confidence interval:
##  -0.1939709 -0.1307402
## sample estimates:
##   mean in group older mean in group younger 
##             0.3080000             0.4703556
```

**Shapiro-Wilk normality test**  
The two p-values are smaller than the significance level 0.05 implying
that the distribution of the data are significantly different from the
normal distribution. In other words, we can NOT assume the
normality.

**F-test for the homogeneity of variances**  
The p-value of F-test is p = 0.1685. It’s greater than the significance
level alpha = 0.05. In conclusion, there is no significant difference
between the variances of the two sets of data. Therefore, we can use the
classic t-test which assumes the equality of the two variances.

The p-value of the test is < 2.2e-16, which is less than the
significance level alpha = 0.05. We can conclude that older cohort’s
average language change is significantly different from younger cohort’s
average language change with a p-value <0.001.

The same calculations for gender:

```
# Shapiro-Wilk normality test for males' and females' dialect change
with(LMI_globals, shapiro.test(sumChangeFromSDS[Sex == "M"])) # p = 4.193e-05
```

```
## 
##  Shapiro-Wilk normality test
## 
## data:  sumChangeFromSDS[Sex == "M"]
## W = 0.9701, p-value = 4.193e-05
```

```
with(LMI_globals, shapiro.test(sumChangeFromSDS[Sex == "F"])) # p = 7.104e-06
```

```
## 
##  Shapiro-Wilk normality test
## 
## data:  sumChangeFromSDS[Sex == "F"]
## W = 0.96438, p-value = 7.104e-06
```

```
#  we can NOT assume the normality

#F-test to test for homogeneity in variances
var.test(sumChangeFromSDS ~ Sex, data = LMI_globals)
```

```
## 
##  F test to compare two variances
## 
## data:  sumChangeFromSDS by Sex
## F = 0.89976, num df = 249, denom df = 249, p-value = 0.4052
## alternative hypothesis: true ratio of variances is not equal to 1
## 95 percent confidence interval:
##  0.7014454 1.1541427
## sample estimates:
## ratio of variances 
##            0.89976
```

```
#there is no significant difference between the variances of the two sets of data

t.test(sumChangeFromSDS ~ Sex, data = LMI_globals, var.equal = TRUE)
```

```
## 
##  Two Sample t-test
## 
## data:  sumChangeFromSDS by Sex
## t = -2.1108, df = 498, p-value = 0.03528
## alternative hypothesis: true difference in means between group F and group M is not equal to 0
## 95 percent confidence interval:
##  -0.071653466 -0.002568756
## sample estimates:
## mean in group F mean in group M 
##       0.3706222       0.4077333
```

The p-value of the t-test is 0.03528, which is less than the
significance level alpha = 0.05. We can conclude that males’ average
dialect change is significantly larger from females’ average language
change.

The same calculations for education background:

```
# it looks very similar, also the groups are not of equal size

# we only perform the F-test
var.test(sumChangeFromSDS ~ Edu_bkgr_2cat, data = LMI_globals)
```

```
## 
##  F test to compare two variances
## 
## data:  sumChangeFromSDS by Edu_bkgr_2cat
## F = 0.97834, num df = 178, denom df = 320, p-value = 0.8787
## alternative hypothesis: true ratio of variances is not equal to 1
## 95 percent confidence interval:
##  0.7581454 1.2748645
## sample estimates:
## ratio of variances 
##          0.9783428
```

```
#there is no significant difference between the variances of the two sets of data

t.test(sumChangeFromSDS ~ Edu_bkgr_2cat, data = LMI_globals, var.equal = TRUE)
```

```
## 
##  Two Sample t-test
## 
## data:  sumChangeFromSDS by Edu_bkgr_2cat
## t = -0.082178, df = 498, p-value = 0.9345
## alternative hypothesis: true difference in means between group mit tertiärem Bildungsabschluss and group ohne tertiären Bildungsabschluss is not equal to 0
## 95 percent confidence interval:
##  -0.03769984  0.03467276
## sample estimates:
##  mean in group mit tertiärem Bildungsabschluss 
##                                      0.3882061 
## mean in group ohne tertiären Bildungsabschluss 
##                                      0.3897196
```

The p-value of the t-test is 0.9345, which is larger than the
significance level alpha = 0.05. We can conclude that the average
dialect change of people without tertiary education is not significantly
different from the average dialect change of people with tertiary
education.

**Urbanity**

We also test the effect of urbanity. In Switzerland, communities over
10,000 inhabitants qualify for the right to be called ‘cities’, thus we
compare those SDATS localities over (nr of speakers = 160) and under
10,000 (nr of speakers = 340) with regards to dialect change rate.

```
urbtest <- LMI_globals %>% 
  mutate(POPS2018Urb = ifelse(POPS2018>10000,"urban","non-urban"))
group_by(urbtest, POPS2018Urb) %>%
     summarise(
         count = n(),
         mean = mean(sumChangeFromSDS, na.rm = TRUE),
         sd = sd(sumChangeFromSDS, na.rm = TRUE)
     )
```

```
## # A tibble: 2 × 4
##   POPS2018Urb count  mean    sd
##   <chr>       <int> <dbl> <dbl>
## 1 non-urban     340 0.401 0.191
## 2 urban         160 0.365 0.208
```

```
# Shapiro-Wilk normality test for urbans' and non-urban' dialect change
with(urbtest, shapiro.test(sumChangeFromSDS[POPS2018Urb == "urban"])) # p = 4.193e-05
```

```
## 
##  Shapiro-Wilk normality test
## 
## data:  sumChangeFromSDS[POPS2018Urb == "urban"]
## W = 0.96413, p-value = 0.0003678
```

```
with(urbtest, shapiro.test(sumChangeFromSDS[POPS2018Urb == "non-urban"])) # p = 7.104e-06
```

```
## 
##  Shapiro-Wilk normality test
## 
## data:  sumChangeFromSDS[POPS2018Urb == "non-urban"]
## W = 0.96639, p-value = 4.524e-07
```

```
#  we can NOT assume the normality

#F-test to test for homogeneity in variances
var.test(sumChangeFromSDS ~ POPS2018Urb, data = urbtest)
```

```
## 
##  F test to compare two variances
## 
## data:  sumChangeFromSDS by POPS2018Urb
## F = 0.84656, num df = 339, denom df = 159, p-value = 0.2105
## alternative hypothesis: true ratio of variances is not equal to 1
## 95 percent confidence interval:
##  0.6438421 1.0986617
## sample estimates:
## ratio of variances 
##          0.8465613
```

```
#there is no significant difference between the variances of the two sets of data

t.test(sumChangeFromSDS ~ POPS2018Urb, data = urbtest, var.equal = TRUE)
```

```
## 
##  Two Sample t-test
## 
## data:  sumChangeFromSDS by POPS2018Urb
## t = 1.9069, df = 498, p-value = 0.05711
## alternative hypothesis: true difference in means between group non-urban and group urban is not equal to 0
## 95 percent confidence interval:
##  -0.001090957  0.073019062
## sample estimates:
## mean in group non-urban     mean in group urban 
##               0.4006863               0.3647222
```

The two p-values are smaller than the significance level 0.05
implying that the distribution of the data are significantly different
from the normal distribution. In other words, we can NOT assume the
normality.

The p-value of F-test is greater than the significance level alpha =
0.05. In conclusion, there is no significant difference between the
variances of the two sets of data.

The t-test shows (p-value is almost significant) that the dialect
change in the non-urban areas is almost significantly higher than in the
urban areas. It also depends, however on how the line is drawn, as the
largest urban places change remarkably less than the rural areas.

```
ggplot(urbtest, aes(x=POPS2018Urb, y=sumChangeFromSDS)) +
  geom_violin(aes(fill = POPS2018Urb)) +
  geom_boxplot(width=0.1)+
  scale_fill_manual(values = c("yellow","dodgerblue2")) + 
  ylab("Aggregate dialect change rate") +
  xlab("Urbanity") +
  scale_x_discrete(labels=c("below 10,000 inhabitants","over 10,000 inhabitants"))+
  theme(legend.position = "none")
```

### 3.1.2 Kruskal-Wallis test

We use the Kruskal-Wallis test for finding out if the LMI prototypes’
values and dialect change rate are different across the 125
localities.

Kruskal-Wallis test by rank is a non-parametric alternative to
one-way ANOVA test, which extends the two-samples Wilcoxon test in the
situation where there are more than two groups. Its use is recommended
when the assumptions of one-way ANOVA test are not met.

We perform this test because the distributions within localities
cannot be assumed to be normal as there are only four speakers per
locality.

We perform the test on the four LMI prototypes

```
# site_code = reference locality (n=125)

kruskal.test(LMI_minimal ~ site_code, data = LMI_globals) # p>0.05!
```

```
## 
##  Kruskal-Wallis rank sum test
## 
## data:  LMI_minimal by site_code
## Kruskal-Wallis chi-squared = 141.17, df = 124, p-value = 0.1388
```

```
kruskal.test(LMI_cumulated ~ site_code, data = LMI_cumul) # p<0.05
```

```
## 
##  Kruskal-Wallis rank sum test
## 
## data:  LMI_cumulated by site_code
## Kruskal-Wallis chi-squared = 155.14, df = 124, p-value = 0.03046
```

```
kruskal.test(LMI_compreh ~ site_code, data = LMI_globals) # p<0.05
```

```
## 
##  Kruskal-Wallis rank sum test
## 
## data:  LMI_compreh by site_code
## Kruskal-Wallis chi-squared = 152.06, df = 124, p-value = 0.04417
```

```
kruskal.test(LMI_cohort ~ site_code, data = LMI_globals) # p<0.05
```

```
## 
##  Kruskal-Wallis rank sum test
## 
## data:  LMI_cohort by site_code
## Kruskal-Wallis chi-squared = 153.02, df = 124, p-value = 0.03941
```

And also on the sum of dialect change.

```
kruskal.test(sumChangeFromSDS ~ site_code, data = LMI_globals)  # p<0.05
```

```
## 
##  Kruskal-Wallis rank sum test
## 
## data:  sumChangeFromSDS by site_code
## Kruskal-Wallis chi-squared = 266.82, df = 124, p-value = 1.789e-12
```

The Kruskal-Wallis test shows a significant difference across the
reference localities for the rate of dialect change and for each LMI
prototype except for the ‘minimal prototype’ LMI\_A. Rates of dialect
change are also mapped at each reference locality in Figure 5 in the
manuscript, separately for the two cohorts.

### 3.1.3 Spatial autocorrelation

Using Moran’s *I*, we would like to find out if the dialectal
change rate and the values of the LMI prototypes in space are
autocorrelated, thus whether clusters with similar values are present,
or the spatial distribution is random.

**Dialect change**

```
library(ape)
library(fields)

# put together the test table for spatial autocorrelation, including the spatial coordinates
df_forSA <- LMI_globals %>% select(UID, site_code, LMI_minimal, LMI_compreh, LMI_cohort, SumChangeSDS_scaled) %>% 
  left_join(LMI_cumul %>%  select(UID,LMI_cumulated), by="UID") %>% 
  left_join(survey_sites %>% select(site_code, LAT, LONG), by="site_code")

# create a distance matrix
sdats_distMat <-round(RdistEarth(x1=as.matrix(df_forSA %>% select(LONG, LAT)), miles=FALSE),4)

# Moran's I calculations

# dialect change
Moran.I(df_forSA$SumChangeSDS_scaled, sdats_distMat)
```

```
## $observed
## [1] -0.02229374
## 
## $expected
## [1] -0.002004008
## 
## $sd
## [1] 0.001205752
## 
## $p.value
## [1] 1.53575e-63
```

**LMIA**

```
# Minimal LMI prototype
Moran.I(df_forSA$LMI_minimal, sdats_distMat)
```

```
## $observed
## [1] -0.004016714
## 
## $expected
## [1] -0.002004008
## 
## $sd
## [1] 0.001204088
## 
## $p.value
## [1] 0.09461103
```

**LMIB**

```
# Cumulated LMI prototype
Moran.I(df_forSA$LMI_cumulated, sdats_distMat)
```

```
## $observed
## [1] -0.004998405
## 
## $expected
## [1] -0.002004008
## 
## $sd
## [1] 0.001203484
## 
## $p.value
## [1] 0.01284251
```

**LMIC**

```
# comprehensive LMI prototype
Moran.I(df_forSA$LMI_compreh, sdats_distMat)
```

```
## $observed
## [1] -0.005264304
## 
## $expected
## [1] -0.002004008
## 
## $sd
## [1] 0.001203989
## 
## $p.value
## [1] 0.00677082
```

**LMID**

```
# Cohorts LMI prototype
Moran.I(df_forSA$LMI_cohort, sdats_distMat)
```

```
## $observed
## [1] -0.005524196
## 
## $expected
## [1] -0.002004008
## 
## $sd
## [1] 0.001203195
## 
## $p.value
## [1] 0.003436827
```

Moran’s *I* analysis shows negative spatial autocorrelation in
dialect change and for each LMI prototype. This is significant except
for LMI\_A. These mean that there is a higher chance to find different
dialect change rates in nearby localities rather than finding similar
ones. That is, localities with similar dialect change or LMI values do
not cluster in space significantly, but the distribution of the values
in space is also not random.

# 4 Mixed-effects models

We test the performance of the four LMI prototypes values in
mixed-effects models.

Models with growing numbers of predictors are set up in order to
verify that the fully adjusted model (henceforth *full model*) is
a valid test our hypotheses. In the paper, however, only the full model
is reported. We also argue that we use the survey design variables as
control variables (fixed effects) because they are indeed the ones based
on which we selected our speakers, thus they are valid grouping
factors.

Please, click here to jump to the Full
models.

The following models are set up:

- **“Base” models**
  - Fixed effects: LMI prototypes
  - Random effects: UID and Item
- **“Reduced” models**
  - Fixed effects: LMI prototypes, Age cohorts (the most important
    survey design variable)
  - Random effects: UID and Item
- **Fully adjusted model**
  - Fixed effects: LMI prototypes, Age cohorts, Sex and Educational
    background
  - Random effects: UID and Item

## 4.1 Base model

**Intercept-only random effect model**

```
null.base <- glmer(Change ~1 + (1|UID) + (1|Item),
                        data=compara, family=binomial(), 
                  control = glmerControl(optimizer = "bobyqa"))
summ(null.base, digits =4)
```

|  |  |
| --- | --- |
| Observations | 4983 |
| Dependent variable | Change |
| Type | Mixed effects generalized linear model |
| Family | binomial |
| Link | logit |

|  |  |
| --- | --- |
| AIC | 6242.7146 |
| BIC | 6262.2560 |
| Pseudo-R² (fixed effects) | 0.0000 |
| Pseudo-R² (total) | 0.1952 |

| Fixed Effects | | | | |
| --- | --- | --- | --- | --- |
|  | Est. | S.E. | z val. | p |
| (Intercept) | -0.5103 | 0.1985 | -2.5715 | 0.0101 |

| Random Effects | | |
| --- | --- | --- |
| Group | Parameter | Std. Dev. |
| UID | (Intercept) | 0.6501 |
| Item | (Intercept) | 0.6126 |

| Grouping Variables | | |
| --- | --- | --- |
| Group | # groups | ICC |
| UID | 500 | 0.1034 |
| Item | 10 | 0.0918 |

**LMIA**

```
minim.base <- glmer(Change ~ LMI_minimal + (1|UID) + (1|Item),
                        data=compara, family=binomial(),
                  control = glmerControl(optimizer = "bobyqa"))
summ(minim.base, digits =4)
```

|  |  |
| --- | --- |
| Observations | 4983 |
| Dependent variable | Change |
| Type | Mixed effects generalized linear model |
| Family | binomial |
| Link | logit |

|  |  |
| --- | --- |
| AIC | 6222.5105 |
| BIC | 6248.5657 |
| Pseudo-R² (fixed effects) | 0.0096 |
| Pseudo-R² (total) | 0.1952 |

| Fixed Effects | | | | |
| --- | --- | --- | --- | --- |
|  | Est. | S.E. | z val. | p |
| (Intercept) | -0.5102 | 0.1983 | -2.5731 | 0.0101 |
| LMI\_minimal | 0.3957 | 0.0831 | 4.7613 | 0.0000 |

| Random Effects | | |
| --- | --- | --- |
| Group | Parameter | Std. Dev. |
| UID | (Intercept) | 0.6194 |
| Item | (Intercept) | 0.6126 |

| Grouping Variables | | |
| --- | --- | --- |
| Group | # groups | ICC |
| UID | 500 | 0.0948 |
| Item | 10 | 0.0927 |

```
# highly significant

effects.minim.base<- allEffects(minim.base)

plot(effects.minim.base, 'LMI_minimal', xlab="Only LMI^A + UID and Item as random effects", main="LMI^A Effect", symbols=FALSE)
```

**LMIB**

```
cumul.base <- glmer(Change ~ LMI_cumulated + (1|UID) + (1|Item),
                        data=compara, family=binomial(),
                  control = glmerControl(optimizer = "bobyqa"))
summ(cumul.base, digits =4)
```

|  |  |
| --- | --- |
| Observations | 4983 |
| Dependent variable | Change |
| Type | Mixed effects generalized linear model |
| Family | binomial |
| Link | logit |

|  |  |
| --- | --- |
| AIC | 6187.9523 |
| BIC | 6214.0075 |
| Pseudo-R² (fixed effects) | 0.0236 |
| Pseudo-R² (total) | 0.1952 |

| Fixed Effects | | | | |
| --- | --- | --- | --- | --- |
|  | Est. | S.E. | z val. | p |
| (Intercept) | -0.5091 | 0.1980 | -2.5719 | 0.0101 |
| LMI\_cumulated | 0.6219 | 0.0811 | 7.6721 | 0.0000 |

| Random Effects | | |
| --- | --- | --- |
| Group | Parameter | Std. Dev. |
| UID | (Intercept) | 0.5711 |
| Item | (Intercept) | 0.6125 |

| Grouping Variables | | |
| --- | --- | --- |
| Group | # groups | ICC |
| UID | 500 | 0.0817 |
| Item | 10 | 0.0940 |

```
# highly significant

effects.cumul.base<- allEffects(cumul.base)

plot(effects.cumul.base, 'LMI_cumulated', xlab="Only LMI^B + UID and Item as random", main="LMI^B Effect", symbols=FALSE)
```

**LMIC**

```
compreh.base <- glmer(Change ~ LMI_compreh + (1|UID) + (1|Item),
                        data=compara, family=binomial(),
                  control = glmerControl(optimizer = "bobyqa"))
summ(compreh.base, digits =4)
```

|  |  |
| --- | --- |
| Observations | 4983 |
| Dependent variable | Change |
| Type | Mixed effects generalized linear model |
| Family | binomial |
| Link | logit |

|  |  |
| --- | --- |
| AIC | 6211.3998 |
| BIC | 6237.4550 |
| Pseudo-R² (fixed effects) | 0.0142 |
| Pseudo-R² (total) | 0.1953 |

| Fixed Effects | | | | |
| --- | --- | --- | --- | --- |
|  | Est. | S.E. | z val. | p |
| (Intercept) | -0.5100 | 0.1982 | -2.5734 | 0.0101 |
| LMI\_compreh | 0.4825 | 0.0825 | 5.8449 | 0.0000 |

| Random Effects | | |
| --- | --- | --- |
| Group | Parameter | Std. Dev. |
| UID | (Intercept) | 0.6042 |
| Item | (Intercept) | 0.6126 |

| Grouping Variables | | |
| --- | --- | --- |
| Group | # groups | ICC |
| UID | 500 | 0.0906 |
| Item | 10 | 0.0931 |

```
# highly significant

effects.compreh.base<- allEffects(compreh.base)

plot(effects.compreh.base, 'LMI_compreh', xlab="Only LMI^C + UID and Item as random", main="LMI^C Effect", symbols=FALSE)
```

**LMID**

```
cohort.base <- glmer(Change ~ LMI_cohort + (1|UID) + (1|Item),
                        data=compara, family=binomial(),
                  control = glmerControl(optimizer = "bobyqa"))
summ(cohort.base, digits =4)
```

|  |  |
| --- | --- |
| Observations | 4983 |
| Dependent variable | Change |
| Type | Mixed effects generalized linear model |
| Family | binomial |
| Link | logit |

|  |  |
| --- | --- |
| AIC | 6190.9505 |
| BIC | 6217.0056 |
| Pseudo-R² (fixed effects) | 0.0226 |
| Pseudo-R² (total) | 0.1954 |

| Fixed Effects | | | | |
| --- | --- | --- | --- | --- |
|  | Est. | S.E. | z val. | p |
| (Intercept) | -0.5095 | 0.1980 | -2.5733 | 0.0101 |
| LMI\_cohort | 0.6075 | 0.0815 | 7.4553 | 0.0000 |

| Random Effects | | |
| --- | --- | --- |
| Group | Parameter | Std. Dev. |
| UID | (Intercept) | 0.5756 |
| Item | (Intercept) | 0.6126 |

| Grouping Variables | | |
| --- | --- | --- |
| Group | # groups | ICC |
| UID | 500 | 0.0829 |
| Item | 10 | 0.0939 |

```
# highly significant


effects.cohort.base<- allEffects(cohort.base)


plot(effects.cohort.base, 'LMI_cohort', xlab="Only LMI^D + UID and Item as random", main="LMI^D Effect", symbols=FALSE)
```

All LMI prototypes show a significant effect.

For example, the exponentiated slope estimate of LMI\_D,
`exp(0.6075)= 1.835836` means that increasing the
standardised LMI value by two standard deviations increases the odds of
dialect change by 83% in an average speaker, in one item. *SE*
values stay similar across the four prototypes.

The below graph shows the comparability of the four prototypes and
their individual effect on the dialect change rate.

```
library("PerformanceAnalytics") # for correlation plot and changes anova too

# Plotting the LMI prototypes against the dialect changes too

df_forCP <- LMI_globals %>% select(UID, sumChangeFromSDS, LMI_minimal, LMI_compreh, LMI_cohort) %>%
  left_join(LMI_cumul %>% select(UID,LMI_cumulated)) %>% select(-UID)
chart.Correlation(df_forCP, histogram=TRUE, pch=19)
```

## 4.2 Reduced models

Having observed that age is a crucial predictor of dialect change,
the effect of age cohorts was tested alongside the LMI.

**LMIA**

```
minimmodel.age <- glmer(Change ~ LMI_minimal +  Age_cohort.z +(1|UID) + (1|Item),
                        data=compara, family=binomial(), na.action = na.fail,
                  control = glmerControl(optimizer = "bobyqa"))
summ(minimmodel.age, digits=4)
```

|  |  |
| --- | --- |
| Observations | 4983 |
| Dependent variable | Change |
| Type | Mixed effects generalized linear model |
| Family | binomial |
| Link | logit |

|  |  |
| --- | --- |
| AIC | 6132.7231 |
| BIC | 6165.2920 |
| Pseudo-R² (fixed effects) | 0.0452 |
| Pseudo-R² (total) | 0.1960 |

| Fixed Effects | | | | |
| --- | --- | --- | --- | --- |
|  | Est. | S.E. | z val. | p |
| (Intercept) | -0.5108 | 0.1977 | -2.5836 | 0.0098 |
| LMI\_minimal | 0.3469 | 0.0760 | 4.5639 | 0.0000 |
| Age\_cohort.z | 0.7645 | 0.0775 | 9.8696 | 0.0000 |

| Random Effects | | |
| --- | --- | --- |
| Group | Parameter | Std. Dev. |
| UID | (Intercept) | 0.4913 |
| Item | (Intercept) | 0.6131 |

| Grouping Variables | | |
| --- | --- | --- |
| Group | # groups | ICC |
| UID | 500 | 0.0618 |
| Item | 10 | 0.0962 |

```
# highly significant, age too
```

**LMIB**

```
cumulmodel.age <- glmer(Change ~ LMI_cumulated +  Age_cohort.z + (1|UID) + (1|Item),
                        data=compara, family=binomial(), na.action = na.fail,
                  control = glmerControl(optimizer = "bobyqa"))
summ(cumulmodel.age, digits=4)
```

|  |  |
| --- | --- |
| Observations | 4983 |
| Dependent variable | Change |
| Type | Mixed effects generalized linear model |
| Family | binomial |
| Link | logit |

|  |  |
| --- | --- |
| AIC | 6123.4781 |
| BIC | 6156.0470 |
| Pseudo-R² (fixed effects) | 0.0483 |
| Pseudo-R² (total) | 0.1959 |

| Fixed Effects | | | | |
| --- | --- | --- | --- | --- |
|  | Est. | S.E. | z val. | p |
| (Intercept) | -0.5100 | 0.1976 | -2.5809 | 0.0099 |
| LMI\_cumulated | 0.4333 | 0.0787 | 5.5043 | 0.0000 |
| Age\_cohort.z | 0.6623 | 0.0794 | 8.3396 | 0.0000 |

| Random Effects | | |
| --- | --- | --- |
| Group | Parameter | Std. Dev. |
| UID | (Intercept) | 0.4778 |
| Item | (Intercept) | 0.6130 |

| Grouping Variables | | |
| --- | --- | --- |
| Group | # groups | ICC |
| UID | 500 | 0.0586 |
| Item | 10 | 0.0965 |

```
# highly significant, age too
```

**LMIC**

```
comprehmodel.age <- glmer(Change ~ LMI_compreh +  Age_cohort.z + (1|UID) + (1|Item),
                        data=compara, family=binomial(), na.action = na.fail,
                  control = glmerControl(optimizer = "bobyqa"))
summ(comprehmodel.age, digits=4)
```

|  |  |
| --- | --- |
| Observations | 4983 |
| Dependent variable | Change |
| Type | Mixed effects generalized linear model |
| Family | binomial |
| Link | logit |

|  |  |
| --- | --- |
| AIC | 6122.7829 |
| BIC | 6155.3518 |
| Pseudo-R² (fixed effects) | 0.0486 |
| Pseudo-R² (total) | 0.1961 |

| Fixed Effects | | | | |
| --- | --- | --- | --- | --- |
|  | Est. | S.E. | z val. | p |
| (Intercept) | -0.5105 | 0.1976 | -2.5828 | 0.0098 |
| LMI\_compreh | 0.4223 | 0.0758 | 5.5737 | 0.0000 |
| Age\_cohort.z | 0.7527 | 0.0768 | 9.8048 | 0.0000 |

| Random Effects | | |
| --- | --- | --- |
| Group | Parameter | Std. Dev. |
| UID | (Intercept) | 0.4770 |
| Item | (Intercept) | 0.6131 |

| Grouping Variables | | |
| --- | --- | --- |
| Group | # groups | ICC |
| UID | 500 | 0.0584 |
| Item | 10 | 0.0965 |

```
# highly significant, age too
```

**LMID**

```
cohortmodel.age <- glmer(Change ~ LMI_cohort +  Age_cohort.z + (1|UID) + (1|Item),
                        data=compara, family=binomial(), na.action = na.fail,
                  control = glmerControl(optimizer = "bobyqa"))
summ(cohortmodel.age, digits=4)
```

|  |  |
| --- | --- |
| Observations | 4983 |
| Dependent variable | Change |
| Type | Mixed effects generalized linear model |
| Family | binomial |
| Link | logit |

|  |  |
| --- | --- |
| AIC | 6118.7472 |
| BIC | 6151.3162 |
| Pseudo-R² (fixed effects) | 0.0501 |
| Pseudo-R² (total) | 0.1961 |

| Fixed Effects | | | | |
| --- | --- | --- | --- | --- |
|  | Est. | S.E. | z val. | p |
| (Intercept) | -0.5102 | 0.1976 | -2.5820 | 0.0098 |
| LMI\_cohort | 0.4590 | 0.0774 | 5.9305 | 0.0000 |
| Age\_cohort.z | 0.6866 | 0.0777 | 8.8326 | 0.0000 |

| Random Effects | | |
| --- | --- | --- |
| Group | Parameter | Std. Dev. |
| UID | (Intercept) | 0.4708 |
| Item | (Intercept) | 0.6130 |

| Grouping Variables | | |
| --- | --- | --- |
| Group | # groups | ICC |
| UID | 500 | 0.0570 |
| Item | 10 | 0.0967 |

```
# highly significant, age too
```

In all models now, both LMI and age are very highly significant.

Effect plots:

```
plot(allEffects(minimmodel.age))
```

```
plot(allEffects(cumulmodel.age))
```

```
plot(allEffects(comprehmodel.age))
```

```
plot(allEffects(cohortmodel.age))
```

In models including age cohort alongside the LMI as fixed effects,
both terms appear highly significant for each LMI prototype, while
*SE* values remain low. Also, both terms prove to be a meaningful
addition to a model containing the other term as a sole fixed effect, as
shown by *χ2*-tests (see below).

These tests check if the addition of age cohort has a significant
effect and check if the difference between the models now grew.

```
anova(minim.base, minimmodel.age)
```

```
## Data: compara
## Models:
## minim.base: Change ~ LMI_minimal + (1 | UID) + (1 | Item)
## minimmodel.age: Change ~ LMI_minimal + Age_cohort.z + (1 | UID) + (1 | Item)
##                npar    AIC    BIC  logLik deviance  Chisq Df Pr(>Chisq)    
## minim.base        4 6222.5 6248.6 -3107.3   6214.5                         
## minimmodel.age    5 6132.7 6165.3 -3061.4   6122.7 91.787  1  < 2.2e-16 ***
## ---
## Signif. codes:  0 '***' 0.001 '**' 0.01 '*' 0.05 '.' 0.1 ' ' 1
```

```
anova(cumul.base, cumulmodel.age)
```

```
## Data: compara
## Models:
## cumul.base: Change ~ LMI_cumulated + (1 | UID) + (1 | Item)
## cumulmodel.age: Change ~ LMI_cumulated + Age_cohort.z + (1 | UID) + (1 | Item)
##                npar    AIC  BIC  logLik deviance  Chisq Df Pr(>Chisq)    
## cumul.base        4 6188.0 6214 -3090.0   6180.0                         
## cumulmodel.age    5 6123.5 6156 -3056.7   6113.5 66.474  1  3.545e-16 ***
## ---
## Signif. codes:  0 '***' 0.001 '**' 0.01 '*' 0.05 '.' 0.1 ' ' 1
```

```
anova(compreh.base, comprehmodel.age)
```

```
## Data: compara
## Models:
## compreh.base: Change ~ LMI_compreh + (1 | UID) + (1 | Item)
## comprehmodel.age: Change ~ LMI_compreh + Age_cohort.z + (1 | UID) + (1 | Item)
##                  npar    AIC    BIC  logLik deviance  Chisq Df Pr(>Chisq)    
## compreh.base        4 6211.4 6237.5 -3101.7   6203.4                         
## comprehmodel.age    5 6122.8 6155.4 -3056.4   6112.8 90.617  1  < 2.2e-16 ***
## ---
## Signif. codes:  0 '***' 0.001 '**' 0.01 '*' 0.05 '.' 0.1 ' ' 1
```

```
anova(cohort.base, cohortmodel.age)
```

```
## Data: compara
## Models:
## cohort.base: Change ~ LMI_cohort + (1 | UID) + (1 | Item)
## cohortmodel.age: Change ~ LMI_cohort + Age_cohort.z + (1 | UID) + (1 | Item)
##                 npar    AIC    BIC  logLik deviance  Chisq Df Pr(>Chisq)    
## cohort.base        4 6191.0 6217.0 -3091.5   6183.0                         
## cohortmodel.age    5 6118.7 6151.3 -3054.4   6108.7 74.203  1  < 2.2e-16 ***
## ---
## Signif. codes:  0 '***' 0.001 '**' 0.01 '*' 0.05 '.' 0.1 ' ' 1
```

For each LMI, adding age cohorts means a highly significant
improvement.

Let’s test if the LMI prototypes make a significant difference when
age cohort is already in the model.

```
model.age <- glmer(Change ~ Age_cohort.z + (1|UID) + (1|Item),
                        data=compara, family=binomial(),
                  control = glmerControl(optimizer = "bobyqa"))
summ(model.age, digits=4)
```

|  |  |
| --- | --- |
| Observations | 4983 |
| Dependent variable | Change |
| Type | Mixed effects generalized linear model |
| Family | binomial |
| Link | logit |

|  |  |
| --- | --- |
| AIC | 6151.1552 |
| BIC | 6177.2103 |
| Pseudo-R² (fixed effects) | 0.0378 |
| Pseudo-R² (total) | 0.1958 |

| Fixed Effects | | | | |
| --- | --- | --- | --- | --- |
|  | Est. | S.E. | z val. | p |
| (Intercept) | -0.5106 | 0.1978 | -2.5817 | 0.0098 |
| Age\_cohort.z | 0.7865 | 0.0789 | 9.9723 | 0.0000 |

| Random Effects | | |
| --- | --- | --- |
| Group | Parameter | Std. Dev. |
| UID | (Intercept) | 0.5201 |
| Item | (Intercept) | 0.6129 |

| Grouping Variables | | |
| --- | --- | --- |
| Group | # groups | ICC |
| UID | 500 | 0.0687 |
| Item | 10 | 0.0954 |

```
anova(model.age, minimmodel.age)
```

```
## Data: compara
## Models:
## model.age: Change ~ Age_cohort.z + (1 | UID) + (1 | Item)
## minimmodel.age: Change ~ LMI_minimal + Age_cohort.z + (1 | UID) + (1 | Item)
##                npar    AIC    BIC  logLik deviance  Chisq Df Pr(>Chisq)    
## model.age         4 6151.2 6177.2 -3071.6   6143.2                         
## minimmodel.age    5 6132.7 6165.3 -3061.4   6122.7 20.432  1  6.178e-06 ***
## ---
## Signif. codes:  0 '***' 0.001 '**' 0.01 '*' 0.05 '.' 0.1 ' ' 1
```

```
anova(model.age, cumulmodel.age)
```

```
## Data: compara
## Models:
## model.age: Change ~ Age_cohort.z + (1 | UID) + (1 | Item)
## cumulmodel.age: Change ~ LMI_cumulated + Age_cohort.z + (1 | UID) + (1 | Item)
##                npar    AIC    BIC  logLik deviance  Chisq Df Pr(>Chisq)    
## model.age         4 6151.2 6177.2 -3071.6   6143.2                         
## cumulmodel.age    5 6123.5 6156.0 -3056.7   6113.5 29.677  1  5.103e-08 ***
## ---
## Signif. codes:  0 '***' 0.001 '**' 0.01 '*' 0.05 '.' 0.1 ' ' 1
```

```
anova(model.age, comprehmodel.age)
```

```
## Data: compara
## Models:
## model.age: Change ~ Age_cohort.z + (1 | UID) + (1 | Item)
## comprehmodel.age: Change ~ LMI_compreh + Age_cohort.z + (1 | UID) + (1 | Item)
##                  npar    AIC    BIC  logLik deviance  Chisq Df Pr(>Chisq)    
## model.age           4 6151.2 6177.2 -3071.6   6143.2                         
## comprehmodel.age    5 6122.8 6155.4 -3056.4   6112.8 30.372  1  3.566e-08 ***
## ---
## Signif. codes:  0 '***' 0.001 '**' 0.01 '*' 0.05 '.' 0.1 ' ' 1
```

```
anova(model.age, cohortmodel.age)
```

```
## Data: compara
## Models:
## model.age: Change ~ Age_cohort.z + (1 | UID) + (1 | Item)
## cohortmodel.age: Change ~ LMI_cohort + Age_cohort.z + (1 | UID) + (1 | Item)
##                 npar    AIC    BIC  logLik deviance  Chisq Df Pr(>Chisq)    
## model.age          4 6151.2 6177.2 -3071.6   6143.2                         
## cohortmodel.age    5 6118.7 6151.3 -3054.4   6108.7 34.408  1  4.469e-09 ***
## ---
## Signif. codes:  0 '***' 0.001 '**' 0.01 '*' 0.05 '.' 0.1 ' ' 1
```

For each prototype both the LMI and the age cohort terms prove to be
a significant addition to a model containing the other term as a sole
fixed effect. This means that both predictors serve to better predict
dialect change in combination with the other. The LMI prototypes’ slope
estimates change from the those in the base model, and their predictive
power becomes more similar than in the case of the base models; the
coefficients of LMI\_B and LMI\_D decrease, while those of LMI\_A and LMI\_C
increase to values similar to LMI\_B.

Regarding random effects, *SD\_speaker* decreases with the
involvement of age cohort as a fixed effect. This decrease means that a
substantial amount of the variance explained so far only by speaker
random effect is channelled into the age cohort’s effect.

It shows us that it is indeed a significant addition to use LMI in
the prediction of dialect change in addition to an age-cohort-only
model. *AICc* also shows that the models with LMI are of better
model quality. *AICc* decreases the most for LMI\_D.

Demonstrating the defining effect of age cohort on the example of
LMI\_A, the contrast of `exp(0.3469)= 1.414675` and
`exp(0.7645)= 2.14792` means that although increasing the
scaled LMI value by two standard deviations means a 41.5% growth in the
odds of dialect change, changing from the old to the young age cohort
means 114% higher odds of dialect change.

### 4.2.1 Interaction of LMI prototypes and age cohort

We test if adding an interaction term with age cohort makes a
difference with the different LMI prototypes.

**LMIA**

```
minimmodel.age.inter <- glmer(Change ~ LMI_minimal +  Age_cohort.z + LMI_minimal:Age_cohort.z +(1|UID) + (1|Item),
                        data=compara, family=binomial(),
                  control = glmerControl(optimizer = "bobyqa"))

summ(minimmodel.age.inter, digits=4) # interaction * sig
```

|  |  |
| --- | --- |
| Observations | 4983 |
| Dependent variable | Change |
| Type | Mixed effects generalized linear model |
| Family | binomial |
| Link | logit |

|  |  |
| --- | --- |
| AIC | 6128.4167 |
| BIC | 6167.4994 |
| Pseudo-R² (fixed effects) | 0.0480 |
| Pseudo-R² (total) | 0.1965 |

| Fixed Effects | | | | |
| --- | --- | --- | --- | --- |
|  | Est. | S.E. | z val. | p |
| (Intercept) | -0.5059 | 0.1977 | -2.5587 | 0.0105 |
| LMI\_minimal | 0.3742 | 0.0761 | 4.9185 | 0.0000 |
| Age\_cohort.z | 0.7669 | 0.0770 | 9.9648 | 0.0000 |
| LMI\_minimal:Age\_cohort.z | -0.3838 | 0.1520 | -2.5251 | 0.0116 |

| Random Effects | | |
| --- | --- | --- |
| Group | Parameter | Std. Dev. |
| UID | (Intercept) | 0.4814 |
| Item | (Intercept) | 0.6133 |

| Grouping Variables | | |
| --- | --- | --- |
| Group | # groups | ICC |
| UID | 500 | 0.0595 |
| Item | 10 | 0.0965 |

**LMIB**

```
cumulmodel.age.inter <- glmer(Change ~ LMI_cumulated +  Age_cohort.z + LMI_cumulated:Age_cohort.z+ (1|UID) + (1|Item),
                        data=compara, family=binomial(),
                  control = glmerControl(optimizer = "bobyqa"))

summ(cumulmodel.age.inter, digits=4) # interaction . almost sig
```

|  |  |
| --- | --- |
| Observations | 4983 |
| Dependent variable | Change |
| Type | Mixed effects generalized linear model |
| Family | binomial |
| Link | logit |

|  |  |
| --- | --- |
| AIC | 6122.1372 |
| BIC | 6161.2200 |
| Pseudo-R² (fixed effects) | 0.0500 |
| Pseudo-R² (total) | 0.1964 |

| Fixed Effects | | | | |
| --- | --- | --- | --- | --- |
|  | Est. | S.E. | z val. | p |
| (Intercept) | -0.4900 | 0.1979 | -2.4758 | 0.0133 |
| LMI\_cumulated | 0.4781 | 0.0820 | 5.8274 | 0.0000 |
| Age\_cohort.z | 0.6533 | 0.0792 | 8.2494 | 0.0000 |
| LMI\_cumulated:Age\_cohort.z | -0.3004 | 0.1637 | -1.8347 | 0.0665 |

| Random Effects | | |
| --- | --- | --- |
| Group | Parameter | Std. Dev. |
| UID | (Intercept) | 0.4728 |
| Item | (Intercept) | 0.6132 |

| Grouping Variables | | |
| --- | --- | --- |
| Group | # groups | ICC |
| UID | 500 | 0.0575 |
| Item | 10 | 0.0967 |

**LMIC**

```
comprehmodel.age.inter <- glmer(Change ~ LMI_compreh +  Age_cohort.z + LMI_compreh:Age_cohort.z+ (1|UID) + (1|Item),
                        data=compara, family=binomial(),
                  control = glmerControl(optimizer = "bobyqa"))

summ(comprehmodel.age.inter, digits=4) # interaction not sig
```

|  |  |
| --- | --- |
| Observations | 4983 |
| Dependent variable | Change |
| Type | Mixed effects generalized linear model |
| Family | binomial |
| Link | logit |

|  |  |
| --- | --- |
| AIC | 6122.6990 |
| BIC | 6161.7817 |
| Pseudo-R² (fixed effects) | 0.0498 |
| Pseudo-R² (total) | 0.1965 |

| Fixed Effects | | | | |
| --- | --- | --- | --- | --- |
|  | Est. | S.E. | z val. | p |
| (Intercept) | -0.5072 | 0.1977 | -2.5655 | 0.0103 |
| LMI\_compreh | 0.4397 | 0.0764 | 5.7520 | 0.0000 |
| Age\_cohort.z | 0.7543 | 0.0766 | 9.8460 | 0.0000 |
| LMI\_compreh:Age\_cohort.z | -0.2209 | 0.1526 | -1.4480 | 0.1476 |

| Random Effects | | |
| --- | --- | --- |
| Group | Parameter | Std. Dev. |
| UID | (Intercept) | 0.4737 |
| Item | (Intercept) | 0.6132 |

| Grouping Variables | | |
| --- | --- | --- |
| Group | # groups | ICC |
| UID | 500 | 0.0577 |
| Item | 10 | 0.0967 |

**LMID**

```
cohortmodel.age.inter <- glmer(Change ~ LMI_cohort +  Age_cohort.z + LMI_cohort:Age_cohort.z+ (1|UID) + (1|Item),
                        data=compara, family=binomial(),
                  control = glmerControl(optimizer = "bobyqa"))

summ(cohortmodel.age.inter, digits=4) # interaction . almost sig
```

|  |  |
| --- | --- |
| Observations | 4983 |
| Dependent variable | Change |
| Type | Mixed effects generalized linear model |
| Family | binomial |
| Link | logit |

|  |  |
| --- | --- |
| AIC | 6117.7945 |
| BIC | 6156.8772 |
| Pseudo-R² (fixed effects) | 0.0517 |
| Pseudo-R² (total) | 0.1966 |

| Fixed Effects | | | | |
| --- | --- | --- | --- | --- |
|  | Est. | S.E. | z val. | p |
| (Intercept) | -0.4969 | 0.1978 | -2.5125 | 0.0120 |
| LMI\_cohort | 0.4901 | 0.0792 | 6.1900 | 0.0000 |
| Age\_cohort.z | 0.6838 | 0.0774 | 8.8299 | 0.0000 |
| LMI\_cohort:Age\_cohort.z | -0.2725 | 0.1580 | -1.7242 | 0.0847 |

| Random Effects | | |
| --- | --- | --- |
| Group | Parameter | Std. Dev. |
| UID | (Intercept) | 0.4660 |
| Item | (Intercept) | 0.6133 |

| Grouping Variables | | |
| --- | --- | --- |
| Group | # groups | ICC |
| UID | 500 | 0.0559 |
| Item | 10 | 0.0969 |

The interaction provides a significant effect only in the case of
LMI\_A. The estimates for LMI however grow for cohort-based, minimal and
comprehensive model.

Plotting the interaction of age cohort and LMI\_A.

```
plot(allEffects(minimmodel.age.inter))
```

Regarding random effects, *SD\_speaker* decreases with the
involvement of age cohort as a fixed effect. This decrease means that a
substantial amount of the variance explained so far only by speaker
random effect is channelled into the age cohort’s effect.

```
# basically by this we define again the base model
minimmodel.age.inter1 <- update(minimmodel.age.inter, ~.-LMI_minimal:Age_cohort.z)

anova(minimmodel.age.inter, minimmodel.age.inter1)
```

```
## Data: compara
## Models:
## minimmodel.age.inter1: Change ~ LMI_minimal + Age_cohort.z + (1 | UID) + (1 | Item)
## minimmodel.age.inter: Change ~ LMI_minimal + Age_cohort.z + LMI_minimal:Age_cohort.z + (1 | UID) + (1 | Item)
##                       npar    AIC    BIC  logLik deviance  Chisq Df Pr(>Chisq)
## minimmodel.age.inter1    5 6132.7 6165.3 -3061.4   6122.7                     
## minimmodel.age.inter     6 6128.4 6167.5 -3058.2   6116.4 6.3064  1    0.01203
##                        
## minimmodel.age.inter1  
## minimmodel.age.inter  *
## ---
## Signif. codes:  0 '***' 0.001 '**' 0.01 '*' 0.05 '.' 0.1 ' ' 1
```

```
AIC(minimmodel.age.inter, minimmodel.age.inter1)
```

```
##                       df      AIC
## minimmodel.age.inter   6 6128.417
## minimmodel.age.inter1  5 6132.723
```

```
MuMIn::AICc(minimmodel.age.inter, minimmodel.age.inter1)
```

```
##                       df     AICc
## minimmodel.age.inter   6 6128.434
## minimmodel.age.inter1  5 6132.735
```

The addition of the interaction is a significant for LMI\_A. This is
shown by *AIC* and *AICc* as well.

## 4.3 Full models

Adding the survey design variables (age cohort, gender and
educational background) as fixed effects.

**LMIA**

```
#LMI^A
minim.model2 <- glmer(Change ~ 
                  LMI_minimal +
                  Age_cohort.z + 
                  Sex.z +
                  Edu_bkgr_2cat.z +
                  (1|UID) + (1|Item), 
                data=compara, family=binomial(),
                control = glmerControl(optimizer = "bobyqa")
)

summ(minim.model2, digits=4)
```

|  |  |
| --- | --- |
| Observations | 4983 |
| Dependent variable | Change |
| Type | Mixed effects generalized linear model |
| Family | binomial |
| Link | logit |

|  |  |
| --- | --- |
| AIC | 6129.6321 |
| BIC | 6175.2286 |
| Pseudo-R² (fixed effects) | 0.0476 |
| Pseudo-R² (total) | 0.1960 |

| Fixed Effects | | | | |
| --- | --- | --- | --- | --- |
|  | Est. | S.E. | z val. | p |
| (Intercept) | -0.5232 | 0.1980 | -2.6426 | 0.0082 |
| LMI\_minimal | 0.3486 | 0.0755 | 4.6152 | 0.0000 |
| Age\_cohort.z | 0.7691 | 0.0771 | 9.9755 | 0.0000 |
| Sex.z | 0.2022 | 0.0777 | 2.6025 | 0.0093 |
| Edu\_bkgr\_2cat.z | 0.0901 | 0.0811 | 1.1105 | 0.2668 |

| Random Effects | | |
| --- | --- | --- |
| Group | Parameter | Std. Dev. |
| UID | (Intercept) | 0.4809 |
| Item | (Intercept) | 0.6130 |

| Grouping Variables | | |
| --- | --- | --- |
| Group | # groups | ICC |
| UID | 500 | 0.0593 |
| Item | 10 | 0.0964 |

**LMIB**

```
#LMI^B
cumul.model2 <- glmer(Change ~ LMI_cumulated +Age_cohort.z + Sex.z +Edu_bkgr_2cat.z +
                  (1|UID) + (1|Item), 
                data=compara, family=binomial(),
                control = glmerControl(optimizer = "bobyqa")
)

summ(cumul.model2, digits=4)
```

|  |  |
| --- | --- |
| Observations | 4983 |
| Dependent variable | Change |
| Type | Mixed effects generalized linear model |
| Family | binomial |
| Link | logit |

|  |  |
| --- | --- |
| AIC | 6119.7646 |
| BIC | 6165.3611 |
| Pseudo-R² (fixed effects) | 0.0509 |
| Pseudo-R² (total) | 0.1959 |

| Fixed Effects | | | | |
| --- | --- | --- | --- | --- |
|  | Est. | S.E. | z val. | p |
| (Intercept) | -0.5291 | 0.1979 | -2.6738 | 0.0075 |
| LMI\_cumulated | 0.4395 | 0.0784 | 5.6041 | 0.0000 |
| Age\_cohort.z | 0.6681 | 0.0789 | 8.4704 | 0.0000 |
| Sex.z | 0.1919 | 0.0769 | 2.4942 | 0.0126 |
| Edu\_bkgr\_2cat.z | 0.1374 | 0.0806 | 1.7055 | 0.0881 |

| Random Effects | | |
| --- | --- | --- |
| Group | Parameter | Std. Dev. |
| UID | (Intercept) | 0.4663 |
| Item | (Intercept) | 0.6129 |

| Grouping Variables | | |
| --- | --- | --- |
| Group | # groups | ICC |
| UID | 500 | 0.0560 |
| Item | 10 | 0.0967 |

**LMIC**

```
#LMI^C
compreh.model2 <- glmer(Change ~ LMI_compreh +Age_cohort.z + Sex.z +Edu_bkgr_2cat.z +
                  (1|UID) + (1|Item), 
                data=compara, family=binomial(),
                control = glmerControl(optimizer = "bobyqa")
)

summ(compreh.model2, digits=4)
```

|  |  |
| --- | --- |
| Observations | 4983 |
| Dependent variable | Change |
| Type | Mixed effects generalized linear model |
| Family | binomial |
| Link | logit |

|  |  |
| --- | --- |
| AIC | 6120.1836 |
| BIC | 6165.7801 |
| Pseudo-R² (fixed effects) | 0.0509 |
| Pseudo-R² (total) | 0.1960 |

| Fixed Effects | | | | |
| --- | --- | --- | --- | --- |
|  | Est. | S.E. | z val. | p |
| (Intercept) | -0.5275 | 0.1979 | -2.6653 | 0.0077 |
| LMI\_compreh | 0.4203 | 0.0755 | 5.5693 | 0.0000 |
| Age\_cohort.z | 0.7596 | 0.0764 | 9.9385 | 0.0000 |
| Sex.z | 0.1797 | 0.0770 | 2.3325 | 0.0197 |
| Edu\_bkgr\_2cat.z | 0.1228 | 0.0804 | 1.5264 | 0.1269 |

| Random Effects | | |
| --- | --- | --- |
| Group | Parameter | Std. Dev. |
| UID | (Intercept) | 0.4672 |
| Item | (Intercept) | 0.6130 |

| Grouping Variables | | |
| --- | --- | --- |
| Group | # groups | ICC |
| UID | 500 | 0.0562 |
| Item | 10 | 0.0967 |

**LMID**

```
#LMI^D
cohort.model2 <- glmer(Change ~ LMI_cohort +Age_cohort.z + Sex.z +Edu_bkgr_2cat.z +
                  (1|UID) + (1|Item), 
                data=compara, family=binomial(),
                control = glmerControl(optimizer = "bobyqa")
)

summ(cohort.model2, digits=4)
```

|  |  |
| --- | --- |
| Observations | 4983 |
| Dependent variable | Change |
| Type | Mixed effects generalized linear model |
| Family | binomial |
| Link | logit |

|  |  |
| --- | --- |
| AIC | 6115.6341 |
| BIC | 6161.2306 |
| Pseudo-R² (fixed effects) | 0.0525 |
| Pseudo-R² (total) | 0.1960 |

| Fixed Effects | | | | |
| --- | --- | --- | --- | --- |
|  | Est. | S.E. | z val. | p |
| (Intercept) | -0.5278 | 0.1979 | -2.6673 | 0.0076 |
| LMI\_cohort | 0.4600 | 0.0770 | 5.9714 | 0.0000 |
| Age\_cohort.z | 0.6934 | 0.0773 | 8.9693 | 0.0000 |
| Sex.z | 0.1859 | 0.0766 | 2.4262 | 0.0153 |
| Edu\_bkgr\_2cat.z | 0.1264 | 0.0801 | 1.5779 | 0.1146 |

| Random Effects | | |
| --- | --- | --- |
| Group | Parameter | Std. Dev. |
| UID | (Intercept) | 0.4602 |
| Item | (Intercept) | 0.6130 |

| Grouping Variables | | |
| --- | --- | --- |
| Group | # groups | ICC |
| UID | 500 | 0.0546 |
| Item | 10 | 0.0969 |

Effect plots:

```
plot(allEffects(minim.model2))
```

```
plot(allEffects(cumul.model2))
```

```
plot(allEffects(compreh.model2))
```

```
plot(allEffects(cohort.model2))
```

Showing a summary with the already **exponentiated**
estimates in `glmer` results, using the `jtools`
package.

**LMIA**

```
summ(minim.model2, exp=T, digits=4)
```

|  |  |
| --- | --- |
| Observations | 4983 |
| Dependent variable | Change |
| Type | Mixed effects generalized linear model |
| Family | binomial |
| Link | logit |

|  |  |
| --- | --- |
| AIC | 6129.6321 |
| BIC | 6175.2286 |
| Pseudo-R² (fixed effects) | 0.0476 |
| Pseudo-R² (total) | 0.1960 |

| Fixed Effects | | | | |
| --- | --- | --- | --- | --- |
|  | exp(Est.) | S.E. | z val. | p |
| (Intercept) | 0.5926 | 0.1980 | -2.6426 | 0.0082 |
| LMI\_minimal | 1.4171 | 0.0755 | 4.6152 | 0.0000 |
| Age\_cohort.z | 2.1577 | 0.0771 | 9.9755 | 0.0000 |
| Sex.z | 1.2241 | 0.0777 | 2.6025 | 0.0093 |
| Edu\_bkgr\_2cat.z | 1.0942 | 0.0811 | 1.1105 | 0.2668 |

| Random Effects | | |
| --- | --- | --- |
| Group | Parameter | Std. Dev. |
| UID | (Intercept) | 0.4809 |
| Item | (Intercept) | 0.6130 |

| Grouping Variables | | |
| --- | --- | --- |
| Group | # groups | ICC |
| UID | 500 | 0.0593 |
| Item | 10 | 0.0964 |

**LMIB**

```
summ(cumul.model2, exp=T, digits=4)
```

|  |  |
| --- | --- |
| Observations | 4983 |
| Dependent variable | Change |
| Type | Mixed effects generalized linear model |
| Family | binomial |
| Link | logit |

|  |  |
| --- | --- |
| AIC | 6119.7646 |
| BIC | 6165.3611 |
| Pseudo-R² (fixed effects) | 0.0509 |
| Pseudo-R² (total) | 0.1959 |

| Fixed Effects | | | | |
| --- | --- | --- | --- | --- |
|  | exp(Est.) | S.E. | z val. | p |
| (Intercept) | 0.5891 | 0.1979 | -2.6738 | 0.0075 |
| LMI\_cumulated | 1.5520 | 0.0784 | 5.6041 | 0.0000 |
| Age\_cohort.z | 1.9506 | 0.0789 | 8.4704 | 0.0000 |
| Sex.z | 1.2115 | 0.0769 | 2.4942 | 0.0126 |
| Edu\_bkgr\_2cat.z | 1.1473 | 0.0806 | 1.7055 | 0.0881 |

| Random Effects | | |
| --- | --- | --- |
| Group | Parameter | Std. Dev. |
| UID | (Intercept) | 0.4663 |
| Item | (Intercept) | 0.6129 |

| Grouping Variables | | |
| --- | --- | --- |
| Group | # groups | ICC |
| UID | 500 | 0.0560 |
| Item | 10 | 0.0967 |

**LMIC**

```
summ(compreh.model2, exp=T, digits=4)
```

|  |  |
| --- | --- |
| Observations | 4983 |
| Dependent variable | Change |
| Type | Mixed effects generalized linear model |
| Family | binomial |
| Link | logit |

|  |  |
| --- | --- |
| AIC | 6120.1836 |
| BIC | 6165.7801 |
| Pseudo-R² (fixed effects) | 0.0509 |
| Pseudo-R² (total) | 0.1960 |

| Fixed Effects | | | | |
| --- | --- | --- | --- | --- |
|  | exp(Est.) | S.E. | z val. | p |
| (Intercept) | 0.5901 | 0.1979 | -2.6653 | 0.0077 |
| LMI\_compreh | 1.5224 | 0.0755 | 5.5693 | 0.0000 |
| Age\_cohort.z | 2.1374 | 0.0764 | 9.9385 | 0.0000 |
| Sex.z | 1.1968 | 0.0770 | 2.3325 | 0.0197 |
| Edu\_bkgr\_2cat.z | 1.1306 | 0.0804 | 1.5264 | 0.1269 |

| Random Effects | | |
| --- | --- | --- |
| Group | Parameter | Std. Dev. |
| UID | (Intercept) | 0.4672 |
| Item | (Intercept) | 0.6130 |

| Grouping Variables | | |
| --- | --- | --- |
| Group | # groups | ICC |
| UID | 500 | 0.0562 |
| Item | 10 | 0.0967 |

**LMID**

```
summ(cohort.model2, exp=T, digits=4)
```

|  |  |
| --- | --- |
| Observations | 4983 |
| Dependent variable | Change |
| Type | Mixed effects generalized linear model |
| Family | binomial |
| Link | logit |

|  |  |
| --- | --- |
| AIC | 6115.6341 |
| BIC | 6161.2306 |
| Pseudo-R² (fixed effects) | 0.0525 |
| Pseudo-R² (total) | 0.1960 |

| Fixed Effects | | | | |
| --- | --- | --- | --- | --- |
|  | exp(Est.) | S.E. | z val. | p |
| (Intercept) | 0.5899 | 0.1979 | -2.6673 | 0.0076 |
| LMI\_cohort | 1.5841 | 0.0770 | 5.9714 | 0.0000 |
| Age\_cohort.z | 2.0005 | 0.0773 | 8.9693 | 0.0000 |
| Sex.z | 1.2043 | 0.0766 | 2.4262 | 0.0153 |
| Edu\_bkgr\_2cat.z | 1.1348 | 0.0801 | 1.5779 | 0.1146 |

| Random Effects | | |
| --- | --- | --- |
| Group | Parameter | Std. Dev. |
| UID | (Intercept) | 0.4602 |
| Item | (Intercept) | 0.6130 |

| Grouping Variables | | |
| --- | --- | --- |
| Group | # groups | ICC |
| UID | 500 | 0.0546 |
| Item | 10 | 0.0969 |

Fixed effects prove to be significant predictors, except for the
education background wich is not significant for any of the prototypes.
The LMI prototypes’ effects are highly significant; their estimates grow
slightly compared to the reduced models and their *SE* stays low.
Based on *AIC* values the quality of the model with LMI\_D emerges
as the best. In the case of each LMI prototype, the already high
predictive power of age cohort grows slightly with the inclusion of
gender and educational background and maintains its decisive effect. The
LMI prototypes’ predictive power in increasing order is A, C, B, D.

Exponentiating the estimates of the LMI\_D prototype, we can compare
the effects of the fixed effects in a plastic manner.

When other effects are kept constant, differences across age cohorts,
through the exponentiation of beta-estimates and *SD*,
`exp(0.6934)=2.0005 and exp(0.0773)=1.080366`, respectively,
means that the younger cohort has more than 100% higher odds for dialect
change, with a 8% standard deviation.

Regarding sex,
`exp(0.1859)=1.2043 and exp(0.0766)=1.07961`, means that
males have more than 20% higher odds for dialect change, with a 8%
standard deviation.

Regarding educational background,
`exp(0.1264)=1.1348 and exp(0.0801)=1.083395`, means that
males have more than 13% higher odds for dialect change, with a 8%
standard deviation. Because this SD is so high compared to the
difference between the categories, the effect cannot be taken as
significant.

Regarding LMI\_D, an increase of two standard deviations in its value
`exp(0.4600)=1.5841 and exp(0.0770)=1.080042`, means that
males have more than 58% higher odds for dialect change, with a 8%
standard deviation. Thus, LMI as a predictor is stronger than sex but
not as strong as age cohorts.

Checking **Variance inflation factors** (VIF).

```
library(car)
car::vif(minim.model2)
```

```
##     LMI_minimal    Age_cohort.z           Sex.z Edu_bkgr_2cat.z 
##        1.002969        1.006010        1.039073        1.043035
```

```
car::vif(compreh.model2)
```

```
##     LMI_compreh    Age_cohort.z           Sex.z Edu_bkgr_2cat.z 
##        1.007557        1.006521        1.039496        1.045160
```

```
car::vif(cumul.model2)
```

```
##   LMI_cumulated    Age_cohort.z           Sex.z Edu_bkgr_2cat.z 
##        1.079975        1.073344        1.038441        1.050141
```

```
car::vif(cohort.model2)
```

```
##      LMI_cohort    Age_cohort.z           Sex.z Edu_bkgr_2cat.z 
##        1.040680        1.039262        1.038396        1.045795
```

VIF-values look good, all of them staying close to 1, thus no
problems with collinearity are expected.

Looking at whether the LMI is a useful addition in models where the
design variables are already present as fixed effects.

```
model.woLMI <- glmer(Change ~ Age_cohort.z + Sex.z +Edu_bkgr_2cat.z +
                  (1|UID) + (1|Item),
                        data=compara, family=binomial(),
                  control = glmerControl(optimizer = "bobyqa"))
summ(model.woLMI, digits=4)
```

|  |  |
| --- | --- |
| Observations | 4983 |
| Dependent variable | Change |
| Type | Mixed effects generalized linear model |
| Family | binomial |
| Link | logit |

|  |  |
| --- | --- |
| AIC | 6148.5239 |
| BIC | 6187.6066 |
| Pseudo-R² (fixed effects) | 0.0402 |
| Pseudo-R² (total) | 0.1957 |

| Fixed Effects | | | | |
| --- | --- | --- | --- | --- |
|  | Est. | S.E. | z val. | p |
| (Intercept) | -0.5245 | 0.1981 | -2.6477 | 0.0081 |
| Age\_cohort.z | 0.7918 | 0.0785 | 10.0829 | 0.0000 |
| Sex.z | 0.1963 | 0.0792 | 2.4775 | 0.0132 |
| Edu\_bkgr\_2cat.z | 0.1003 | 0.0827 | 1.2128 | 0.2252 |

| Random Effects | | |
| --- | --- | --- |
| Group | Parameter | Std. Dev. |
| UID | (Intercept) | 0.5104 |
| Item | (Intercept) | 0.6128 |

| Grouping Variables | | |
| --- | --- | --- |
| Group | # groups | ICC |
| UID | 500 | 0.0664 |
| Item | 10 | 0.0957 |

```
# Let's see if adding the different LMI's is significant, using likelihood-ratio testing (LRT) with chi-squares
anova(model.woLMI, minim.model2)
```

```
## Data: compara
## Models:
## model.woLMI: Change ~ Age_cohort.z + Sex.z + Edu_bkgr_2cat.z + (1 | UID) + (1 | Item)
## minim.model2: Change ~ LMI_minimal + Age_cohort.z + Sex.z + Edu_bkgr_2cat.z + (1 | UID) + (1 | Item)
##              npar    AIC    BIC  logLik deviance  Chisq Df Pr(>Chisq)    
## model.woLMI     6 6148.5 6187.6 -3068.3   6136.5                         
## minim.model2    7 6129.6 6175.2 -3057.8   6115.6 20.892  1   4.86e-06 ***
## ---
## Signif. codes:  0 '***' 0.001 '**' 0.01 '*' 0.05 '.' 0.1 ' ' 1
```

```
anova(model.woLMI, cumul.model2)
```

```
## Data: compara
## Models:
## model.woLMI: Change ~ Age_cohort.z + Sex.z + Edu_bkgr_2cat.z + (1 | UID) + (1 | Item)
## cumul.model2: Change ~ LMI_cumulated + Age_cohort.z + Sex.z + Edu_bkgr_2cat.z + (1 | UID) + (1 | Item)
##              npar    AIC    BIC  logLik deviance  Chisq Df Pr(>Chisq)    
## model.woLMI     6 6148.5 6187.6 -3068.3   6136.5                         
## cumul.model2    7 6119.8 6165.4 -3052.9   6105.8 30.759  1  2.921e-08 ***
## ---
## Signif. codes:  0 '***' 0.001 '**' 0.01 '*' 0.05 '.' 0.1 ' ' 1
```

```
anova(model.woLMI, compreh.model2)
```

```
## Data: compara
## Models:
## model.woLMI: Change ~ Age_cohort.z + Sex.z + Edu_bkgr_2cat.z + (1 | UID) + (1 | Item)
## compreh.model2: Change ~ LMI_compreh + Age_cohort.z + Sex.z + Edu_bkgr_2cat.z + (1 | UID) + (1 | Item)
##                npar    AIC    BIC  logLik deviance Chisq Df Pr(>Chisq)    
## model.woLMI       6 6148.5 6187.6 -3068.3   6136.5                        
## compreh.model2    7 6120.2 6165.8 -3053.1   6106.2 30.34  1  3.625e-08 ***
## ---
## Signif. codes:  0 '***' 0.001 '**' 0.01 '*' 0.05 '.' 0.1 ' ' 1
```

```
anova(model.woLMI, cohort.model2)
```

```
## Data: compara
## Models:
## model.woLMI: Change ~ Age_cohort.z + Sex.z + Edu_bkgr_2cat.z + (1 | UID) + (1 | Item)
## cohort.model2: Change ~ LMI_cohort + Age_cohort.z + Sex.z + Edu_bkgr_2cat.z + (1 | UID) + (1 | Item)
##               npar    AIC    BIC  logLik deviance Chisq Df Pr(>Chisq)    
## model.woLMI      6 6148.5 6187.6 -3068.3   6136.5                        
## cohort.model2    7 6115.6 6161.2 -3050.8   6101.6 34.89  1  3.489e-09 ***
## ---
## Signif. codes:  0 '***' 0.001 '**' 0.01 '*' 0.05 '.' 0.1 ' ' 1
```

```
AIC(model.woLMI, minim.model2)
```

```
##              df      AIC
## model.woLMI   6 6148.524
## minim.model2  7 6129.632
```

```
AIC(model.woLMI, cumul.model2)
```

```
##              df      AIC
## model.woLMI   6 6148.524
## cumul.model2  7 6119.765
```

```
AIC(model.woLMI, compreh.model2)
```

```
##                df      AIC
## model.woLMI     6 6148.524
## compreh.model2  7 6120.184
```

```
AIC(model.woLMI, cohort.model2)
```

```
##               df      AIC
## model.woLMI    6 6148.524
## cohort.model2  7 6115.634
```

We see that removing LMI does not mean that the education predictor
becomes significant.

*AIC* values show that adding the LMI in the model makes
sense. For all four models, based on *AIC* values and
*χ2*-tests, adding the LMI prototypes in the model as fixed
effects proves to be significant.

### 4.3.1 Interaction of LMI with age cohorts

#### 4.3.1.1 LMIA (minimal prototype)

```
minim.model2.inter <- glmer(Change ~ LMI_minimal +Age_cohort.z + Sex.z +Edu_bkgr_2cat.z +
                        LMI_minimal:Age_cohort.z +
                  (1|UID) + (1|Item), 
                data=compara, family=binomial(),
                control = glmerControl(optimizer = "bobyqa"))

summ(minim.model2.inter, digits=4)
```

|  |  |
| --- | --- |
| Observations | 4983 |
| Dependent variable | Change |
| Type | Mixed effects generalized linear model |
| Family | binomial |
| Link | logit |

|  |  |
| --- | --- |
| AIC | 6125.3463 |
| BIC | 6177.4566 |
| Pseudo-R² (fixed effects) | 0.0503 |
| Pseudo-R² (total) | 0.1964 |

| Fixed Effects | | | | |
| --- | --- | --- | --- | --- |
|  | Est. | S.E. | z val. | p |
| (Intercept) | -0.5186 | 0.1980 | -2.6193 | 0.0088 |
| LMI\_minimal | 0.3754 | 0.0756 | 4.9662 | 0.0000 |
| Age\_cohort.z | 0.7716 | 0.0766 | 10.0723 | 0.0000 |
| Sex.z | 0.1998 | 0.0772 | 2.5886 | 0.0096 |
| Edu\_bkgr\_2cat.z | 0.0921 | 0.0806 | 1.1432 | 0.2529 |
| LMI\_minimal:Age\_cohort.z | -0.3805 | 0.1509 | -2.5207 | 0.0117 |

| Random Effects | | |
| --- | --- | --- |
| Group | Parameter | Std. Dev. |
| UID | (Intercept) | 0.4710 |
| Item | (Intercept) | 0.6132 |

| Grouping Variables | | |
| --- | --- | --- |
| Group | # groups | ICC |
| UID | 500 | 0.0571 |
| Item | 10 | 0.0967 |

```
plot(allEffects(minim.model2.inter))
```

```
anova(minim.model2.inter, minim.model2)
```

```
## Data: compara
## Models:
## minim.model2: Change ~ LMI_minimal + Age_cohort.z + Sex.z + Edu_bkgr_2cat.z + (1 | UID) + (1 | Item)
## minim.model2.inter: Change ~ LMI_minimal + Age_cohort.z + Sex.z + Edu_bkgr_2cat.z + LMI_minimal:Age_cohort.z + (1 | UID) + (1 | Item)
##                    npar    AIC    BIC  logLik deviance  Chisq Df Pr(>Chisq)  
## minim.model2          7 6129.6 6175.2 -3057.8   6115.6                       
## minim.model2.inter    8 6125.3 6177.5 -3054.7   6109.3 6.2858  1    0.01217 *
## ---
## Signif. codes:  0 '***' 0.001 '**' 0.01 '*' 0.05 '.' 0.1 ' ' 1
```

```
AIC(minim.model2.inter, minim.model2)
```

```
##                    df      AIC
## minim.model2.inter  8 6125.346
## minim.model2        7 6129.632
```

```
AICc(minim.model2.inter, minim.model2)
```

```
##                    df     AICc
## minim.model2.inter  8 6125.375
## minim.model2        7 6129.655
```

Adding the interaction term still means a significant addition for
LMI\_A.  
For all the other LMI prototypes, the interaction term is not
significant.

#### 4.3.1.2 LMIB (cumulative prototype)

```
cumul.model2.inter <- glmer(Change ~ LMI_cumulated +Age_cohort.z + Sex.z +Edu_bkgr_2cat.z +
                        LMI_cumulated:Age_cohort.z +
                  (1|UID) + (1|Item), 
                data=compara, family=binomial(),
                control = glmerControl(optimizer = "bobyqa"))

summ(cumul.model2.inter, digits=4)
```

|  |  |
| --- | --- |
| Observations | 4983 |
| Dependent variable | Change |
| Type | Mixed effects generalized linear model |
| Family | binomial |
| Link | logit |

|  |  |
| --- | --- |
| AIC | 6118.9734 |
| BIC | 6171.0837 |
| Pseudo-R² (fixed effects) | 0.0523 |
| Pseudo-R² (total) | 0.1963 |

| Fixed Effects | | | | |
| --- | --- | --- | --- | --- |
|  | Est. | S.E. | z val. | p |
| (Intercept) | -0.5113 | 0.1982 | -2.5796 | 0.0099 |
| LMI\_cumulated | 0.4807 | 0.0819 | 5.8717 | 0.0000 |
| Age\_cohort.z | 0.6599 | 0.0787 | 8.3838 | 0.0000 |
| Sex.z | 0.1803 | 0.0770 | 2.3400 | 0.0193 |
| Edu\_bkgr\_2cat.z | 0.1399 | 0.0804 | 1.7406 | 0.0818 |
| LMI\_cumulated:Age\_cohort.z | -0.2738 | 0.1633 | -1.6763 | 0.0937 |

| Random Effects | | |
| --- | --- | --- |
| Group | Parameter | Std. Dev. |
| UID | (Intercept) | 0.4622 |
| Item | (Intercept) | 0.6131 |

| Grouping Variables | | |
| --- | --- | --- |
| Group | # groups | ICC |
| UID | 500 | 0.0551 |
| Item | 10 | 0.0969 |

#### 4.3.1.3 LMIC (comprehensive prototype)

```
compreh.model2.inter <- glmer(Change ~ LMI_compreh +Age_cohort.z + Sex.z +Edu_bkgr_2cat.z +
                        LMI_compreh:Age_cohort.z +
                  (1|UID) + (1|Item), 
                data=compara, family=binomial(),
                control = glmerControl(optimizer = "bobyqa"))

summ(compreh.model2.inter, digits=4)
```

|  |  |
| --- | --- |
| Observations | 4983 |
| Dependent variable | Change |
| Type | Mixed effects generalized linear model |
| Family | binomial |
| Link | logit |

|  |  |
| --- | --- |
| AIC | 6120.4007 |
| BIC | 6172.5110 |
| Pseudo-R² (fixed effects) | 0.0519 |
| Pseudo-R² (total) | 0.1964 |

| Fixed Effects | | | | |
| --- | --- | --- | --- | --- |
|  | Est. | S.E. | z val. | p |
| (Intercept) | -0.5250 | 0.1979 | -2.6523 | 0.0080 |
| LMI\_compreh | 0.4368 | 0.0762 | 5.7303 | 0.0000 |
| Age\_cohort.z | 0.7613 | 0.0763 | 9.9772 | 0.0000 |
| Sex.z | 0.1716 | 0.0771 | 2.2255 | 0.0260 |
| Edu\_bkgr\_2cat.z | 0.1266 | 0.0803 | 1.5762 | 0.1150 |
| LMI\_compreh:Age\_cohort.z | -0.2039 | 0.1523 | -1.3391 | 0.1805 |

| Random Effects | | |
| --- | --- | --- |
| Group | Parameter | Std. Dev. |
| UID | (Intercept) | 0.4644 |
| Item | (Intercept) | 0.6132 |

| Grouping Variables | | |
| --- | --- | --- |
| Group | # groups | ICC |
| UID | 500 | 0.0556 |
| Item | 10 | 0.0969 |

#### 4.3.1.4 LMID (cohort-based prototype)

```
cohort.model2.inter <- glmer(Change ~ LMI_cohort +Age_cohort.z + Sex.z +Edu_bkgr_2cat.z +
                        LMI_cohort:Age_cohort.z +
                  (1|UID) + (1|Item), 
                data=compara, family=binomial(),
                control = glmerControl(optimizer = "bobyqa"))

summ(cohort.model2.inter, digits=4)
```

|  |  |
| --- | --- |
| Observations | 4983 |
| Dependent variable | Change |
| Type | Mixed effects generalized linear model |
| Family | binomial |
| Link | logit |

|  |  |
| --- | --- |
| AIC | 6115.1313 |
| BIC | 6167.2416 |
| Pseudo-R² (fixed effects) | 0.0539 |
| Pseudo-R² (total) | 0.1965 |

| Fixed Effects | | | | |
| --- | --- | --- | --- | --- |
|  | Est. | S.E. | z val. | p |
| (Intercept) | -0.5154 | 0.1980 | -2.6022 | 0.0093 |
| LMI\_cohort | 0.4886 | 0.0789 | 6.1954 | 0.0000 |
| Age\_cohort.z | 0.6907 | 0.0771 | 8.9639 | 0.0000 |
| Sex.z | 0.1785 | 0.0766 | 2.3321 | 0.0197 |
| Edu\_bkgr\_2cat.z | 0.1243 | 0.0799 | 1.5553 | 0.1199 |
| LMI\_cohort:Age\_cohort.z | -0.2496 | 0.1573 | -1.5870 | 0.1125 |

| Random Effects | | |
| --- | --- | --- |
| Group | Parameter | Std. Dev. |
| UID | (Intercept) | 0.4560 |
| Item | (Intercept) | 0.6132 |

| Grouping Variables | | |
| --- | --- | --- |
| Group | # groups | ICC |
| UID | 500 | 0.0537 |
| Item | 10 | 0.0971 |

### 4.3.2 Interaction of sex and age cohorts

#### 4.3.2.1 LMIA (minimal prototype)

```
minim.model2_sexage <- glmer(Change ~ LMI_minimal +Age_cohort.z + Sex.z +Edu_bkgr_2cat.z +
                        Sex.z:Age_cohort.z +
                  (1|UID) + (1|Item), 
                data=compara, family=binomial(),
                control = glmerControl(optimizer = "bobyqa"))

summ(minim.model2_sexage, digits=4)
```

|  |  |
| --- | --- |
| Observations | 4983 |
| Dependent variable | Change |
| Type | Mixed effects generalized linear model |
| Family | binomial |
| Link | logit |

|  |  |
| --- | --- |
| AIC | 6129.8905 |
| BIC | 6182.0008 |
| Pseudo-R² (fixed effects) | 0.0480 |
| Pseudo-R² (total) | 0.1958 |

| Fixed Effects | | | | |
| --- | --- | --- | --- | --- |
|  | Est. | S.E. | z val. | p |
| (Intercept) | -0.5191 | 0.1980 | -2.6224 | 0.0087 |
| LMI\_minimal | 0.3507 | 0.0754 | 4.6499 | 0.0000 |
| Age\_cohort.z | 0.7663 | 0.0770 | 9.9534 | 0.0000 |
| Sex.z | 0.1917 | 0.0780 | 2.4595 | 0.0139 |
| Edu\_bkgr\_2cat.z | 0.0645 | 0.0833 | 0.7743 | 0.4387 |
| Age\_cohort.z:Sex.z | 0.2075 | 0.1567 | 1.3246 | 0.1853 |

| Random Effects | | |
| --- | --- | --- |
| Group | Parameter | Std. Dev. |
| UID | (Intercept) | 0.4784 |
| Item | (Intercept) | 0.6129 |

| Grouping Variables | | |
| --- | --- | --- |
| Group | # groups | ICC |
| UID | 500 | 0.0588 |
| Item | 10 | 0.0965 |

```
plot(allEffects(minim.model2_sexage))
```

```
anova(minim.model2_sexage, minim.model2)
```

```
## Data: compara
## Models:
## minim.model2: Change ~ LMI_minimal + Age_cohort.z + Sex.z + Edu_bkgr_2cat.z + (1 | UID) + (1 | Item)
## minim.model2_sexage: Change ~ LMI_minimal + Age_cohort.z + Sex.z + Edu_bkgr_2cat.z + Sex.z:Age_cohort.z + (1 | UID) + (1 | Item)
##                     npar    AIC    BIC  logLik deviance  Chisq Df Pr(>Chisq)
## minim.model2           7 6129.6 6175.2 -3057.8   6115.6                     
## minim.model2_sexage    8 6129.9 6182.0 -3056.9   6113.9 1.7416  1     0.1869
```

```
AIC(minim.model2_sexage, minim.model2)
```

```
##                     df      AIC
## minim.model2_sexage  8 6129.891
## minim.model2         7 6129.632
```

```
AICc(minim.model2_sexage, minim.model2)
```

```
##                     df     AICc
## minim.model2_sexage  8 6129.919
## minim.model2         7 6129.655
```

#### 4.3.2.2 LMIB (cumulative prototype)

```
cumul.model2_sexage <- glmer(Change ~ LMI_cumulated +Age_cohort.z + Sex.z +Edu_bkgr_2cat.z +
                        Sex.z:Age_cohort.z +
                  (1|UID) + (1|Item), 
                data=compara, family=binomial(),
                control = glmerControl(optimizer = "bobyqa"))

summ(cumul.model2_sexage, digits=4)
```

|  |  |
| --- | --- |
| Observations | 4983 |
| Dependent variable | Change |
| Type | Mixed effects generalized linear model |
| Family | binomial |
| Link | logit |

|  |  |
| --- | --- |
| AIC | 6119.1795 |
| BIC | 6171.2898 |
| Pseudo-R² (fixed effects) | 0.0515 |
| Pseudo-R² (total) | 0.1956 |

| Fixed Effects | | | | |
| --- | --- | --- | --- | --- |
|  | Est. | S.E. | z val. | p |
| (Intercept) | -0.5242 | 0.1978 | -2.6498 | 0.0081 |
| LMI\_cumulated | 0.4479 | 0.0784 | 5.7101 | 0.0000 |
| Age\_cohort.z | 0.6625 | 0.0787 | 8.4145 | 0.0000 |
| Sex.z | 0.1791 | 0.0771 | 2.3223 | 0.0202 |
| Edu\_bkgr\_2cat.z | 0.1073 | 0.0826 | 1.2997 | 0.1937 |
| Age\_cohort.z:Sex.z | 0.2508 | 0.1554 | 1.6140 | 0.1065 |

| Random Effects | | |
| --- | --- | --- |
| Group | Parameter | Std. Dev. |
| UID | (Intercept) | 0.4624 |
| Item | (Intercept) | 0.6127 |

| Grouping Variables | | |
| --- | --- | --- |
| Group | # groups | ICC |
| UID | 500 | 0.0551 |
| Item | 10 | 0.0968 |

```
plot(allEffects(cumul.model2_sexage))
```

#### 4.3.2.3 LMIC (comprehensive prototype)

```
compreh.model2_sexage <- glmer(Change ~ LMI_compreh +Age_cohort.z + Sex.z +Edu_bkgr_2cat.z +
                        Sex.z:Age_cohort.z +
                  (1|UID) + (1|Item), 
                data=compara, family=binomial(),
                control = glmerControl(optimizer = "bobyqa"))

summ(compreh.model2_sexage, digits=4)
```

|  |  |
| --- | --- |
| Observations | 4983 |
| Dependent variable | Change |
| Type | Mixed effects generalized linear model |
| Family | binomial |
| Link | logit |

|  |  |
| --- | --- |
| AIC | 6119.5653 |
| BIC | 6171.6756 |
| Pseudo-R² (fixed effects) | 0.0515 |
| Pseudo-R² (total) | 0.1958 |

| Fixed Effects | | | | |
| --- | --- | --- | --- | --- |
|  | Est. | S.E. | z val. | p |
| (Intercept) | -0.5226 | 0.1979 | -2.6411 | 0.0083 |
| LMI\_compreh | 0.4287 | 0.0755 | 5.6789 | 0.0000 |
| Age\_cohort.z | 0.7557 | 0.0763 | 9.9101 | 0.0000 |
| Sex.z | 0.1665 | 0.0772 | 2.1553 | 0.0311 |
| Edu\_bkgr\_2cat.z | 0.0922 | 0.0825 | 1.1178 | 0.2637 |
| Age\_cohort.z:Sex.z | 0.2526 | 0.1555 | 1.6243 | 0.1043 |

| Random Effects | | |
| --- | --- | --- |
| Group | Parameter | Std. Dev. |
| UID | (Intercept) | 0.4634 |
| Item | (Intercept) | 0.6129 |

| Grouping Variables | | |
| --- | --- | --- |
| Group | # groups | ICC |
| UID | 500 | 0.0553 |
| Item | 10 | 0.0968 |

```
plot(allEffects(compreh.model2_sexage))
```

#### 4.3.2.4 LMID (cohort-based prototype)

```
cohort.model2_sexage <- glmer(Change ~ LMI_cohort +Age_cohort.z + Sex.z +Edu_bkgr_2cat.z +
                        Sex.z:Age_cohort.z +
                  (1|UID) + (1|Item), 
                data=compara, family=binomial(),
                control = glmerControl(optimizer = "bobyqa"))

summ(cohort.model2_sexage, digits=4)
```

|  |  |
| --- | --- |
| Observations | 4983 |
| Dependent variable | Change |
| Type | Mixed effects generalized linear model |
| Family | binomial |
| Link | logit |

|  |  |
| --- | --- |
| AIC | 6115.5107 |
| BIC | 6167.6210 |
| Pseudo-R² (fixed effects) | 0.0530 |
| Pseudo-R² (total) | 0.1958 |

| Fixed Effects | | | | |
| --- | --- | --- | --- | --- |
|  | Est. | S.E. | z val. | p |
| (Intercept) | -0.5234 | 0.1978 | -2.6454 | 0.0082 |
| LMI\_cohort | 0.4643 | 0.0770 | 6.0308 | 0.0000 |
| Age\_cohort.z | 0.6896 | 0.0772 | 8.9346 | 0.0000 |
| Sex.z | 0.1743 | 0.0769 | 2.2678 | 0.0233 |
| Edu\_bkgr\_2cat.z | 0.0989 | 0.0822 | 1.2035 | 0.2288 |
| Age\_cohort.z:Sex.z | 0.2261 | 0.1546 | 1.4625 | 0.1436 |

| Random Effects | | |
| --- | --- | --- |
| Group | Parameter | Std. Dev. |
| UID | (Intercept) | 0.4571 |
| Item | (Intercept) | 0.6128 |

| Grouping Variables | | |
| --- | --- | --- |
| Group | # groups | ICC |
| UID | 500 | 0.0539 |
| Item | 10 | 0.0969 |

```
plot(allEffects(cohort.model2_sexage))
```

#### 4.3.2.5 Without LMI and Education

```
model2_sexage <- glmer(Change ~ Age_cohort.z + Sex.z +
                        Sex.z:Age_cohort.z +
                  (1|UID) + (1|Item), 
                data=compara, family=binomial(),
                control = glmerControl(optimizer = "bobyqa"))

summ(model2_sexage, digits=4)
```

|  |  |
| --- | --- |
| Observations | 4983 |
| Dependent variable | Change |
| Type | Mixed effects generalized linear model |
| Family | binomial |
| Link | logit |

|  |  |
| --- | --- |
| AIC | 6147.9030 |
| BIC | 6186.9857 |
| Pseudo-R² (fixed effects) | 0.0402 |
| Pseudo-R² (total) | 0.1955 |

| Fixed Effects | | | | |
| --- | --- | --- | --- | --- |
|  | Est. | S.E. | z val. | p |
| (Intercept) | -0.5099 | 0.1977 | -2.5790 | 0.0099 |
| Age\_cohort.z | 0.7848 | 0.0783 | 10.0222 | 0.0000 |
| Sex.z | 0.1718 | 0.0778 | 2.2083 | 0.0272 |
| Age\_cohort.z:Sex.z | 0.2254 | 0.1555 | 1.4492 | 0.1473 |

| Random Effects | | |
| --- | --- | --- |
| Group | Parameter | Std. Dev. |
| UID | (Intercept) | 0.5094 |
| Item | (Intercept) | 0.6127 |

| Grouping Variables | | |
| --- | --- | --- |
| Group | # groups | ICC |
| UID | 500 | 0.0661 |
| Item | 10 | 0.0957 |

```
plot(allEffects(model2_sexage))
```

### 4.3.3 Miscellanea: Dredging experiment to see if all fixed effects are needed in the model

We set up a model with interaction terms between LMI prototypes and
all design variables and test if there are superfluous fixed effects in
the model by running all possible models and testing their quality based
on *AICc*.

For each of the full model variants, using the `MuMIn`
package, we ranked submodels of the full model, which we extended with
interaction terms between the LMI and the other fixed effects. In the
case of LMIA and LMID, several submodels not containing the fixed effect
for education background are similar \(\Delta\)*AIC* < 2) to the full
model reported before. But this submodel ranking based on *AIC*
also shows that involving educational background in the model does not
change the model quality significantly, which means that its involvement
is not superfluous either; thus, the full model, involving all survey
design variables, is also of high quality.

#### 4.3.3.1 LMIA

```
mm <- glmer(Change ~  LMI_minimal +Age_cohort.z + Sex.z + Edu_bkgr_2cat.z +
            Age_cohort.z:LMI_minimal + 
            Sex.z:LMI_minimal + 
            Edu_bkgr_2cat.z:LMI_minimal + 
              (1|UID) + (1|Item),
          data=compara, family=binomial(), na.action=na.fail,
          control = glmerControl(optimizer = "bobyqa"))

# run all models (rank them using AICc) takes ~ 1 minute
ms<- dredge(mm, rank="AICc")

subset(ms, delta < 4) # this shows the models that have a delta (difference between AICc) smaller than 4, which means they are very similar to each other
```

```
## Global model call: glmer(formula = Change ~ LMI_minimal + Age_cohort.z + Sex.z + 
##     Edu_bkgr_2cat.z + Age_cohort.z:LMI_minimal + Sex.z:LMI_minimal + 
##     Edu_bkgr_2cat.z:LMI_minimal + (1 | UID) + (1 | Item), data = compara, 
##     family = binomial(), control = glmerControl(optimizer = "bobyqa"), 
##     na.action = na.fail)
## ---
## Model selection table 
##      (Int) Age_chr.z Edu_bkg_2ct.z LMI_mnm  Sex.z Age_chr.z:LMI_mnm
## 30 -0.5057    0.7663                0.3777 0.1831           -0.3786
## 32 -0.5186    0.7716       0.09209  0.3754 0.1998           -0.3805
## 94 -0.5052    0.7668                0.3771 0.1818           -0.3709
## 96 -0.5178    0.7719       0.08979  0.3750 0.1982           -0.3736
## 64 -0.5190    0.7710       0.09115  0.3614 0.1978           -0.3749
## 22 -0.5059    0.7669                0.3742                  -0.3838
##    Edu_bkg_2ct.z:LMI_mnm LMI_mnm:Sex.z df    logLik   AICc delta weight
## 30                                      7 -3055.323 6124.7  0.00  0.347
## 32                                      8 -3054.673 6125.4  0.71  0.244
## 94                             0.08851  8 -3055.150 6126.3  1.66  0.152
## 96                             0.07950  9 -3054.533 6127.1  2.43  0.103
## 64               0.07954                9 -3054.549 6127.1  2.47  0.101
## 22                                      6 -3058.208 6128.4  3.76  0.053
## Models ranked by AICc(x) 
## Random terms (all models): 
##   1 | UID, 1 | Item
```

This shows the models that have a the difference in *AICc*
smaller than 4, which means they are very similar to each other. The
worst the difference in *AICc* is 120 so there are indeed larger
differences between models (combinations of predictors). Including all
fixed effects and no interaction terms is similar to including all fixed
effects and all interactions but the best performing model does not
include the education background while including the interaction term
between LMI\_A and Age.

#### 4.3.3.2 LMIB

```
mm <- glmer(Change ~  LMI_cumulated +Age_cohort.z + Sex.z + Edu_bkgr_2cat.z +
            Age_cohort.z:LMI_cumulated + 
            Sex.z:LMI_cumulated + 
            Edu_bkgr_2cat.z:LMI_cumulated + 
              (1|UID) + (1|Item),
          data=compara, family=binomial(), na.action=na.fail,
          control = glmerControl(optimizer = "bobyqa"))

ms<- dredge(mm, rank="AICc")

subset(ms, delta < 4)
```

```
## Global model call: glmer(formula = Change ~ LMI_cumulated + Age_cohort.z + Sex.z + 
##     Edu_bkgr_2cat.z + Age_cohort.z:LMI_cumulated + Sex.z:LMI_cumulated + 
##     Edu_bkgr_2cat.z:LMI_cumulated + (1 | UID) + (1 | Item), data = compara, 
##     family = binomial(), control = glmerControl(optimizer = "bobyqa"), 
##     na.action = na.fail)
## ---
## Model selection table 
##       (Int) Age_chr.z Edu_bkg_2ct.z LMI_cml  Sex.z Age_chr.z:LMI_cml
## 32  -0.5113    0.6599        0.1399  0.4807 0.1803           -0.2738
## 96  -0.5122    0.6663        0.1305  0.4791 0.1759           -0.2535
## 16  -0.5291    0.6681        0.1374  0.4395 0.1919                  
## 30  -0.4920    0.6555                0.4689 0.1552           -0.2685
## 80  -0.5286    0.6748        0.1268  0.4413 0.1864                  
## 94  -0.4946    0.6631                0.4678 0.1522           -0.2454
## 78  -0.5110    0.6715                0.4314 0.1630                  
## 14  -0.5099    0.6636                0.4286 0.1670                  
## 64  -0.5111    0.6599        0.1396  0.4800 0.1800           -0.2735
## 128 -0.5116    0.6663        0.1290  0.4751 0.1747           -0.2514
## 48  -0.5282    0.6681        0.1358  0.4350 0.1903                  
## 112 -0.5271    0.6748        0.1237  0.4338 0.1836                  
## 22  -0.4900    0.6533                0.4781                  -0.3004
## 24  -0.5043    0.6565        0.1049  0.4881                  -0.3084
##     Edu_bkg_2ct.z:LMI_cml LMI_cml:Sex.z df    logLik   AICc delta weight
## 32                                       8 -3051.487 6119.0  0.00  0.155
## 96                               0.1728  9 -3050.842 6119.7  0.72  0.108
## 16                                       7 -3052.882 6119.8  0.78  0.104
## 30                                       7 -3052.993 6120.0  1.01  0.093
## 80                               0.1986  8 -3052.026 6120.1  1.08  0.090
## 94                               0.1980  8 -3052.142 6120.3  1.31  0.080
## 78                               0.2225  7 -3053.248 6120.5  1.52  0.072
## 14                                       6 -3054.329 6120.7  1.67  0.067
## 64               0.004196                9 -3051.486 6121.0  2.01  0.057
## 128              0.021860        0.1748 10 -3050.833 6121.7  2.71  0.040
## 48               0.026910                8 -3052.868 6121.8  2.76  0.039
## 112              0.045250        0.2023  9 -3051.988 6122.0  3.01  0.034
## 22                                       6 -3055.069 6122.2  3.15  0.032
## 24                                       7 -3054.200 6122.4  3.42  0.028
## Models ranked by AICc(x) 
## Random terms (all models): 
##   1 | UID, 1 | Item
```

Here, actually the best model includes the fixed effects and the
interaction with Age\_cohort (nr.32), but the one that includes all fixed
effects without interactions (nr. 16) has a very small *AICc*
difference to the top one. Several models with the difference in
*AICc* < 4 do not include all fixed effects.

#### 4.3.3.3 LMIC

```
mm <- glmer(Change ~  LMI_compreh +Age_cohort.z + Sex.z + Edu_bkgr_2cat.z +
            Age_cohort.z:LMI_compreh + 
            Sex.z:LMI_compreh + 
            Edu_bkgr_2cat.z:LMI_compreh + 
              (1|UID) + (1|Item),
          data=compara, family=binomial(), na.action=na.fail,
          control = glmerControl(optimizer = "bobyqa"))

ms<- dredge(mm, rank="AICc")

subset(ms, delta < 4)
```

```
## Global model call: glmer(formula = Change ~ LMI_compreh + Age_cohort.z + Sex.z + 
##     Edu_bkgr_2cat.z + Age_cohort.z:LMI_compreh + Sex.z:LMI_compreh + 
##     Edu_bkgr_2cat.z:LMI_compreh + (1 | UID) + (1 | Item), data = compara, 
##     family = binomial(), control = glmerControl(optimizer = "bobyqa"), 
##     na.action = na.fail)
## ---
## Model selection table 
##       (Int) Age_chr.z Edu_bkg_2ct.z LMI_cmp  Sex.z Age_chr.z:LMI_cmp
## 16  -0.5275    0.7596       0.12280  0.4203 0.1797                  
## 32  -0.5250    0.7613       0.12660  0.4368 0.1716           -0.2039
## 14  -0.5103    0.7533                0.4145 0.1575                  
## 30  -0.5074    0.7547                0.4302 0.1492           -0.1952
## 48  -0.5260    0.7581       0.12000  0.4095 0.1779                  
## 80  -0.5280    0.7618       0.12150  0.4186 0.1787                  
## 64  -0.5239    0.7600       0.12440  0.4283 0.1705           -0.2000
## 78  -0.5111    0.7560                0.4126 0.1567                  
## 96  -0.5254    0.7626       0.12580  0.4354 0.1711           -0.2002
## 22  -0.5072    0.7543                0.4397                  -0.2209
## 6   -0.5105    0.7527                0.4223                         
## 94  -0.5081    0.7566                0.4285 0.1487           -0.1904
## 24  -0.5202    0.7593       0.09374  0.4456                  -0.2303
## 8   -0.5228    0.7573       0.08752  0.4272                         
## 112 -0.5263    0.7605       0.11810  0.4059 0.1766                  
##     Edu_bkg_2ct.z:LMI_cmp LMI_cmp:Sex.z df    logLik   AICc delta weight
## 16                                       7 -3053.092 6120.2  0.00  0.150
## 32                                       8 -3052.200 6120.4  0.22  0.134
## 14                                       6 -3054.251 6120.5  0.31  0.128
## 30                                       7 -3053.436 6120.9  0.69  0.106
## 48                0.06793                8 -3053.001 6122.0  1.82  0.060
## 80                              0.05574  8 -3053.024 6122.1  1.87  0.059
## 64                0.05121                9 -3052.149 6122.3  2.13  0.052
## 78                              0.06559  7 -3054.157 6122.3  2.13  0.052
## 96                              0.03538  9 -3052.173 6122.4  2.18  0.051
## 22                                       6 -3055.350 6122.7  2.51  0.043
## 6                                        5 -3056.391 6122.8  2.59  0.041
## 94                              0.04629  8 -3053.390 6122.8  2.60  0.041
## 24                                       7 -3054.656 6123.3  3.13  0.031
## 8                                        6 -3055.788 6123.6  3.39  0.028
## 112               0.07802       0.06643  9 -3052.907 6123.8  3.64  0.024
## Models ranked by AICc(x) 
## Random terms (all models): 
##   1 | UID, 1 | Item
```

The model with all fixed effects tops the chart, followed by all
fixed effects plus interaction with age cohorts coming in at the second
place. Similarly to the above calculations, several models with the
difference in *AICc* < 4 do not include all fixed effects.

#### 4.3.3.4 LMID

```
mm <- glmer(Change ~  LMI_cohort +Age_cohort.z + Sex.z + Edu_bkgr_2cat.z +
            Age_cohort.z:LMI_cohort + 
            Sex.z:LMI_cohort + 
            Edu_bkgr_2cat.z:LMI_cohort + 
              (1|UID) + (1|Item),
          data=compara, family=binomial(), na.action=na.fail,
          control = glmerControl(optimizer = "bobyqa"))

ms<- dredge(mm, rank="AICc")

subset(ms, delta < 4)
```

```
## Global model call: glmer(formula = Change ~ LMI_cohort + Age_cohort.z + Sex.z + 
##     Edu_bkgr_2cat.z + Age_cohort.z:LMI_cohort + Sex.z:LMI_cohort + 
##     Edu_bkgr_2cat.z:LMI_cohort + (1 | UID) + (1 | Item), data = compara, 
##     family = binomial(), control = glmerControl(optimizer = "bobyqa"), 
##     na.action = na.fail)
## ---
## Model selection table 
##       (Int) Age_chr.z Edu_bkg_2ct.z LMI_chr  Sex.z Age_chr.z:LMI_chr
## 32  -0.5154    0.6907       0.12430  0.4886 0.1785           -0.2496
## 30  -0.4977    0.6851                0.4824 0.1561           -0.2535
## 16  -0.5278    0.6934       0.12640  0.4600 0.1859                  
## 14  -0.5100    0.6877                0.4533 0.1631                  
## 96  -0.5158    0.6928       0.12160  0.4863 0.1766           -0.2437
## 64  -0.5141    0.6906       0.12160  0.4826 0.1765           -0.2478
## 94  -0.4987    0.6877                0.4798 0.1544           -0.2462
## 80  -0.5279    0.6959       0.12300  0.4581 0.1836                  
## 48  -0.5262    0.6932       0.12360  0.4535 0.1834                  
## 78  -0.5108    0.6908                0.4512 0.1611                  
## 22  -0.4969    0.6838                0.4901                  -0.2725
## 24  -0.5096    0.6879       0.08925  0.4955                  -0.2718
## 128 -0.5142    0.6929       0.11760  0.4780 0.1735           -0.2403
## 6   -0.5102    0.6866                0.4590                         
## 112 -0.5259    0.6960       0.11860  0.4492 0.1800                  
##     Edu_bkg_2ct.z:LMI_chr LMI_chr:Sex.z df    logLik   AICc delta weight
## 32                                       8 -3049.566 6115.2  0.00  0.159
## 30                                       7 -3050.769 6115.6  0.40  0.130
## 16                                       7 -3050.817 6115.7  0.50  0.124
## 14                                       6 -3052.055 6116.1  0.97  0.098
## 96                              0.08056  9 -3049.424 6116.9  1.72  0.067
## 64                0.05752                9 -3049.496 6117.0  1.87  0.062
## 94                              0.09532  8 -3050.570 6117.2  2.01  0.058
## 80                              0.09740  8 -3050.610 6117.2  2.09  0.056
## 48                0.06468                8 -3050.730 6117.5  2.33  0.050
## 78                              0.11300  7 -3051.777 6117.6  2.42  0.047
## 22                                       6 -3052.897 6117.8  2.65  0.042
## 24                                       7 -3052.261 6118.5  3.38  0.029
## 128               0.07511       0.09401 10 -3049.310 6118.7  3.50  0.028
## 6                                        5 -3054.374 6118.8  3.60  0.026
## 112               0.08560       0.11260  9 -3050.462 6119.0  3.80  0.024
## Models ranked by AICc(x) 
## Random terms (all models): 
##   1 | UID, 1 | Item
```

Here, actually the best model includes the fixed effects and the
interaction with Age\_cohort, but the one that includes all fixed effects
without interactions has a very small *AICc* difference to the
top one and comes at third place. Not all models with the difference in
*AICc* < 4 include all design variables. Educational
background often becomes superfluous and sometimes sex as well. LMI\_D,
however, is always in the model.

Because the model qualities in all cases are similar with or without
interactions, we did not include further interactions with the LMI
prototypes.

# 5 Spatial characteristics in a table

The following table shows all 125 SDATS localities with their average
dialect change rates and average LMI values, by the two age cohorts.
This data was used for the creation the maps in Fig 5 in the
manuscript.

```
library(DT)
library(kableExtra)
ling_df <- read.csv("sdats125_change_LMI.csv", header =T, stringsAsFactors = F)
kbl(ling_df, digits = 4) %>%
  kable_paper() %>%
  scroll_box(width = "1000px", height = "400px")
```

| X | SDATS\_fullname | site\_code | canton | Population2018 | avg\_dialChange\_old | avg\_dialChange\_young | avg\_LMI\_A\_old | avg\_LMI\_A\_young | avg\_LMI\_B\_old | avg\_LMI\_B\_young | avg\_LMI\_C\_old | avg\_LMI\_C\_young | avg\_LMI\_D\_old | avg\_LMI\_D\_young |
| --- | --- | --- | --- | --- | --- | --- | --- | --- | --- | --- | --- | --- | --- | --- |
| 1 | Aarau | AG01 | AG | 21506 | 0.1500 | 0.4500 | -0.4016 | 0.0052 | -0.4192 | -0.0670 | -0.4654 | -0.1298 | -0.4486 | -0.1155 |
| 2 | Bremgarten/Widen/Zufikon | AG02 | AG | 7946 | 0.4500 | 0.5500 | -0.1843 | -0.1021 | 0.0733 | 0.0452 | 0.0245 | -0.2159 | -0.5537 | -0.1898 |
| 3 | Brugg | AG03 | AG | 11179 | 0.1500 | 0.3500 | 0.3391 | 0.2968 | 0.2432 | 0.2489 | 0.4156 | 0.2033 | 0.3608 | 0.1937 |
| 4 | Frick | AG04 | AG | 5564 | 0.5500 | 0.7500 | -0.4016 | 0.8277 | -0.2644 | 0.8270 | -0.1944 | 0.7187 | -0.1898 | 0.6563 |
| 5 | Gränichen | AG05 | AG | 7735 | 0.1000 | 0.4000 | -0.4016 | 0.0086 | -0.4412 | 0.1268 | -0.3684 | -0.0049 | -0.3483 | 0.0968 |
| 6 | Menziken | AG06 | AG | 6334 | 0.2000 | 0.6500 | -0.4016 | -0.3661 | -0.5139 | -0.5133 | -0.4594 | -0.4610 | -0.4346 | -0.4084 |
| 7 | Merenschwand | AG07 | AG | 3549 | 0.5500 | 0.5500 | 0.1681 | -0.4016 | -0.0976 | 0.0796 | -0.0264 | -0.1485 | -0.0364 | -0.0529 |
| 8 | Möhlin | AG08 | AG | 11043 | 0.4000 | 0.6000 | 0.1189 | -0.0790 | -0.0801 | -0.2114 | 0.0391 | -0.0472 | -0.2814 | 0.0063 |
| 9 | Spreitenbach | AG09 | AG | 11881 | 0.6000 | 0.7444 | 0.4305 | -0.0417 | 0.0182 | 0.4637 | 0.1873 | 0.0111 | 0.1665 | 0.0622 |
| 10 | Würenlingen | AG10 | AG | 4702 | 0.2500 | 0.7000 | -0.1411 | -0.2926 | -0.2539 | 0.0908 | -0.1972 | -0.0640 | -0.4500 | 0.0371 |
| 11 | Zofingen | AG11 | AG | 11656 | 0.1500 | 0.5000 | -0.3159 | -0.4016 | -0.1684 | -0.2536 | -0.0360 | -0.2294 | -0.0394 | -0.1426 |
| 12 | Appenzell | AI01 | AI | 5795 | 0.4000 | 0.4500 | 0.2661 | 0.2809 | -0.0685 | 0.8166 | 0.0661 | 0.4412 | 0.0469 | 0.5433 |
| 13 | Herisau | AR01 | AR | 15745 | 0.3500 | 0.4000 | -0.0517 | -0.2027 | -0.1289 | -0.0941 | -0.0543 | -0.2578 | -0.0895 | -0.2239 |
| 14 | Teufen | AR02 | AR | 6265 | 0.3500 | 0.2000 | 0.0326 | -0.0709 | -0.0030 | 0.2239 | 0.0312 | -0.2206 | -0.2060 | -0.0738 |
| 15 | Aarberg | BE01 | BE | 4628 | 0.1000 | 0.2500 | -0.3240 | -0.1208 | -0.4057 | -0.1650 | -0.3249 | -0.2031 | -0.4415 | -0.1828 |
| 16 | Adelboden | BE02 | BE | 3390 | 0.5000 | 0.6000 | -0.2710 | -0.4016 | -0.2977 | -0.2308 | -0.2415 | -0.5360 | -0.2335 | -0.4553 |
| 17 | Bern | BE03 | BE | 133883 | 0.2000 | 0.2000 | -0.1140 | 0.0920 | -0.2319 | -0.1826 | -0.1752 | -0.0941 | -0.2740 | -0.0929 |
| 18 | Biel | BE04 | BE | 55159 | 0.0000 | 0.0000 | -0.1361 | -0.4016 | -0.2643 | -0.0552 | -0.1560 | -0.4554 | -0.1536 | -0.3888 |
| 19 | Blumenstein | BE05 | BE | 1246 | 0.1000 | 0.2000 | -0.1344 | -0.2949 | -0.2018 | -0.2264 | -0.0836 | -0.3551 | -0.3927 | -0.2521 |
| 20 | Büren | BE06 | BE | 3583 | 0.1500 | 0.2500 | 0.0474 | -0.2720 | -0.0594 | -0.0266 | 0.0664 | -0.2865 | -0.3943 | -0.2411 |
| 21 | Burgdorf | BE07 | BE | 16420 | 0.1000 | 0.4000 | -0.2338 | 0.7256 | -0.3210 | 0.7678 | -0.2213 | 0.7358 | -0.2132 | 0.7895 |
| 22 | Spiez/Faulensee | BE08 | BE | 12812 | 0.1000 | 0.3000 | 0.2322 | -0.1378 | 0.0584 | 0.1446 | 0.2336 | -0.1352 | 0.2068 | -0.0509 |
| 23 | Frutigen | BE09 | BE | 6923 | 0.2000 | 0.4000 | -0.4016 | 0.4993 | -0.5423 | 0.3478 | -0.4916 | 0.3479 | -0.4628 | 0.4032 |
| 24 | Grindelwald | BE10 | BE | 3802 | 0.3500 | 0.3500 | 0.9538 | -0.4016 | 0.6066 | 0.0476 | 0.8115 | -0.3447 | 0.7170 | -0.2060 |
| 25 | Huttwil | BE11 | BE | 4898 | 0.1500 | 0.2500 | -0.2492 | 0.4930 | -0.3819 | 0.1747 | -0.3522 | 0.2650 | -0.3720 | 0.3254 |
| 26 | Ins | BE12 | BE | 3564 | 0.2500 | 0.2500 | 0.4989 | 0.4569 | 0.1761 | 0.1078 | 0.3574 | 0.2274 | 0.3177 | 0.2034 |
| 27 | Konolfingen | BE13 | BE | 5283 | 0.0500 | 0.3000 | -0.2366 | 1.3775 | -0.2393 | 0.9745 | -0.1272 | 1.1367 | -0.3988 | 1.0648 |
| 28 | Langenthal | BE14 | BE | 15624 | 0.2000 | 0.3000 | -0.0667 | 0.4044 | -0.0105 | 1.2495 | 0.1357 | 0.8073 | 0.1078 | 0.8482 |
| 29 | Langnau | BE15 | BE | 9380 | 0.0500 | 0.2500 | 0.0489 | -0.1490 | -0.1273 | -0.1898 | 0.0055 | -0.2188 | -0.3655 | -0.1840 |
| 30 | Lauterbrunnen | BE16 | BE | 2290 | 0.2000 | 0.7500 | -0.4016 | 0.4075 | -0.5956 | 0.4128 | -0.5475 | 0.5376 | -0.5108 | 0.6989 |
| 31 | Meiringen | BE17 | BE | 4622 | 0.2000 | 0.4500 | -0.4016 | -0.2553 | -0.3120 | -0.2697 | -0.2080 | -0.3234 | -0.1979 | -0.2024 |
| 32 | Münchenbuchsee | BE18 | BE | 10213 | 0.1000 | 0.3000 | -0.1916 | -0.2814 | -0.3880 | -0.1323 | -0.3012 | -0.1714 | -0.2848 | 0.0123 |
| 33 | Niederbipp | BE19 | BE | 4715 | 0.4000 | 0.5000 | 0.4915 | -0.2332 | 0.2724 | 0.3365 | 0.3724 | 0.0566 | 0.3295 | 0.1096 |
| 34 | Saanen/Gstaad | BE20 | BE | 6818 | 0.2000 | 0.1500 | -0.4016 | -0.3341 | 0.3211 | -0.5133 | 0.5075 | -0.4688 | -0.6084 | -0.3639 |
| 35 | Schwarzenburg | BE21 | BE | 6831 | 0.2111 | 0.3000 | -0.1989 | 0.5284 | -0.4461 | 0.5612 | -0.3686 | 0.4692 | -0.3459 | 0.5354 |
| 36 | Sumiswald | BE22 | BE | 5045 | 0.1000 | 0.2000 | -0.2620 | -0.0610 | -0.1631 | 0.3255 | -0.0701 | 0.0886 | -0.0731 | 0.1733 |
| 37 | Thun | BE23 | BE | 43734 | 0.0500 | 0.2000 | -0.2808 | 0.2948 | -0.4511 | 0.0746 | -0.4089 | 0.1604 | -0.3935 | 0.1552 |
| 38 | Interlaken/Unterseen | BE24 | BE | 5751 | 0.3000 | 0.4000 | -0.0172 | 0.0184 | 0.0941 | -0.2576 | 0.2145 | -0.1686 | 0.1783 | -0.1303 |
| 39 | Zweisimmen | BE25 | BE | 3018 | 0.2667 | 0.3000 | -0.2831 | -0.1056 | -0.4991 | -0.0420 | -0.4413 | -0.1658 | -0.4135 | -0.0229 |
| 40 | Aesch | BL01 | BL | 10352 | 0.3000 | 0.5500 | 0.2731 | 1.1445 | 0.2878 | 1.0234 | 0.3929 | 0.9496 | 0.2203 | 0.8855 |
| 41 | Gelterkinden | BL02 | BL | 6200 | 0.2000 | 0.5000 | -0.0542 | -0.2265 | 0.1205 | -0.1811 | 0.2419 | -0.2171 | 0.2083 | -0.1560 |
| 42 | Laufen | BL03 | BL | 5619 | 0.6500 | 0.7000 | 0.9466 | 0.3039 | 0.6148 | 0.7086 | 0.8685 | 0.4497 | 0.4218 | 0.6158 |
| 43 | Liestal | BL04 | BL | 14390 | 0.2000 | 0.5000 | 0.1115 | -0.1039 | -0.1193 | -0.0469 | -0.0229 | -0.0792 | -0.1664 | -0.0415 |
| 44 | Reigoldswil | BL05 | BL | 1528 | 0.2500 | 0.4500 | -0.4016 | -0.1704 | -0.4315 | -0.0501 | -0.3691 | -0.1119 | -0.3474 | -0.0140 |
| 45 | Basel | BS01 | BS | 172258 | 0.2500 | 0.1500 | 0.9543 | 0.1790 | 0.8532 | 0.6662 | 1.0551 | 0.1336 | 0.4514 | 0.1637 |
| 46 | Düdingen | FR01 | FR | 7964 | 0.3500 | 0.4000 | 0.7078 | 0.0139 | 0.3333 | -0.1557 | 0.5242 | -0.1141 | 0.4686 | -0.1113 |
| 47 | Freiburg | FR02 | FR | 38365 | 0.5000 | 0.9000 | 0.1820 | 0.3200 | 0.2038 | 0.9322 | 0.3827 | 0.3968 | 0.3451 | 0.4800 |
| 48 | Gurmels | FR03 | FR | 4352 | 0.3000 | 0.5500 | -0.2539 | 0.2637 | -0.1382 | 0.3072 | -0.0087 | 0.3412 | -0.0156 | 0.3955 |
| 49 | Jaun | FR04 | FR | 644 | 0.2500 | 0.4500 | -0.0806 | -0.0263 | -0.2059 | -0.2902 | -0.0810 | -0.2112 | -0.0808 | -0.1813 |
| 50 | Murten | FR05 | FR | 8279 | 0.4000 | 0.4500 | 0.3857 | -0.4016 | 0.3618 | 0.2087 | 0.3019 | -0.2184 | 0.2526 | -0.1634 |
| 51 | Plaffeien | FR06 | FR | 3594 | 0.3500 | 0.4000 | -0.4016 | -0.3007 | -0.5920 | -0.3549 | -0.5441 | -0.3645 | -0.5088 | -0.2985 |
| 52 | Hintere Linthal/Auen | GL01 | GL | 361 | 0.5500 | 0.6500 | 0.4622 | -0.3592 | 0.1766 | -0.4567 | 0.2793 | -0.4275 | 0.2457 | -0.3700 |
| 53 | Glarus | GL02 | GL | 12426 | 0.3000 | 0.4500 | -0.0477 | -0.3611 | -0.2854 | -0.0876 | -0.2052 | -0.2451 | -0.2051 | -0.1910 |
| 54 | Chur/Masans | GR01 | GR | 35378 | 0.4000 | 0.4500 | 0.9412 | -0.2039 | 0.4336 | -0.2619 | 0.6543 | -0.2811 | 0.5917 | -0.0294 |
| 55 | Churwalden | GR02 | GR | 1949 | 0.3111 | 0.5000 | 0.2809 | 1.1453 | 0.2570 | 0.9684 | 0.4732 | 0.8959 | 0.4300 | 0.8480 |
| 56 | Davos/Frauenkirch | GR03 | GR | 10899 | 0.3000 | 0.6000 | -0.2888 | 0.3078 | -0.3765 | 0.5764 | -0.2853 | 0.4450 | -0.2780 | 0.5203 |
| 57 | Igis | GR04 | GR | 3487 | 0.3500 | 0.6000 | -0.1983 | 0.2305 | -0.3609 | 0.1464 | -0.2724 | 0.2761 | -0.2599 | 0.3604 |
| 58 | Obersaxen | GR05 | GR | 1188 | 0.5500 | 0.7000 | -0.4016 | 0.3328 | -0.2726 | 1.0960 | -0.1608 | 0.6535 | -0.1544 | 0.8549 |
| 59 | Schiers | GR06 | GR | 2679 | 0.5000 | 0.5500 | -0.1109 | 0.6348 | 0.0460 | 0.5507 | 0.1293 | 0.7544 | 0.1033 | 0.7642 |
| 60 | Schmitten | GR07 | GR | 234 | 0.3167 | 0.5000 | -0.4016 | 0.6512 | 0.1972 | 1.4017 | 0.2975 | 0.9034 | 0.2190 | 1.0641 |
| 61 | Thusis | GR08 | GR | 3217 | 0.4000 | 0.3000 | -0.1701 | 0.7174 | -0.3505 | 0.5182 | -0.2829 | 0.6302 | -0.2867 | 0.6617 |
| 62 | Vals | GR09 | GR | 1007 | 0.3000 | 0.5000 | -0.4016 | 0.2862 | -0.2455 | 0.7008 | -0.1847 | 0.9046 | -0.1953 | 1.3018 |
| 63 | Escholzmatt | LU01 | LU | 4342 | 0.3500 | 0.6333 | -0.3342 | -0.4016 | -0.4584 | -0.1309 | -0.4232 | -0.3277 | -0.4069 | -0.2293 |
| 64 | Luzern | LU02 | LU | 81691 | 0.6000 | 0.7333 | 0.3714 | -0.3351 | 0.1553 | -0.2698 | 0.3450 | -0.4318 | 0.3089 | -0.4042 |
| 65 | Marbach | LU03 | LU | 4342 | 0.6000 | 0.5000 | -0.2896 | -0.3323 | -0.3114 | -0.2401 | -0.2073 | -0.3156 | -0.1972 | -0.2716 |
| 66 | Sursee | LU04 | LU | 9955 | 0.3000 | 0.5500 | -0.1769 | -0.4016 | -0.2849 | -0.2438 | -0.1865 | -0.3519 | -0.1831 | -0.2893 |
| 67 | Weggis | LU05 | LU | 4369 | 0.6000 | 0.8500 | 0.9402 | 0.2115 | 0.5169 | 0.6418 | 0.7770 | 0.4009 | 0.7088 | 0.4255 |
| 68 | Willisau | LU06 | LU | 7781 | 0.4500 | 0.6000 | -0.1772 | -0.3213 | -0.3523 | 0.4538 | -0.2646 | -0.0805 | -0.2596 | 0.0742 |
| 69 | Stans/Oberdorf | NW01 | NW | 8393 | 0.3500 | 0.5500 | -0.2897 | -0.4016 | -0.2294 | 0.0013 | -0.1092 | -0.3725 | -0.1068 | -0.3225 |
| 70 | Engelberg | OW01 | OW | 4181 | 0.3500 | 0.5500 | -0.1968 | -0.4016 | -0.1086 | -0.0486 | 0.0131 | -0.2317 | -0.0016 | -0.1328 |
| 71 | Lungern | OW02 | OW | 2120 | 0.2500 | 0.4500 | -0.4016 | -0.2729 | -0.5248 | 0.2584 | -0.5252 | 0.0546 | -0.4976 | 0.1747 |
| 72 | Sarnen-Kägiswil | OW03 | OW | 10368 | 0.6000 | 0.8000 | 0.1531 | 0.2986 | 0.2411 | 0.3435 | 0.4009 | 0.3094 | 0.3382 | 0.3396 |
| 73 | Diepoldsau | SG01 | SG | 6471 | 0.3000 | 0.5000 | -0.4016 | -0.0982 | -0.5386 | 0.0606 | -0.5077 | -0.0405 | -0.4872 | 0.0514 |
| 74 | Ebnat-Kappel | SG02 | SG | 5031 | 0.4500 | 0.4000 | -0.0886 | -0.0542 | -0.2811 | -0.0803 | -0.1726 | -0.0387 | -0.1740 | 0.0810 |
| 75 | Eschenbach | SG03 | SG | 9605 | 0.3500 | 0.7500 | 0.2218 | 0.2144 | 0.0885 | 0.2357 | 0.2714 | 0.1910 | 0.2440 | 0.2118 |
| 76 | Flawil | SG04 | SG | 10523 | 0.4500 | 0.6000 | 0.3082 | -0.3102 | -0.0244 | -0.3498 | 0.0963 | -0.3601 | 0.0770 | -0.3253 |
| 77 | Grabs | SG05 | SG | 7068 | 0.4000 | 0.3500 | 0.3039 | 0.3058 | 0.1539 | 0.6170 | 0.3497 | 0.1886 | 0.3162 | 0.2331 |
| 78 | Mels | SG06 | SG | 8623 | 0.2500 | 0.3000 | -0.2610 | -0.0913 | -0.2364 | 0.0271 | -0.0164 | 0.1524 | -1.0545 | 0.2269 |
| 79 | Oberriet | SG07 | SG | 8879 | 0.4500 | 0.6000 | -0.4016 | 0.6231 | -0.4404 | 0.5933 | -0.3617 | 0.6125 | -0.3395 | 0.5977 |
| 80 | Rorschach/Rorschacherberg | SG08 | SG | 9441 | 0.5000 | 0.4500 | 0.3839 | -0.0485 | 0.0475 | 0.1670 | 0.2076 | -0.0983 | 0.1835 | -0.0212 |
| 81 | Schänis | SG09 | SG | 3819 | 0.2500 | 0.6500 | 0.4919 | 0.0986 | 0.2055 | 0.9604 | 0.3909 | 0.2926 | 0.3472 | 0.4227 |
| 82 | St. Gallen | SG10 | SG | 75833 | 0.2500 | 0.3500 | 0.2987 | 0.7501 | 0.2840 | 0.5903 | 0.4483 | 0.6569 | 0.3946 | 0.6764 |
| 83 | Vättis | SG11 | SG | 1565 | 0.5500 | 0.6500 | 0.3995 | 0.2314 | 0.4535 | 0.2893 | 0.4071 | 0.3246 | 0.3548 | 0.3726 |
| 84 | Wil | SG12 | SG | 23966 | 0.4500 | 0.4500 | -0.1734 | -0.4016 | -0.0012 | -0.2795 | 0.1172 | -0.4337 | 0.0858 | -0.3290 |
| 85 | Schaffhausen | SH01 | SH | 36587 | 0.2500 | 0.4000 | 0.0423 | -0.4016 | 0.0426 | -0.0484 | 0.2165 | -0.3471 | 0.1934 | -0.2646 |
| 86 | Wilchingen | SH02 | SH | 1737 | 0.6000 | 0.6333 | -0.0602 | 0.3490 | -0.2577 | 0.0662 | -0.1688 | 0.2043 | -0.1659 | 0.2543 |
| 87 | Bettlach | SO01 | SO | 4911 | 0.4000 | 0.4000 | 0.0298 | -0.2036 | -0.2582 | -0.2927 | -0.1590 | -0.2708 | -0.1552 | -0.2385 |
| 88 | Oensingen | SO02 | SO | 6288 | 0.3000 | 0.3000 | -0.2350 | 0.3281 | -0.3793 | 0.1879 | -0.2922 | 0.1902 | -0.2760 | 0.2165 |
| 89 | Olten | SO03 | SO | 18363 | 0.2500 | 0.5000 | -0.0863 | -0.4016 | -0.3630 | -0.1176 | -0.2691 | -0.3058 | -0.2542 | -0.2255 |
| 90 | Solothurn | SO04 | SO | 16777 | 0.2000 | 0.3000 | -0.1820 | -0.2513 | -0.3396 | -0.1816 | -0.2446 | -0.2568 | -0.2360 | -0.2428 |
| 91 | Einsiedeln | SZ01 | SZ | 15870 | 0.3500 | 0.6500 | -0.0908 | 0.1040 | -0.3359 | 0.4450 | -0.2670 | 0.2966 | -0.2543 | 0.4200 |
| 92 | Innerthal | SZ02 | SZ | 173 | 0.5000 | 0.6500 | -0.0123 | -0.2708 | 0.0148 | -0.1523 | 0.0482 | -0.2623 | -0.5051 | -0.2179 |
| 93 | Muotathal | SZ03 | SZ | 3527 | 0.1000 | 0.2000 | -0.4016 | -0.1854 | -0.4455 | -0.0979 | -0.3694 | -0.1926 | -0.3473 | -0.1450 |
| 94 | Schwyz | SZ04 | SZ | 15181 | 0.2500 | 0.4000 | -0.1164 | 0.1080 | -0.2927 | 0.0955 | -0.2443 | 0.1083 | -0.2482 | 0.1407 |
| 95 | Wollerau/Freienbach | SZ05 | SZ | 7255 | 0.5500 | 0.6000 | 0.4698 | -0.1320 | 0.0625 | -0.1308 | 0.2385 | -0.1958 | 0.2083 | -0.1689 |
| 96 | Amriswil | TG01 | TG | 13538 | 0.5000 | 0.5500 | 0.0607 | -0.2113 | -0.1321 | -0.2807 | -0.0223 | -0.2771 | -0.0315 | -0.2525 |
| 97 | Ermatingen | TG02 | TG | 3455 | 0.3000 | 0.5000 | -0.4016 | 0.9693 | -0.4865 | 0.8029 | -0.5048 | 0.7996 | -0.4855 | 0.7389 |
| 98 | Fischingen | TG03 | TG | 2792 | 0.3000 | 0.4500 | -0.0589 | -0.3290 | -0.0842 | -0.0414 | 0.0235 | -0.2049 | 0.0056 | -0.1173 |
| 99 | Frauenfeld | TG04 | TG | 25611 | 0.3000 | 0.4000 | 0.1884 | 0.4153 | -0.0968 | 0.3254 | 0.0418 | 0.2677 | 0.0319 | 0.2973 |
| 100 | Mammern | TG05 | TG | 654 | 0.3722 | 0.4278 | 0.0392 | -0.4016 | 0.0144 | -0.4256 | 0.0970 | -0.4322 | 0.0769 | -0.3868 |
| 101 | Weinfelden | TG06 | TG | 11534 | 0.6000 | 0.6000 | 0.5518 | 0.0347 | 0.3483 | 0.2086 | 0.5563 | 0.1416 | 0.4953 | 0.1791 |
| 102 | Bosco/Gurin | TI01 | TI | 46 | 0.1667 | 0.1000 | -0.4016 | -0.4016 | -0.5674 | -0.5648 | -0.5364 | -0.5262 | -0.5042 | -0.4082 |
| 103 | Altdorf | UR01 | UR | 9401 | 0.4000 | 0.5778 | 0.5569 | -0.3082 | 0.1397 | -0.1107 | 0.3039 | -0.2683 | 0.2723 | -0.0851 |
| 104 | Hospental | UR02 | UR | 186 | 0.4500 | 0.7000 | 0.1085 | 0.3039 | 0.0498 | 0.0637 | 0.2251 | 0.1466 | 0.2014 | 0.2026 |
| 105 | Unterschächen | UR03 | UR | 697 | 0.2500 | 0.5000 | -0.4016 | -0.2058 | -0.5169 | 0.0325 | -0.4533 | -0.1007 | -0.4240 | -0.0397 |
| 106 | Blatten | VS01 | VS | 290 | 0.2000 | 0.5500 | -0.4016 | 0.5093 | -0.4946 | 0.5701 | -0.5013 | 0.8282 | -0.5251 | 1.0031 |
| 107 | Ernen | VS02 | VS | 492 | 0.2000 | 0.6000 | -0.3254 | 0.4054 | -0.5395 | 0.6752 | -0.4803 | 0.7316 | -0.4489 | 0.9369 |
| 108 | Brig-Glis/Gamsen/Ried-Brig | VS03 | VS | 13058 | 0.1500 | 0.4000 | -0.2020 | 0.6551 | -0.4087 | 0.5389 | -0.3238 | 0.6269 | -0.3046 | 0.6744 |
| 109 | Reckingen | VS04 | VS | 405 | 0.3000 | 0.6000 | -0.0331 | -0.3451 | -0.2784 | -0.3741 | -0.1677 | -0.4243 | -0.1608 | -0.2884 |
| 110 | Saas-Grund | VS05 | VS | 1000 | 0.2500 | 0.6000 | -0.4016 | -0.4016 | -0.5956 | -0.3766 | -0.5475 | -0.4087 | -0.5108 | -0.2932 |
| 111 | Salgesch | VS06 | VS | 1548 | 0.3500 | 0.7500 | -0.0693 | -0.4016 | -0.2617 | -0.1435 | -0.1477 | -0.5205 | -0.1423 | -0.4523 |
| 112 | Simplon-Dorf | VS07 | VS | 305 | 0.3500 | 0.6500 | -0.4016 | -0.2094 | -0.4704 | 0.3403 | -0.4016 | -0.1101 | -0.3939 | 0.0731 |
| 113 | St. Niklaus | VS08 | VS | 2239 | 0.2000 | 0.4500 | -0.4016 | 0.4286 | -0.2195 | 0.3392 | -0.1324 | 0.2842 | -0.1789 | 0.3954 |
| 114 | Turtmann | VS09 | VS | 1111 | 0.2000 | 0.3000 | -0.4016 | -0.2389 | -0.5195 | -0.3042 | -0.4564 | -0.3305 | -0.4268 | -0.2927 |
| 115 | Visp | VS10 | VS | 7950 | 0.3500 | 0.3000 | 0.0592 | -0.1940 | -0.1752 | -0.2225 | -0.0847 | -0.2436 | -0.1026 | -0.1760 |
| 116 | Zermatt | VS11 | VS | 5758 | 0.1500 | 0.5500 | 0.4022 | 0.5651 | 0.2135 | 0.6186 | 0.3671 | 0.4547 | 0.3186 | 0.5545 |
| 117 | Oberägeri | ZG01 | ZG | 6081 | 0.1500 | 0.5000 | -0.0251 | -0.1190 | -0.2959 | 0.3741 | -0.2064 | 0.0299 | -0.1992 | 0.0764 |
| 118 | Zug | ZG02 | ZG | 30542 | 0.5500 | 0.5000 | 0.1558 | 0.3947 | 0.1439 | 0.2583 | 0.3092 | 0.2575 | 0.0489 | 0.3036 |
| 119 | Bauma/Saland | ZH01 | ZH | 4965 | 0.0556 | 0.4444 | 0.3039 | -0.1985 | -0.0693 | -0.3636 | 0.0796 | -0.3214 | 0.0659 | -0.3024 |
| 120 | Bülach | ZH02 | ZH | 20447 | 0.3000 | 0.3500 | -0.4016 | -0.3448 | -0.4266 | -0.4420 | -0.4462 | -0.4387 | -0.6805 | -0.3952 |
| 121 | Horgen | ZH03 | ZH | 22665 | 0.2500 | 0.4000 | 0.0382 | 0.2416 | -0.2196 | 0.1007 | -0.0990 | 0.1464 | -0.0994 | 0.1375 |
| 122 | Maur | ZH04 | ZH | 10215 | 0.2000 | 0.5500 | -0.2114 | -0.1740 | -0.4361 | -0.2485 | -0.3709 | -0.3111 | -0.3509 | -0.2555 |
| 123 | Rafz | ZH05 | ZH | 4576 | 0.3500 | 0.6500 | -0.4016 | 0.0911 | -0.5394 | 0.5813 | -0.4827 | 0.5290 | -0.4521 | 0.6732 |
| 124 | Winterthur | ZH06 | ZH | 111851 | 0.0000 | 0.3000 | -0.2968 | -0.4016 | -0.3522 | -0.3887 | -0.2561 | -0.4875 | -0.2422 | -0.4390 |
| 125 | Zürich | ZH07 | ZH | 415367 | 0.0000 | 0.3000 | -0.0895 | 0.3492 | -0.3263 | -0.0418 | -0.2398 | 0.1154 | -0.2927 | 0.1002 |

# 6 References

Baxter, G. J. and Croft, W. (2016) ‘Modeling language change across
the lifespan: Individual trajectories in community change’, *Language
Variation and Change*, 28(2), pp. 129–173. doi:
10.1017/S0954394516000077

Gelman, A., & Hill, J. (2006). Data Analysis Using Regression and
Multilevel/Hierarchical Models. Cambridge: Cambridge University
Press.

Leemann, A., Jeszenszky, P., Steiner, C., Studerus, M., &
Messerli, J. (2020). SDATS Corpus – Swiss German dialects across time
and space. Retrieved from osf.io/s9z4q
